# Visible-light-induced radical cascade cyclization: a catalyst-free synthetic approach to trifluoromethylated heterocycles

**DOI:** 10.3762/bjoc.20.12

**Published:** 2024-01-19

**Authors:** Chuan Yang, Wei Shi, Jian Tian, Lin Guo, Yating Zhao, Wujiong Xia

**Affiliations:** 1 College of Chemical and Material Engineering, Quzhou University, Quzhou 324000, China; 2 State Key Lab of Urban Water Resource and Environment, School of Science, Harbin Institute of Technology (Shenzhen), Shenzhen, 518055, China; 3 School of Chemistry and Chemical Engineering, Henan Normal University, Xinxiang, Henan 453007, China

**Keywords:** cascade reaction, indole derivatives, photocatalysis, radical chain process, trifluoromethylation

## Abstract

A visible-light-promoted research protocol for constructing dihydropyrido[1,2-*a*]indolone skeletons is herein described proceeding through a cascade cyclization mediated by trifluoromethyl radicals. This method allows the efficient synthesis of various indole derivatives without the need of photocatalysts or transition-metal catalysts. Mechanism experiments indicate that the process involves a radical chain process initiated by the homolysis of Umemoto's reagent. This straightforward method enables a rapid access to heterocycles containing a trifluoromethyl group.

## Introduction

**Figure 1:**
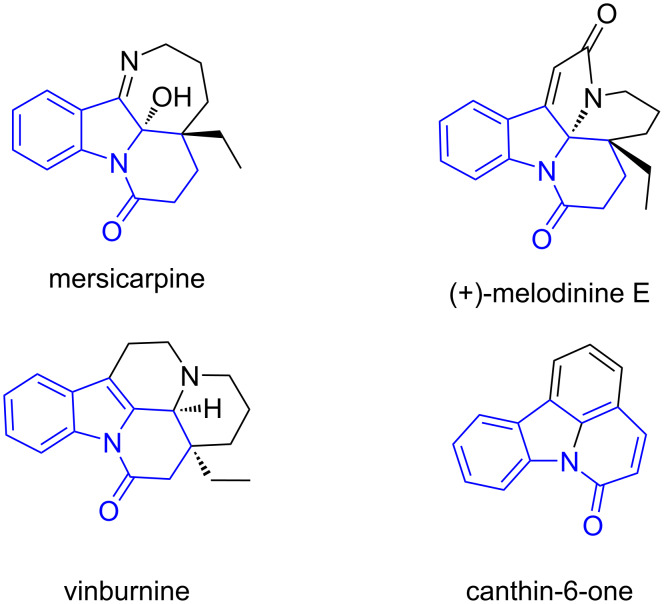
Representative dihydropyrido[1,2-*a*]indolone derivatives.

Dihydropyrido[1,2-*a*]indolone (DHPI) skeletons are commonly found in natural products and pharmaceutical compounds (Figure 1) [1–3], which exhibit a wide range of biological and pharmaceutical activities [4]. For example, mersicarpine has been found to inhibit protein translation and induce apoptosis [5] and vinburnine acts as a vasodilator for the treatment of cerebrovascular insufficiency [6–7].

**Scheme 1:**
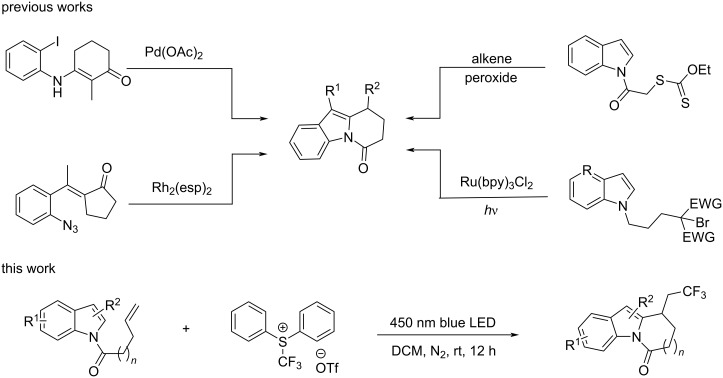
Selected works for the construction of dihydropyrido[1,2-*a*]indolones and current methodology.

Given their biological activity and potential applications, continuous efforts have been dedicated to the synthesis of DHPI derivatives. Various synthetic strategies have been explored (Scheme 1), including transition-metal-catalyzed cross-coupling reactions [8–10], annulation reaction of carbenoids [11], Friedel–Crafts acylation [12], radical cascade reactions [2,13], and photoinduced radical cyclizations [14–17]. However, these methods often suffer from drawbacks such as harsh reaction conditions and the requirement of transition-metal catalysts. Although photocatalyzed cyclization reactions usually occur under mild conditions, they typically require expensive metal-based photocatalysts or structurally complex organic dyes [18]. Therefore, the development of a photoinduced cascade reaction without the need of additional catalysts or additives remains highly desirable [19].

The introduction of an electron-withdrawing functional moiety into drug molecules would increase their metabolic stability [20], by avoiding, e.g., fast oxidation by cytochrome P450 oxidases [21]. In particular, the introduction of a trifluoromethyl group (–CF_3_) was shown to increase the metabolic stability of molecules and at the same time improved cell membrane permeability. Therefore, it became a commonly used strategy to modify medicine candidates [22–24]. Based on these advantages, we envisioned a one-step synthesis of dihydropyrido[1,2-*a*]indolone skeletons utilizing an indole substrate and a trifluoromethyl radical source under light irradiation. Umemoto’s reagent, which is capable of releasing a trifluoromethyl radical via a photoinduced single-electron-transfer (SET) process, is usually employed to enable the trifluoromethylation of unsaturated substrates [25–27]. Herein, we report a protocol to furnish trifluoromethylated dihyropyrido[1,2-*a*]indolones under mild conditions, without the need of photocatalysts or transition metals [28].

## Results and Discussion

We initialized our study by employing Ru(bpy)_3_Cl_2_·6H_2_O and Umemoto’s reagent to generate trifluoromethyl radicals via a photo-reductive quench process (Table 1). The indole substrate **1a** was chosen as a model substrate, and the reaction mixture was irradiated under 450 nm visible light for 12 h, resulting in the formation of the desired product **3a**, albeit in a relatively low yield (Table 1, entry 1). Control experiments revealed that omitting the photocatalyst led to an even higher yield (Table 1, entry 2), but light irradiation was essential to the reaction (Table 1, entries 3 and 4). Initially, some bases were added into the reaction system considering a deprotonation process, but subsequent investigations indicated that bases were not required. Among the solvents examined, DCM was found to be the most effective (Table 1, entries 5–8). However, due to the low boiling point of DCM, more solvent was added to avoid complete volatilization (Table 1, entry 8). The screening of different irradiation wavelengths revealed that 450 nm visible light irradiation is optimal (Table 1, entries 9–12). Furthermore, various types of Umemoto’s reagent were also screened (Table 1, entries 13–15). As Umemoto’s reagent **2b** was easier to prepare [29] and the use of **2b** did not significantly affect the reaction yield, it was chosen as the most suitable CF_3_ radical source. Further optimization involved screening the substrates’ ratios, which revealed that an excess of substrate **1a** resulted in improved yields. Finally, experimenting with anhydrous DCM as the solvent showed that anhydrous conditions were not necessary for the reaction (Table 1, entry 16).

**Table 1:** Optimization of reaction conditions.^a^

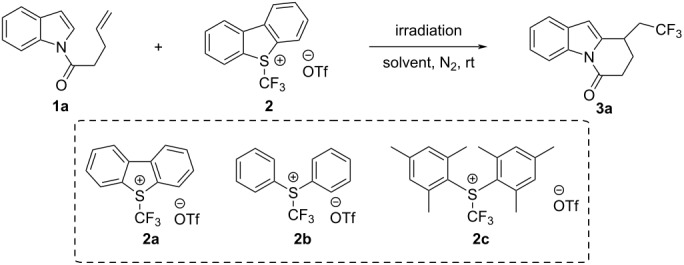						

Entry	CF_3_^·^ source (equiv)	Photocatalyst	Solvent	λ Irradiation (nm)	Time	Yield **3a**

1	**2a** (2)	Ru(bpy)_3_Cl_2_	MeCN	450	12 h	21%
2	**2a** (2)	–	MeCN	450	12 h	43%
3^b^	**2a** (2)	–	MeCN	450	12 h	52%
4^b^	**2a** (2)	–	MeCN	dark	12 h	trace
5^b^	**2a** (2)	–	1,4-dioxane	450	12 h	trace
6^b^	**2a** (2)	–	toluene	450	12 h	trace
7^b^	**2a** (2)	–	DCM (1 mL)	450	12 h	45%
8^b^	**2a** (2)	–	DCM (2 mL)	450	12 h	55%
9^b^	**2a** (2)	–	DCM (2 mL)	365	12 h	33%
10^b^	**2a** (2)	–	DCM (2 mL)	390	12 h	40%
11^b^	**2a** (2)	–	DCM (2 mL)	420	12 h	51%
12^c^	**2a** (1)	–	DCM (2 mL)	450	22 h	68%
13^b,c^	**2a** (1)	–	DCM (2 mL)	450	22 h	68%
14^c^	**2b** (1)	–	DCM (2 mL)	450	18 h	67%
15^c^	**2c** (1)	–	DCM (2 mL)	450	18 h	58%
16^c^	**2b** (1)	–	dry DCM	450	12 h	72%

^a^Standard reaction conditions: **1a** (0.1 mmol, 1.0 equiv), **2a** (0.2 mmol, 2.0 equiv), solvent (1 mL). ^b^2.0 equiv of KH_2_PO_4_ were added. ^c^3.0 equiv of substrate **1a** were utilized in the reaction.

**Scheme 2:**
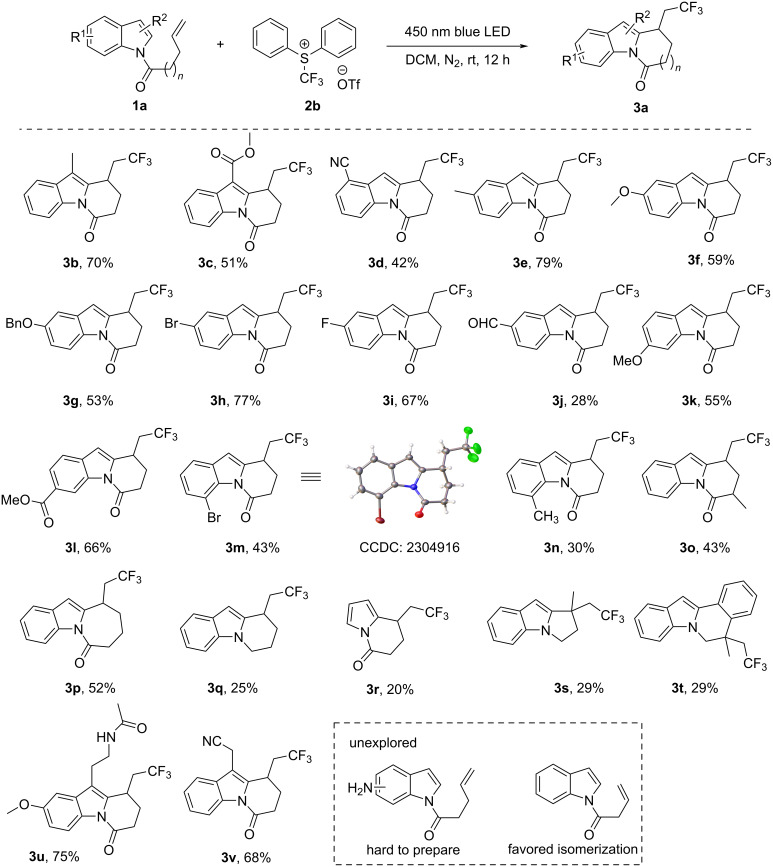
Substrate scope of the cascade reaction.

With the optimized conditions in hand, we started to explore the scope of this photoinduced transformation. Various alkene-tethered indole derivatives were subjected to the reaction. Remarkably, a range of dihydropyrido[1,2-*a*]indolones bearing a trifluoromethyl group were obtained in moderate to good yields (Scheme 2). In general, substrates with electron-withdrawing groups delivered the products in lower yields, such as -CN (**3d**, 42%) and -CHO (**3j**, 28%), while the substrates with electron-donating groups gave higher yields, such as -Me (**3b**, 70%; **3e**, 79%), -OMe (**3f**, 59%; **3k**, 55%). Most of the substrates bearing substituents at the C5 position on the indole skeleton reacted well, furnishing the products in moderate to good yields from 53–79% (**3e**–**i**) except the 5-CHO-substituted substrate which afforded product **3j** in 28% yield. Indoles with substituents at the C7 position of the indole ring (**3m** and **3n**) furnished the products in moderate yields. The structure of compound **3m** (4-Br) was confirmed by X-ray single crystal diffraction (CCDC: 2304916). Reactions of substrates with a longer carbon chain and a branched chain also proceeded well and afforded the products in 43% (**3o**) and 52% (**3p**) yields, respectively. If the chains did not involve a carbonyl group, the yields were much lower (**3q**, 25%; **3s**, 29%, and **3t**, 29%). When a pyrrole ring was used instead of indole, the reaction proceeded but gave the product in low yield (**3r**).

We did not explore the reaction with a primary amine functionalized substrate, because of competition of the reaction site during the synthesis of substrates. However, an amide-substituted indole furnished a clean product without competition; for example, product **3u** was derived from melatonin with an amide functional group, whose reaction to the desired product was clean without competitive byproducts. Finally, 3-indoleacetonitrile, a plant growth hormone, furnished the desired product in good yield (**3v**, 68%). Combined with melatonin, these examples demonstrate the suitability of our approach to be used in drug modification and development. In short, this reaction tolerates different substituents on the indole ring, including electron-donating and electron-withdrawing groups, providing access to a diverse array of dihydropyrido[1,2-*a*]indolone derivatives.

**Scheme 3:**
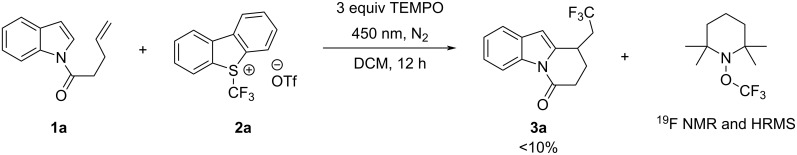
Radical trapping experiment.

To gain insight into the reaction process, we performed a series of control experiments. The addition of a typical radical scavenger – TEMPO (2,2,6,6-tetramethylpiperidin-1-yloxyl) significantly inhibited the reaction (as shown in Scheme 3), suggesting the involvement of radical species during the reaction process. Moreover, the radical trapping product was detected and confirmed via ^19^F NMR (Figure S3 in Supporting Information File 1) [30] and high-resolution mass spectrometry.

**Figure 2:**
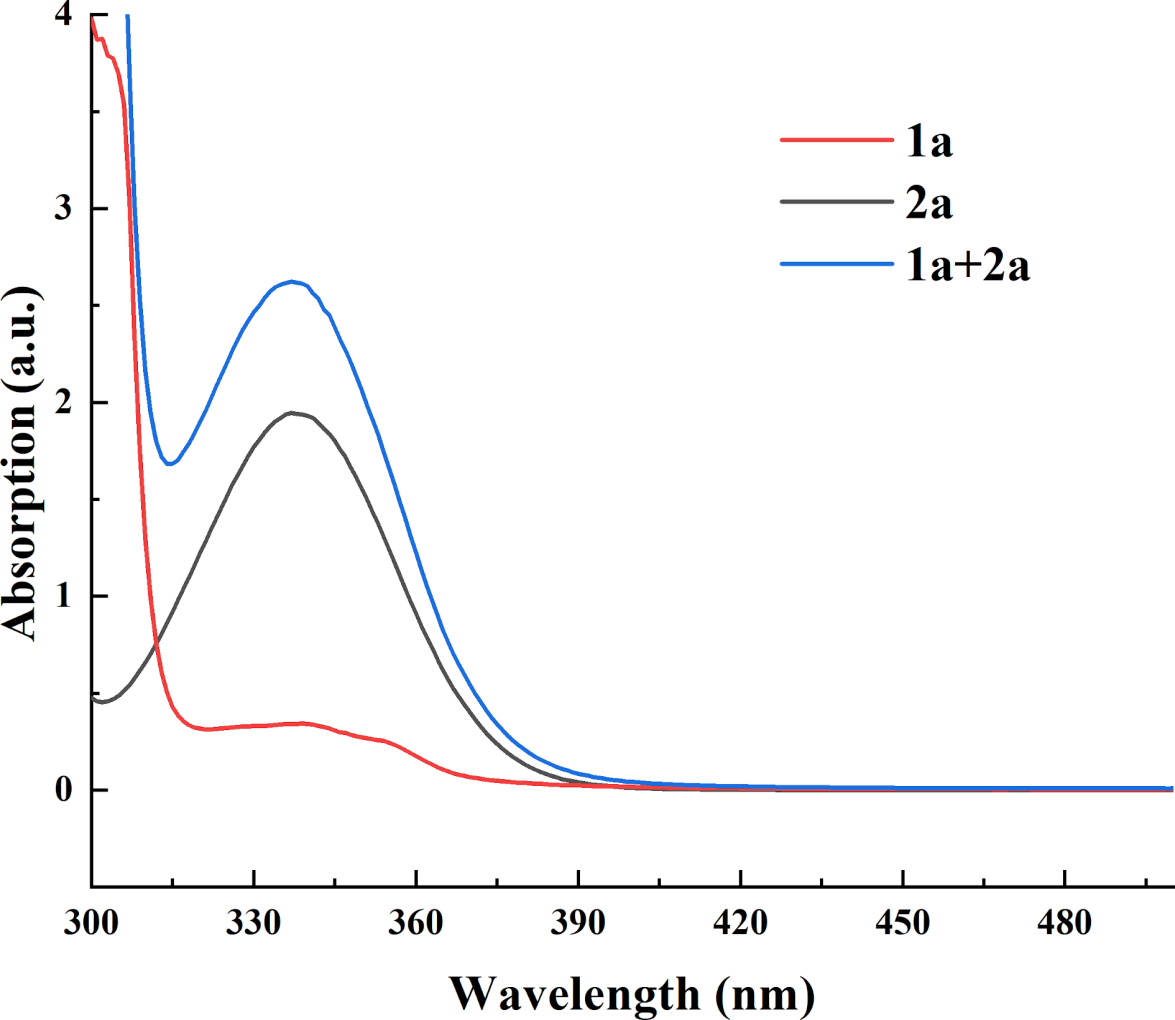
UV–vis spectra of substrates; [**1a**] 0.33 M, [**2a**] 0.11 M.

You and co-workers proposed a reaction pathway involving the combination of the indole substrate and Umemoto’s reagent to form an electron donor–acceptor (EDA) complex [31]. We excluded the possibility of an EDA charge transfer complex because there was no obvious EDA charge transfer band in the UV–vis spectra (Figure 2). Their indole substrate was more electron-rich in structure. The quantum yield was measured to investigate whether there was a radical chain process or not. The procedure was following a precedent work [32] (see Supporting Information File 1), and the calculated quantum yield was 2.2, which revealed that one photon generates more than one product molecule.

**Scheme 4:**
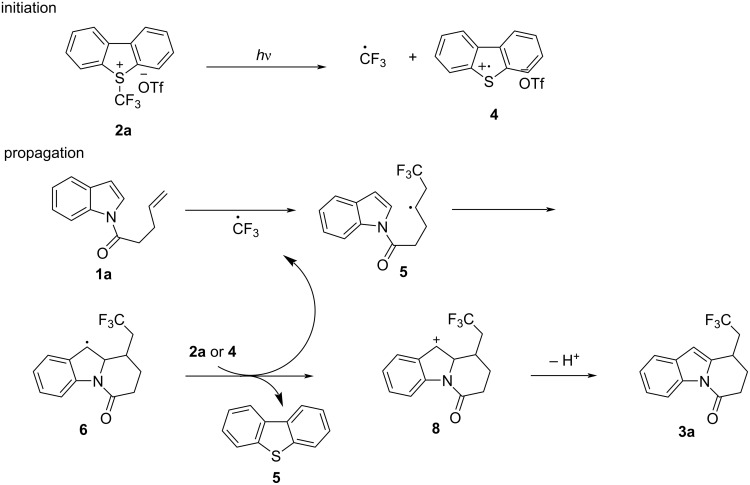
Plausible reaction mechanism.

Based on preliminary experiments and previous reports [33–34], we propose a plausible mechanism (Scheme 4). Upon light irradiation, Umemoto’s reagent undergoes a homolysis process to generate the trifluoromethyl radical species. The trifluoromethyl radical is trapped by the terminal alkene and forms a relayed radical intermediate **6**, which is intercepted by the indole ring realizing an intramolecular cyclization (6-*exo*-*trig*). The newly formed radical **7** can be oxidized by **2a** or **4** giving a cation **8**, which undergoes a deprotonation process and formation of the desired product.

## Conclusion

In conclusion, we have developed a visible-light-promoted protocol for the synthesis of dihydropyrido[1,2-*a*]indolones bearing a trifluoromethyl group at room temperature without additives. Mechanistic investigations support a photochemical process initiated by the homolysis of Umemoto's reagent under visible light irradiation. This method provides rapid access to a diverse range of trifluoromethylated dihydropyrido[1,2-*a*]indolone derivatives in moderate to good yields.

## Experimental

To a vial equipped with a stirring bar, alkene-tethered indole substrate **1a** (0.3 mmol), Umemoto's reagent (**2b**, 0.1 mmol), and DCM (2 mL) were added. Then, the vial was degassed and backfilled with N_2_ three times to remove oxygen. The reaction mixture was stirred at room temperature for 12 hours under visible light irradiation (450 nm). After completion, the reaction mixture was concentrated, and the crude product was purified by column chromatography to afford the desired dihydropyrido[1,2-*a*]indolone product.

## Supporting Information

File 1 Characterization data and copies of spectra.

File 2 Crystallographic information file (cif) of X-ray structure for compound **3m**.

## Data Availability

The data that supports the findings of this study is available from the corresponding author upon reasonable request.
