# Supplementary material for: Visible-light-induced radical cascade cyclization: a catalyst-free synthetic approach to trifluoromethylated heterocycles
Source: Beilstein J Org Chem. 2024 Jan 19;20:118–24. doi: 10.3762/bjoc.20.12 (PMC10804559; doi:10.3762/bjoc.20.12)

BEILSTEIN JOURNAL OF ORGANIC CHEMISTRY

Supporting Information

for

Visible-light-induced radical cascade cyclization: a catalyst-free synthetic approach to trifluoromethylated heterocycles

Chuan Yang, Wei Shi, Jian Tian, Lin Guo, Yating Zhao and Wujiong Xia

Beilstein J. Org. Chem. **2024,** *20,* 118–124. doi:10.3762/bjoc.20.12

Characterization data and copies of spectra

License and Terms: This is a supporting information file under the terms of the Creative Commons Attribution License (https://creativecommons.org/licenses/by/4.0). Please note that the reuse, redistribution and reproduction in particular requires that the author(s) and source are credited and that individual graphics may be subject to special legal provisions.
The license is subject to the *Beilstein Journal of Organic Chemistry* terms and conditions: (https://www.beilstein-journals.org/bjoc/terms)

Table of contents

1. Experimental section....S2
(a) General information....S2
(b) General procedures for the preparation of substances....S3
(c) General procedures....S4
(d) Mechanism exploration....S4
(e) Radical trap expriment....S5
(f) Details of quantum yield measurement....S8
(g) Crystal information of 3m 4-Br....S10
(h) References....S12
2. Characterization of products....S13
3. NMR spectra of products....S22

1. Experimental section

(a) General information

Petrol ether was redistilled to minimize the "oil peak" in ^{1}H NMR spectra around 1.2 ppm. Without specially stated, all commercially available reagents and solvents were used as received. Reactions were monitored by thin-layer chromatography (TLC) on commercial silica gel plates (GF254) using UV light for visualization. The photocatalytic reactions were performed in a reactor (WP-TEC-1020SL) purchased from WATTCAS, China. HRMS spectra were recorded on a Waters Xevo G2QTOF/UPLC mass spectrometer using electrospray ionization.

NMR tests were all performed on a Quantum-I Plus 400 spectrometer. All NMR tested samples used $CDCl_3$ with TMS internal standard as solvent. For ^{1}H NMR, chloroform-*d* (δ = 7.26 ppm) and tetramethylsilane (TMS, δ = 0 ppm) served as the internal standard; for ^{13}C NMR, vhloroform-*d* (δ = 77.16 ppm) served as the internal standard. Data are reported as follows: chemical shift (in ppm), multiplicity (s = singlet, d = doublet, t = triplet, q = quartet, p = quintet, hept = heptet, m = multiplet, br = broad), coupling constant (in Hz), and integration.

(b) General procedures for the preparation of substances

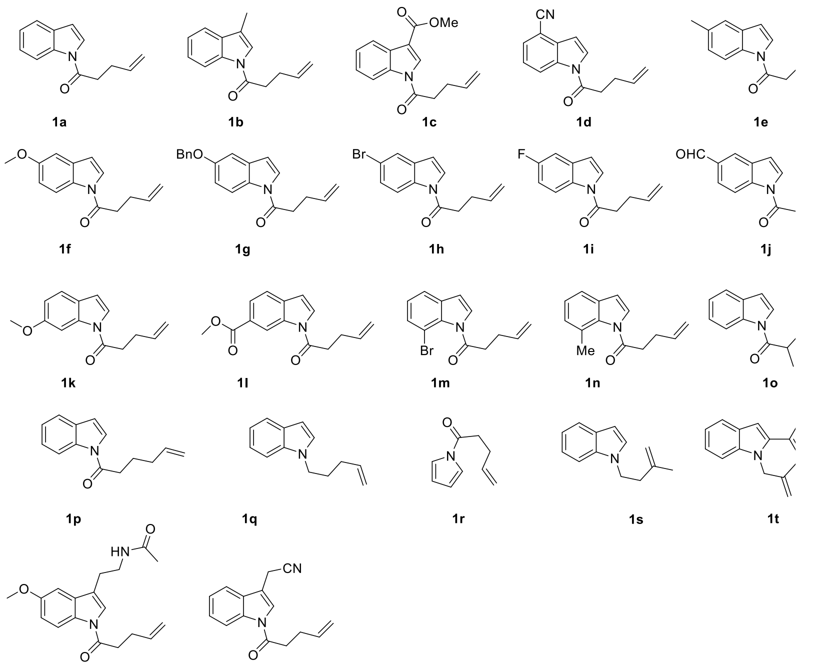

Figure S1 Substrates used in this work

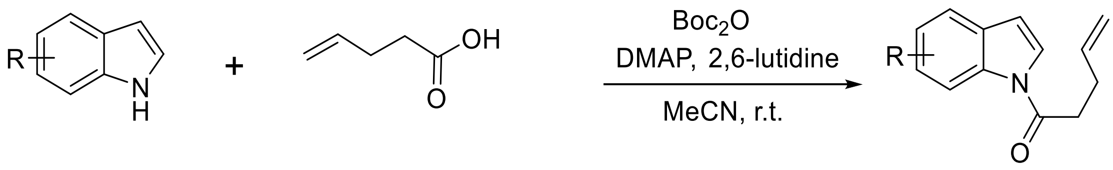

1a-**q**, **1s**, **1v**, and **1w** were prepared according to precedent literature[1]. Generally, 2.5 equiv indole, 1 equiv carboxylic acid chain, 2.5 equiv Boc_2O, 5 mol % DMAP, and 10 mol % 2,6-lutidine were added in 10 mL MeCN. Then, the mixture was stirred at room temperature for about 1 day to obtain the products in moderate to good yields. The unavoidable by-product is *N*-Bocindole. The desired products were separated using column chromatography with the appropriate eluent. The excess equivalent carboxylic could be removed under vacuum during the concentrating of the mixture.

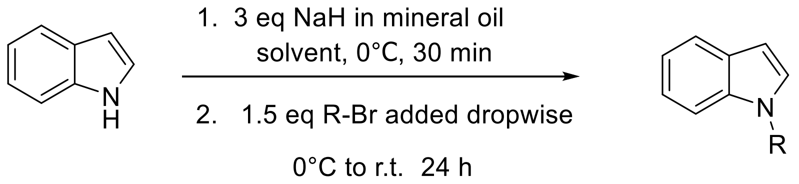

1r, **1t**, **1u** were prepared with the corresponding alkyl halide and indole derivatives[2]. At first, a strong base (NaH, 3 equiv) was added in THF or DMF solvent, stirred, and cooled to 0 °C. Then, the indole derivative was added and the mixture kept for 30 min. Then, the alkyl halide (e.g. 5-bromo-1-pentene, 1.5 equiv) was added dropwise at 0 °C. The reaction progress was monitored by TLC and generally the reaction mixture was stirred for 1 day before purification.

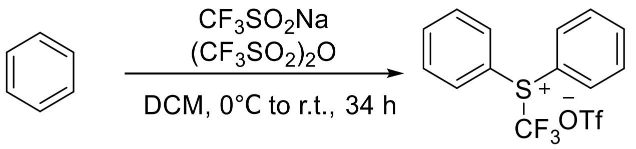

The preparation procedure of Umemoto's reagent was also reported in precedent work[3]. Specifically, 4 mL benzene were added into sodium trifluoromethyl sulfite solution (1.38 g in 10 mL DCM). Then, the mixture was stirred and cooled to 0 °C with an ice－water bath. After that, 3.4 mL trifluoromethyl sulfonic anhydride were added dropwise and the mixture was gradually warmed to room temperature and allowed to react for 34 h to get a moderate yield. After the reaction had finished, the mixture was diluted with DCM, and washed with saturated $NaHCO_3$ solution. The organic phase was collected and dried with anhydrous Na_2SO_4. Then, the solution was concentrated on a vacuum rotary evaporator. The product was purified by column chromatography (DCM/MeCN 4:1, v/v) to obtain 2 g of the product.

(c) General procedures

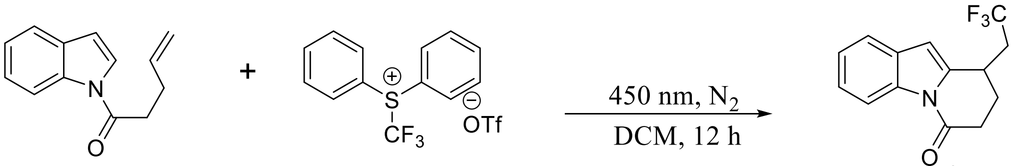

1a **2b** **3a**

Generally, Umemoto's reagent (40.3 mg, 1 mmol) and the N-substituted indole (60.3 mg, 3 equiv) were added into a quartz tube, which was charged with a stirring bar, followed by the addition of 1.5 mL anhydrous DCM. The tube was put into the photoreactor, degassed and backfilled with nitrogen gas with a vacuum pump and a N_2 balloon. The tube was irradiated for 12 hours. After that, the mixture was concentrated with a rotary evaporator and purified by column chromatography. The developing solvent was a mixture of petrol ether and ethyl acetate, and the ratio varied according to the product polarity.

(d) Mechanism exploration

In Zhang's work[4], the authors claimed the indole substrate may form oligomers serving as a photocatalyst under the reaction conditions, and we guess that it is likely that the oligomer was formed after one indole molecule lost an electron, and was attacked by another indole nucleophile.

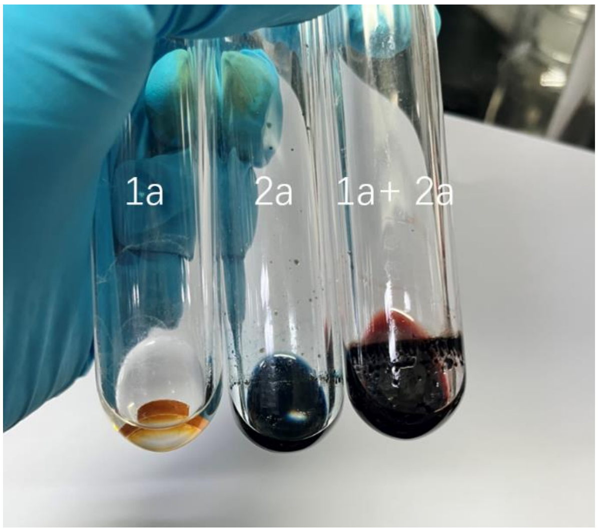

Figure S2 Picture of the control experiment (after 12 h of irradiation)

We did not observe precipitate formation when irradiating an indole (**1a**) solution for 12 h or Umemoto's reagent (**2a**), although under these conditions the S–CF_3 bond would be broken. However, using a mixture of **1a** and **2a**, precipitates formed after the reaction. These precipitates could not be investigated to determine structures due to the poor solubility in DMSO-d_6 or $CDCl_3$. We guess these were oligomers of the indole substrate

(e) Radical trap expriment

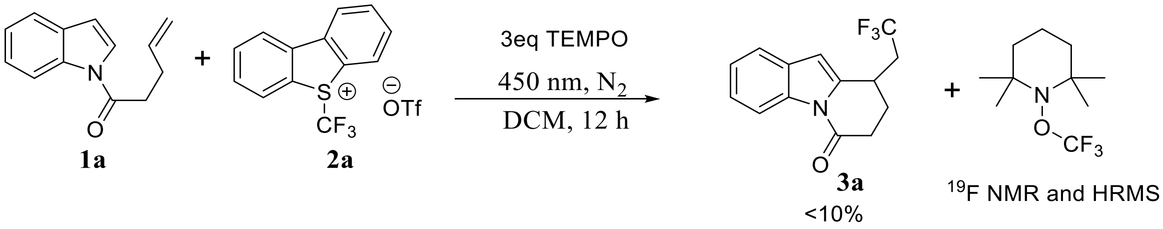

The trapped product could not be isolated because of no UV absorption above 254 nm and no reaction with common indicators. So we used chromatography to isolate and collected the part before the product to perform an NMR test. The collected part after condensation was still a mixture, so we just put its ^{19}F NMR result here.

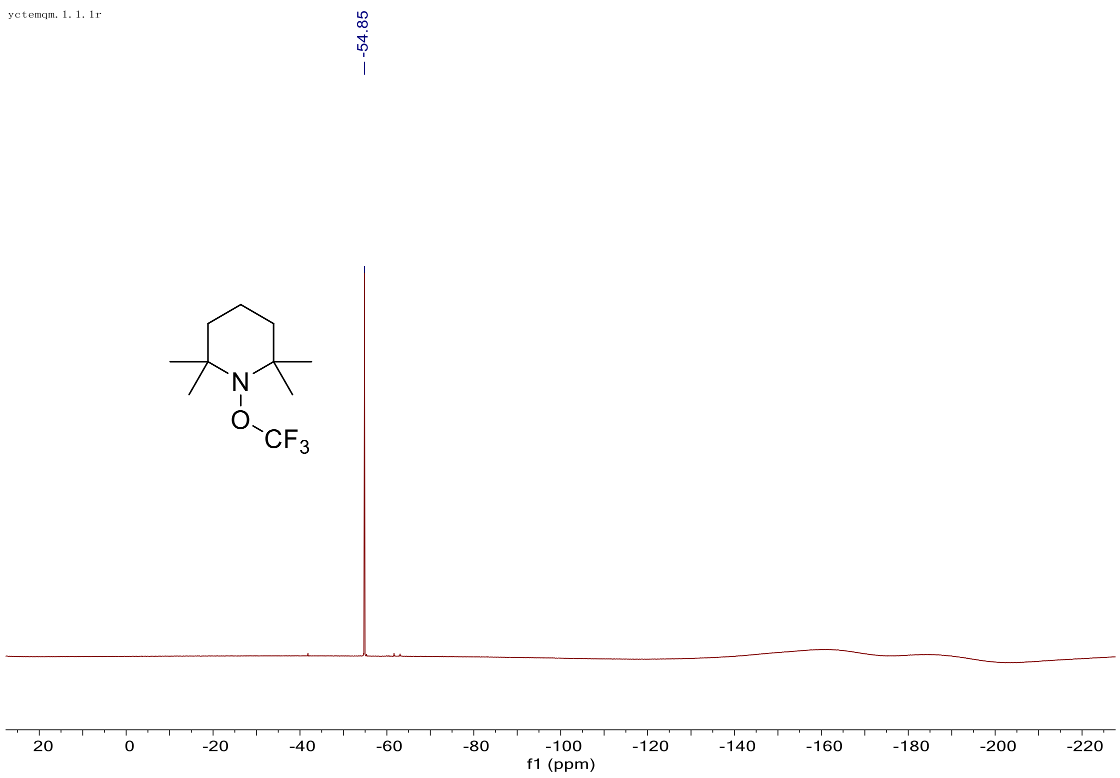

Figure S3 ^{19}F NMR of trapped product

^{19}F NMR (376 MHz, $CDCl_3$) δ −54.85 ppm. The peak position was verified with a precedent report[5], though its position was −55.71 ppm.

Besides, we used GCMS to analyze the components in the system. The trapped product has an *m*/*z* 225.13 molecular ion peak, which could be seen in the GCMS spectrum and its HRMS result also supported its existence. HRMS $[M + H]^+$ calcd for 226.1413; found: 226.1419.

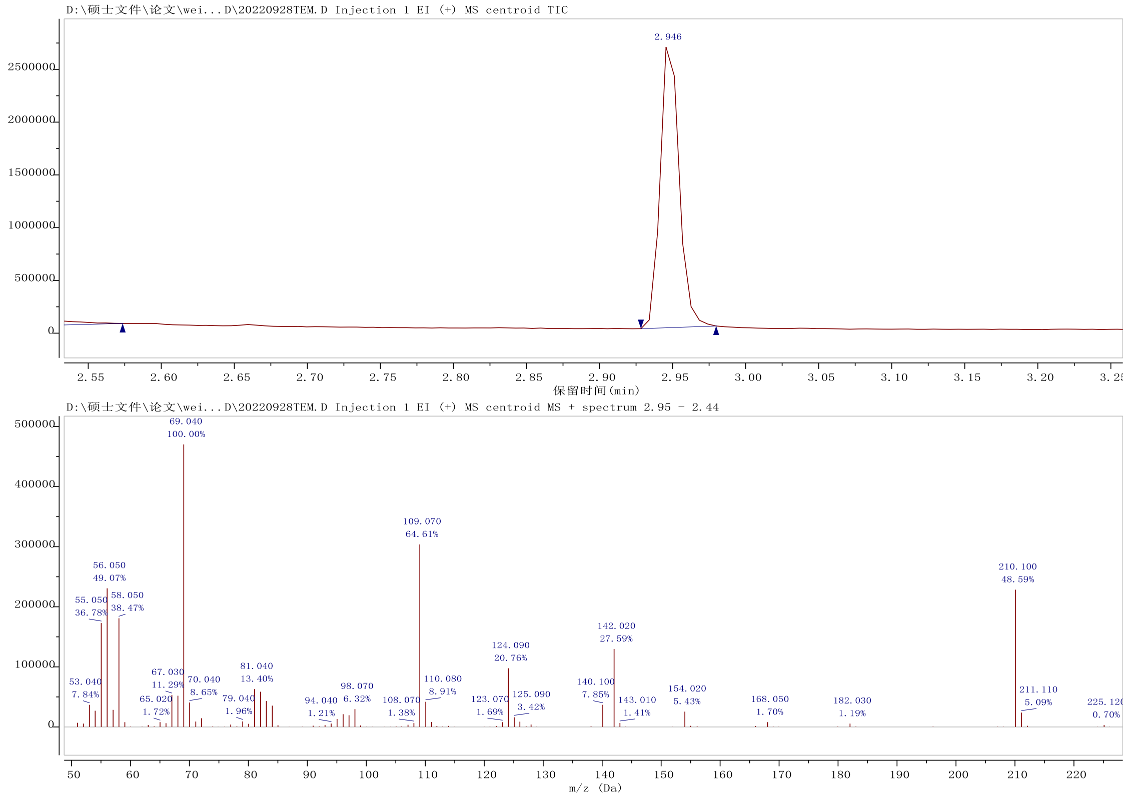

Figure S4 GCMS result of the trapped product

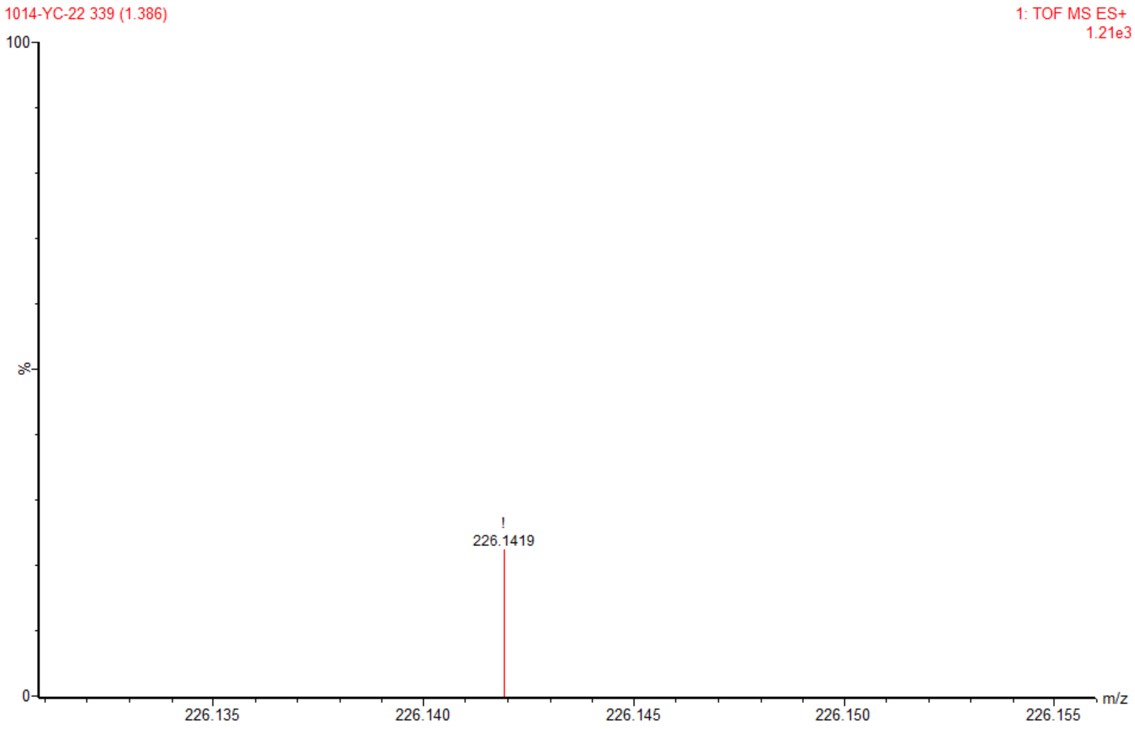

Figure S5 HRMS of trapped product $[M + H]^+$

(f) Details of quantum yield measurement

Determination of light intensity 450 nm LED:

The method to measure the quantum yield was according to Yoon's work[6]. The solutions were prepared following their report and experiments were performed as reported. The mol of Fe^{2+} was calculated using Eq 1:

$$\text{mol Fe}^{2+} = \frac{V*\Delta A}{l*\varepsilon} \tag{1}$$

V is the volume of the solvent(0.003 L); delta A is the difference in absorptions of the irradiated sample and unirradiated sample (1.989) at 510 nm; *l* is the path length (1 cm), and ε is the molar absorptivity at 510 nm (11100 L mol^{-1} cm^{-1}). The photon flux can be calculated via Eq 2.

$$\text{photon flux} = \frac{\text{mol Fe}^{2+}}{\Phi*t*f}$$

(2)

Φ is the quantum yield of Fe^{2+} at 450 nm (approximately 0.96 at 450 nm for a 0.15 M solution); *t* is time (90 s), and f is the fraction of light absorbed at 450 nm(f = $1-10^{-A}$, A = 2.41 at 450 nm, f = 0.996).

The photon flux was calculated to be $6.24\cdot10^{-9}$ einstein s^{-1}.

$$\text{mol Fe}^{2+} = \frac{0.003\ \text{L}*1.989}{1\ \text{cm}*11100\ \text{L mol}^{-1}\text{cm}^{-1}} = 5.375*10^{-7}\ \text{mol}$$

$$\text{photon flux} = \frac{5.375*10^{-7}}{0.96*90\text{s}*0.996} = 6.247*10^{-9}\ \text{einstein s}^{-1}$$

Determination of the reaction yield:

Experiment: 40 mg of Umemoto's reagent (0.1 mmol), 58 mg of **1a** (0.3 mmol), 1.5 mL DCM, N_2, 450 nm LED, irradiated for 35 min.

There was 0.029 mmol product formed (NMR yield with 4-chlorobenzotrifluoride as internal standard). Before the NMR test, 8.5 mL 4-chlorobenzotrifluoride were added.

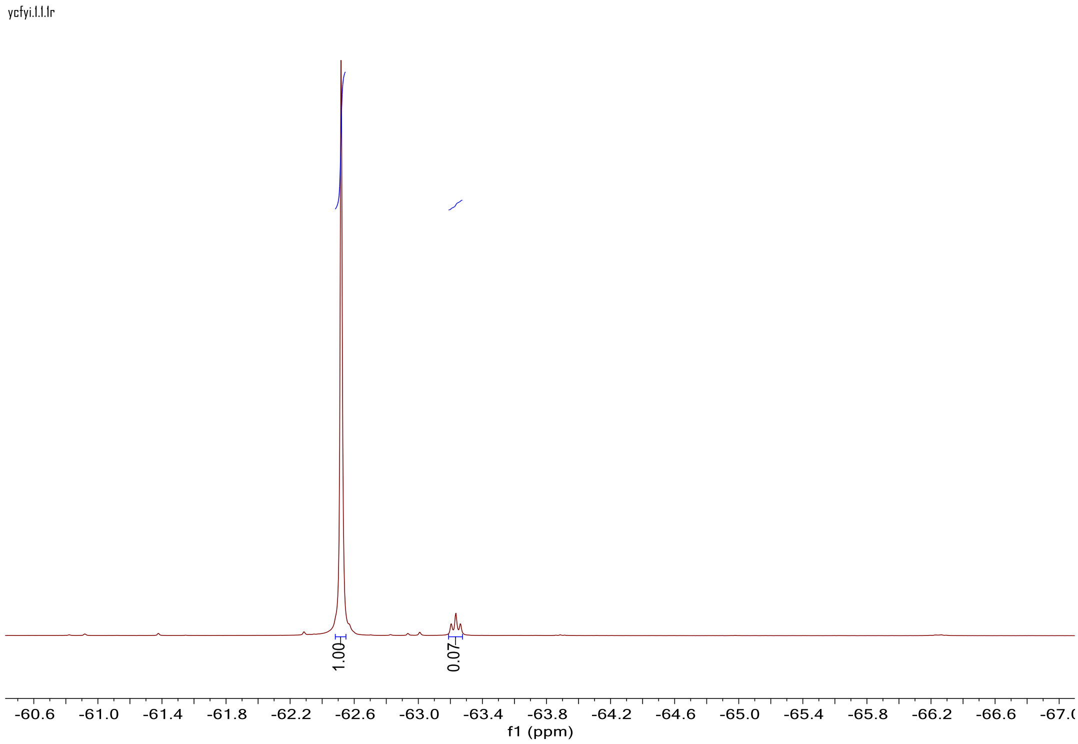

Figure S6 ^{19}F NMR yield of reaction after 35 min

According to the definition of quantum yield, the yield was calculated to be 2.24. f is the fraction of the absorbed light by the mixture and f is calculated to be 1 at half of reaction concentration under light irradiation for 30 min.

$$\text{quantum yield} = \frac{2.9*10^{-5}}{6.247*10^{-9}*2100*0.99} = 2.24$$

$$f = 1- 10^{-A} = 1-10^{-2} = 0.99$$

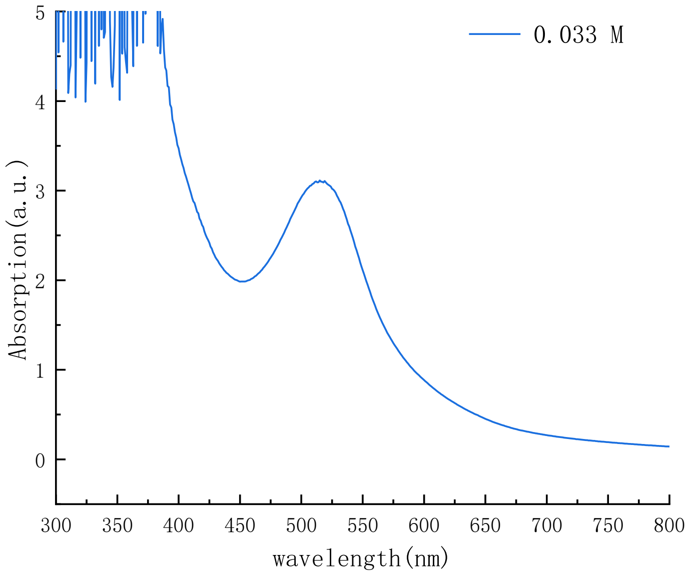

Figure S7 UV–vis absorption of reaction mixture irradiated for 30 min.

(g) Crystal information of 3m 4-Br

Experimental

Single crystals of $C_{14}H_{11}BrF_3NO$ **3m** were obtained by slow evaporation of solvent at room temperature. A suitable crystal was selected and put on an XtaLAB PRO MM007-DW diffractometer. The crystal was kept at 273.15 K during data collection. The X-ray source is Cu Kα (λ = 1.54178 Å). Using Olex2 [1], the structure was solved with the SHELXS [2] structure solution program using Direct Methods and refined with the SHELXL [3] refinement package using least squares minimization.

1. Dolomanov, O.V., Bourhis, L.J., Gildea, R.J, Howard, J.A.K. & Puschmann, H. (2009), J. Appl. Cryst. 42, 339-341.
2. Sheldrick, G.M. (2008). Acta Cryst. A64, 112-122.
3. Sheldrick, G.M. (2015). Acta Cryst. C71, 3-8.

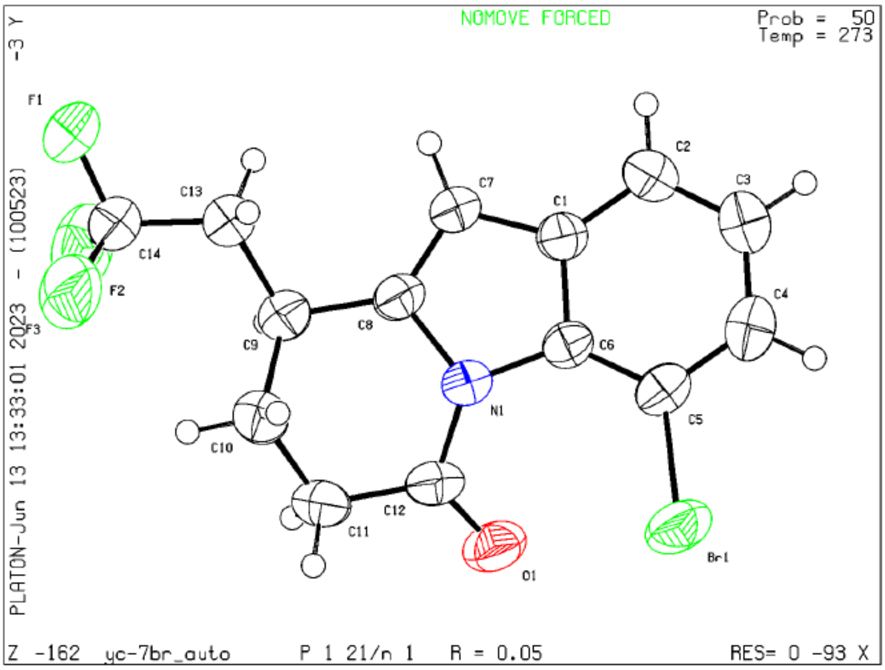

Figure S8 Ellipsoid model of **3m**

Table 1 Crystal data and structure refinement for yc-4Br_auto.

Identification code	yc-4Br_auto
Empirical formula	$C_{14}H_{11}BrF_3NO$
Formula weight	346.15
Temperature/K	273.15
Crystal system	monoclinic
Space group	$P2_1/n$
a/Å	12.6437(2)
b/Å	8.2123(2)
c/Å	13.1511(2)
α/°	90
β/°	106.061(2)
γ/°	90
Volume/Å^3	1312.23(4)
Z	4
ρ_{calc}g/cm^3	1.752
μ/mm^{-1}	4.584
F(000)	688.0
Crystal size/mm^3	0.15 × 0.13 × 0.12
Radiation	Cu Kα (λ = 1.54184)
2Θ range for data collection/°	8.588 to 153.178
Index ranges	$-15 \leq h \leq 15$, $-3 \leq k \leq 9$, $-16 \leq l \leq 16$
Reflections collected	8179
Independent reflections	2608 [R_{int} = 0.0379, R_{sigma} = 0.0315]
Data/restraints/parameters	2608/0/182
Goodness-of-fit on F^2	1.076
Final R indexes [I>=2σ (I)]	R_1 = 0.0475, wR_2 = 0.1381
Final R indexes [all data]	R_1 = 0.0514, wR_2 = 0.1443
Largest diff. peak/hole / e Å^{-3}	0.81/-0.59

Crystal structure determination of 3m 4Br

Crystal data for $C_{14}H_{11}BrF_3NO$ (M =346.15 g/mol): monoclinic, space group $P2_1/n$ (no. 14), a = 12.6437(2) Å, b = 8.2123(2) Å, c = 13.1511(2) Å, β = 106.061(2), V = 1312.23(4) Å^3, Z = 4, T = 273.15 K, μ(Cu Kα) = 4.584 mm^{-1}, D_{calc} = 1.752 g/cm^3, 8179 reflections measured (8.588° ≤ 2Θ ≤ 153.178°), 2608 unique (R_{int} = 0.0379, R_{sigma} = 0.0315) which were used in all calculations. The final R_1 was 0.0475 (I > 2σ(I)) and wR_2 was 0.1443 (all data).

(h) References

[1] UMEHARA A, UEDA H, TOKUYAMA H. Condensation of Carboxylic Acids with Non-Nucleophilic N-Heterocycles and Anilides Using Boc2o[J]. J Org Chem, 2016, 81(22): 11444-11453.

[2] OLIVIER W J, GARDINER M G, BISSEMBER A C, et al. Brønsted Acid-Mediated Annulations of Pyrroles Featuring N-Tethered A,B-Unsaturated Ketones and Esters: Total Syntheses of (±)-Tashiromine and (±)-Indolizidine 209i[J]. Tetrahedron, 2018, 74(38): 5436-5441.

[3] WANG S M, WANG X Y, QIN H L, et al. Palladium-Catalyzed Arylation of Arylboronic Acids with Yagupolskii-Umemoto Reagents[J]. Chemistry, 2016, 22(19): 6542-6546.

[4] YANG R, YI D, SHEN K, et al. Indole and Pyrrole Derivatives as Pre-Photocatalysts and Substrates in the Sulfonyl Radical-Triggered Relay Cyclization Leading to Sulfonylated Heterocycles[J]. Org Lett, 2022, 24(10): 2014-2019.

[5] WANG X, YE Y, ZHANG S, et al. Copper-Catalyzed C(Sp3)–C(Sp3) Bond Formation Using a Hypervalent Iodine Reagent: An Efficient Allylic Trifluoromethylation[J]. Journal of the American Chemical Society, 2011, 133(41): 16410-16413.

[6] CISMESIA M A, YOON T P. Characterizing Chain Processes in Visible Light Photoredox Catalysis[J]. Chemical Science, 2015, 6(10): 5426-5434.

[7] ZHOU Q-H, DAI J-Y, ZHAO W-J, et al. Photocatalytic Synthesis of Azaheterocycle-Fused Piperidines and Pyrrolidines Via Tandem Difunctionalization of Unactivated Alkenes[J]. Organic & Biomolecular Chemistry, 2023, 21(16): 3317-3322.

[8] CHENG J, ZHANG H, LV J, et al. Palladium-Catalyzed Intermolecular Dicarbofunctionalization of Unactivated Alkenes: Synthesis of Fluoroalkylated Heterocycles with All-Carbon Quaternary Centers[J]. European Journal of Organic Chemistry, 2022, 2022(3): e202101342.

2. Characterization of products

9-(2,2,2-Trifluoroethyl)-8,9-dihydropyrido[1,2-*a*]indol-6(7*H*)-one (3a)

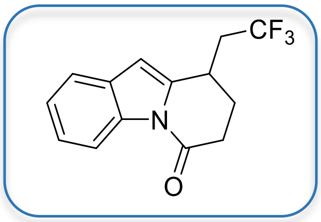

3a, white solid, 19.5mg, 72%. **^{1}H NMR** (400 MHz, $CDCl_3$) δ 8.47 (d, J = 8.0 Hz, 1H), 7.50 (d, J = 7.2 Hz, 1H), 7.36 – 7.27 (m, 2H), 6.41 (s, 1H), 3.37 (dq, J = 9.2, 4.8, 4.2 Hz, 1H), 2.98 – 2.75 (m, 3H), 2.52 – 2.32 (m, 2H), 2.04 – 1.88 (m, 1H). **^{13}C NMR** (101 MHz, $CDCl_3$) δ 168.57, 139.31, 135.22, 129.18, 126.40 (d, J = 277.5 Hz), 124.96, 124.29, 120.17, 116.56, 105.18, 77.48, 77.16, 76.84, 37.37 (q, J = 28.4 Hz)., 29.44, 29.41, 26.95. **^{19}F NMR** δ -63.27 (t, J = 10.9 Hz). **HRMS (ESI)**: calcd. for $C_{14}H_{13}F_3NO[M+H]^+$: 268.0944; found: 268.0943.

10-Methyl-9-(2,2,2-trifluoroethyl)-8,9-dihydropyrido[1,2-*a*]indol-6(7*H*)-one (3b)

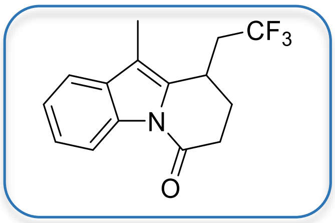

3b, white solid, 19.7mg, yield 70%. **^{1}H NMR** (400 MHz, $CDCl_3$) δ 8.45 (d, J = 8.2 Hz, 1H), 7.46 (d, J = 7.5 Hz, 1H), 7.32 (p, J = 7.3 Hz, 2H), 3.68 (d, J = 4.4 Hz, 1H), 2.95 – 2.74 (m, 2H), 2.58 – 2.27 (m, 2H), 2.24 (d, J = 6.7 Hz, 5H). **^{13}C NMR** (101 MHz, $CDCl_3$) δ 168.1, 134.6, 133.9, 130.7, 126.2 (d, J = 277.5 Hz), 125.1, 124.1, 118.3, 116.6, 113.6, 77.5, 77.2, 76.8, 36.3 (q, J = 27.6 Hz), 29.8, 29.4, 25.4, 25.4, 24.6, 8.5. **^{19}F NMR** (376 MHz, $CDCl_3$) δ -64.18 (t, J = 10.8 Hz). **HRMS (ESI)**: calcd. for $C_{15}H_{15}F_3NO[M+H]^+$: 282.1100; found: 283.1102.

10-Methyl-9-(2,2,2-trifluoroethyl)-8,9-dihydropyrido[1,2-*a*]indol-6(7*H*)-one (3b)

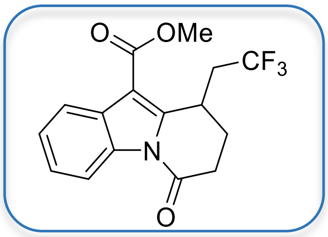

3c, white solid, 16.6mg, yield 51%. **^{1}H NMR** (400 MHz, $CDCl_3$) δ 8.53 – 8.47 (m, 1H), 8.15 – 8.09 (m, 1H), 7.42 – 7.36 (m, 2H), 4.46 (dd, J = 11.0, 5.5 Hz, 1H), 3.99 (d, J =

1.0 Hz, 3H), 2.99 – 2.86 (m, 2H), 2.75 – 2.61 (m, 1H), 2.49 (ddd, J = 15.0, 11.2, 9.6 Hz, 1H), 2.41 – 2.32 (m, 1H), 2.26 (ddt, J = 14.2, 9.0, 5.2 Hz, 1H). **^{13}C NMR** (101 MHz, $CDCl_3$) δ 168.65, 164.74, 146.64, 134.64, 126.77, 126.04 (d, J = 278.0 Hz), 125.92, 125.31, 121.56, 116.54, 109.21, 77.48, 77.16, 76.85, 51.71, 34.88 (q, J = 27.9 Hz), 29.33, 27.15, 27.12, 22.73. **^{19}F NMR** (376 MHz, $CDCl_3$) δ -63.62 (t, J = 10.8 Hz). **HRMS (ESI)**: calcd. for $C_{16}H_{15}F_3NO_3[M+H]^+$: 326.0999; found: 326.0997.

6-Oxo-9-(2,2,2-trifluoroethyl)-6,7,8,9-tetrahydropyrido[1,2-*a*]indole-1-carbonitrile (3d)

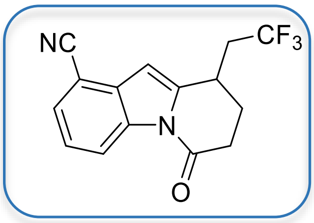

3d, white solid,12.3 mg, 42%. **^{1}H NMR** (400 MHz, $CDCl_3$) δ 8.68 (d, J = 8.6 Hz, 1H), 7.59 (d, J = 7.6 Hz, 1H), 7.38 (t, J = 8.0 Hz, 1H), 6.65 (s, 1H), 3.44 (dt, J = 10.0, 5.2 Hz, 1H), 3.01 – 2.81 (m, 3H), 2.58 – 2.39 (m, 2H), 2.01 (dtd, J = 13.6, 11.1, 4.5 Hz, 1H). **^{13}C NMR** (101 MHz, $CDCl_3$) δ 168.55, 142.48, 135.02, 131.29, 128.46, 126.17 (q, J = 277.4 Hz) 124.91, 124.79, 124.05, 121.01, 117.68, 103.35, 77.48, 77.16, 76.84, 37.19 (q, J = 28.9 Hz), 32.93, 31.50, 30.25, 29.61, 26.71. **^{19}F NMR** (376 MHz, $CDCl_3$) δ -63.23 (t, J = 10.7 Hz). **HRMS (ESI)**: calcd. for $[M+H]^+$: 286.0850; found: 268.0893.

2-Methyl-9-(2,2,2-trifluoroethyl)-8,9-dihydropyrido[1,2-*a*]indol-6(7*H*)-one (3e)

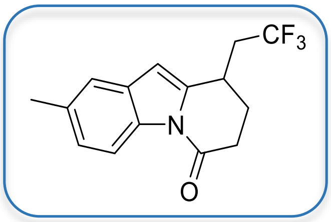

3e, white solid, 22.2mg, 79%. **^{1}H NMR** (400 MHz, $CDCl_3$) δ 8.32 (d, J = 8.4 Hz, 1H), 7.28 (s, 1H), 7.14 (d, J = 8.3 Hz, 1H), 6.31 (s, 1H), 3.33 (dt, J = 9.7, 5.2 Hz, 1H), 2.92 – 2.72 (m, 3H), 2.44 (s, 3H), 2.43 – 2.31 (m, 2H), 1.93 (td, J = 13.4, 12.1, 4.5 Hz, 1H). **^{13}C NMR** (101 MHz, $CDCl_3$) δ 168.39, 139.34, 133.89, 133.37, 129.42, 126.43 (q, J = 277.2 Hz), 126.16, 120.16, 116.15, 104.94, 77.47, 77.16, 76.74, 37.33 (q, J = 28.2 Hz), 32.87, 31.56, 30.20, 29.76, 29.41, 29.38, 26.96, 21.48. **^{19}F NMR** (376 MHz, $CDCl_3$) δ -63.26 (t, J = 10.9 Hz). **HRMS (ESI)**: calcd. for $C_{15}H_{15}F_3NO[M+H]^+$: 282.1100; found: 282.1100.

2-Methoxy-9-(2,2,2-trifluoroethyl)-8,9-dihydropyrido[1,2-*a*]indol-6(7*H*)-one (3f)

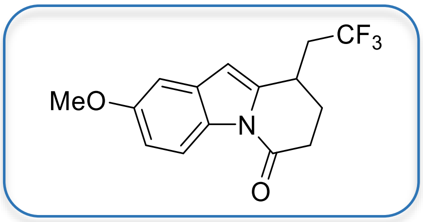

3f, white solid, 17.5mg, yield 59%. **^{1}H NMR** (400 MHz, $CDCl_3$) δ 8.33 (d, J = 8.9 Hz, 1H), 6.99 – 6.87 (m, 2H), 6.33 – 6.29 (m, 1H), 3.84 (s, 3H), 3.33 (dt, J = 9.7, 5.2 Hz, 1H), 2.91 – 2.71 (m, 3H), 2.46 – 2.30 (m, 2H), 1.98 – 1.87 (m, 1H). **^{13}C NMR** (101 MHz, $CDCl_3$) δ 168.19, 156.93, 140.04, 130.25, 129.83, 126.42 (q, J = 277.4 Hz)117.27, 112.86, 105.05, 103.31, 77.47, 77.16, 76.84, 55.70, 37.30 (q, J = 28.3 Hz), 32.75, 29.43, 29.40, 29.37, 29.34, 27.00. **^{19}F NMR** (376 MHz, $CDCl_3$) δ -63.26 (t, J = 10.9 Hz). **HRMS (ESI)**: calcd. for $C_{15}H_{15}F_3NO_2[M+H]^+$: 298.1049; found: 268.1055.

2-(Benzyloxy)-9-(2,2,2-trifluoroethyl)-8,9-dihydropyrido[1,2-*a*]indol-6(7*H*)-one (3g)

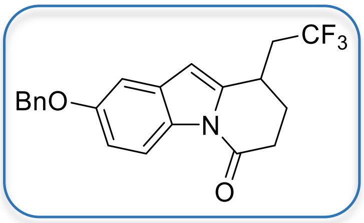

3g, white solid, 19.8 mg, yield 53%. **^{1}H NMR** (400 MHz, $CDCl_3$) δ 8.40 – 8.31 (m, 1H), 7.46 (d, J = 7.3 Hz, 2H), 7.39 (t, J = 7.3 Hz, 2H), 7.33 (t, J = 7.2 Hz, 1H), 7.05 – 6.98 (m, 2H), 6.30 (s, 1H), 5.10 (s, 2H), 3.32 (dt, J = 9.5, 5.1 Hz, 1H), 2.92 – 2.68 (m, 3H), 2.48 – 2.30 (m, 2H), 2.00 – 1.85 (m, 1H). **^{13}C NMR** (101 MHz, $CDCl_3$) δ 168.17, 156.09, 140.07, 137.22, 130.23, 130.01, 128.66, 128.01, 127.56, 126.42 (q, J = 277.2 Hz), 117.32, 113.73, 105.07, 104.67, 77.48, 77.16, 76.84, 70.56, 45.70, 37.30 (q, J = 28.3 Hz), 32.77, 29.79, 29.43, 29.41, 29.38, 29.35, 27.00. **^{19}F NMR** (376 MHz, $CDCl_3$) δ -63.23 (t, J = 10.9 Hz). **HRMS (ESI)**: calcd. for $C_{21}H_{19}F_3NO_2[M+H]^+$: 374.1362; found: 374.1363.

2-Bromo-9-(2,2,2-trifluoroethyl)-8,9-dihydropyrido[1,2-*a*]indol-6(7*H*)-one (3h)

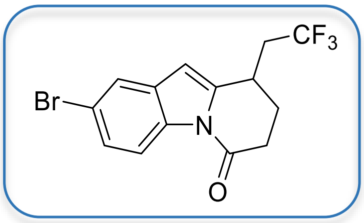

3h, white solid, 26.6 mg, yield 77%. **^{1}H NMR** (400 MHz, $CDCl_3$) δ 8.32 (d, J = 8.7 Hz, 1H), 7.61 (s, 1H), 7.41 (d, J = 8.7 Hz, 1H), 6.34 (s, 1H), 3.37 (dt, J = 9.6, 5.1 Hz, 1H), 2.94 – 2.76(m, 3H), 2.50 – 2.35 (m, 2H), 2.05 – 1.88 (m, 1H). **^{13}C NMR** (101 MHz, $CDCl_3$) δ 168.43, 140.62, 133.88, 130.95, 127.74, 126.30 (q, J = 277.6 Hz), 122.90, 117.89, 117.55, 104.32, 77.48, 77.16, 76.82, 37.27 (q, J = 28.5 Hz), 32.86, 29.77, 29.42, 29.39, 26.86. **^{19}F NMR** (376 MHz, $CDCl_3$) δ -63.25 (t, J = 10.8 Hz). **HRMS (ESI)**: calcd. for $C_{14}H_{12}BrF_3NO[M+H]^+$: 346.0049; found: 346.0049.

2-Fluoro-9-(2,2,2-trifluoroethyl)-8,9-dihydropyrido[1,2-*a*]indol-6(7*H*)-one (3i)

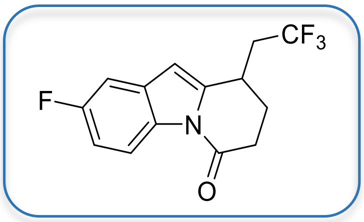

3i, white solid, 19.1 mg, yield 67%. **^{1}H NMR** (400 MHz, $CDCl_3$) δ 8.39 (dd, J = 9.0, 4.8 Hz, 1H), 7.19 – 6.94 (m, 2H), 6.35 (s, 1H), 3.36 (dt, J = 9.6, 5.1 Hz, 1H), 2.94 – 2.74 (m, 3H), 2.51 – 2.32 (m, 2H), 2.05 – 1.84 (m, 1H). **^{13}C NMR** (101 MHz, $CDCl_3$) δ 168.32, 161.23, 158.84, 141.01, 131.53, 130.34, 130.24, 126.34 (q, J = 277.2 Hz),117.58, 117.49, 112.54, 112.29, 106.04, 105.80, 104.86, 104.83, 77.48, 77.16, 76.84, 37.25 (q, J = 28.5 Hz), 32.76, 29.47, 29.44, 29.41, 29.38, 26.92.

^{19}F NMR (376 MHz, $CDCl_3$) δ -63.28 (t, J = 10.8 Hz), -118.43 (td, J = 8.9, 4.9 Hz). **HRMS (ESI)**: calcd. for $C_{14}H_{12}F_4NO[M+H]^+$: 286.0850; found: 268.0850.

6-Oxo-9-(2,2,2-trifluoroethyl)-6,7,8,9-tetrahydropyrido[1,2-*a*]indole-2-carbaldehyde (3j)

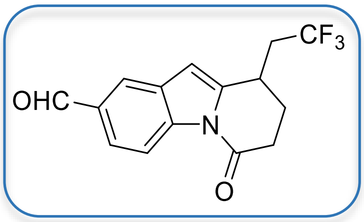

3j, white solid, 8.3mg, yield 28%. **^{1}H NMR** (400 MHz, $CDCl_3$) δ 10.06 (s, 1H), 8.60 (d, J = 8.5 Hz, 1H), 8.03 (s, 1H), 7.85 (d, J = 8.5 Hz, 1H), 6.53 (s, 1H), 3.41 (dt, J = 9.9, 5.2 Hz, 1H), 3.01 – 2.78 (m, 3H), 2.58 – 2.37 (m, 2H), 2.13 – 1.92 (m, 1H). **^{13}C NMR** (101 MHz, $CDCl_3$) δ 192.13, 168.67, 141.34, 138.72, 132.94, 129.47, 126.51, 126.26 (q, J = 277.4 Hz), 124.04, 122.63, 119.15, 116.92, 105.48, 77.47, 77.16, 76.84, 37.28 (q, J = 28.4 Hz), 32.99, 31.49, 30.24, 29.75, 29.53, 26.71. **^{19}F NMR** (376 MHz,

$CDCl_3$) δ -63.23, -63.25, -63.28. **HRMS (ESI)**: calcd. for $C_{15}H_{13}F_3NO_2[M+H]^+$: 296.0893; found: 296.0891.

3-Methoxy-9-(2,2,2-trifluoroethyl)-8,9-dihydropyrido[1,2-*a*]indol-6(7*H*)-one (3k)

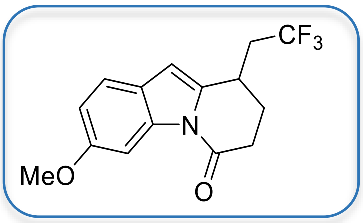

3k, white solid, 16.4 mg, 55%. **^{1}H NMR** (400 MHz, $CDCl_3$) δ 8.07 (d, *J* = 2.3 Hz, 1H), 7.35 (d, *J* = 8.5 Hz, 1H), 6.90 (dd, *J* = 8.5, 2.4 Hz, 1H), 6.31 (s, 1H), 3.87 (s, 3H), 3.33 (t, *J* = 9.8 Hz, 1H), 2.94 – 2.71 (m, 3H), 2.48 – 2.29 (m, 2H), 1.91 (qd, *J* = 10.8, 10.4, 3.8 Hz, 1H). **^{13}C NMR** (101 MHz, $CDCl_3$) δ 168.82, 158.19, 137.90, 136.14, 126.45 (q, *J* = 277.4 Hz), 122.78, 120.55, 113.34, 104.91, 100.83, 77.47, 77.16, 76.82, 55.81, 37.36 (q, *J* = 28.4 Hz), 33.03, 29.43, 29.40, 26.94. **^{19}F NMR** (376 MHz, $CDCl_3$) δ -63.22, -63.25, -63.28. **HRMS (ESI)**: calcd. for $C_{15}H_{15}F_3NO_2[M+H]^+$: 298.1049; found: 298.1051.

Methyl 6-oxo-9-(2,2,2-trifluoroethyl)-6,7,8,9-tetrahydropyrido[1,2-*a*]indole-3-carboxylate (3l)

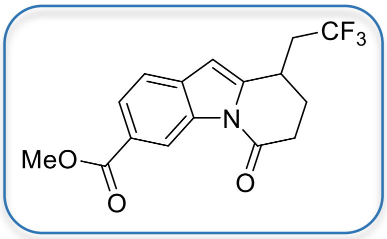

3l, white solid, 21.5 mg, yield 66%. **^{1}H NMR** (400 MHz, $CDCl_3$) δ 9.10 (s, 1H), 7.97 (d, *J* = 8.2 Hz, 1H), 7.51 (d, *J* = 8.2 Hz, 1H), 6.43 (s, 1H), 3.94 (s, 3H), 3.39 (dt, *J* = 9.9, 5.2 Hz, 1H), 2.98 – 2.80 (m, 3H), 2.50 – 2.35 (m, 2H), 2.05 – 1.90 (m, 1H). **^{13}C NMR** (101 MHz, $CDCl_3$) δ 168.29, 167.49, 142.48, 134.58, 132.88, 130.39, 126.58, 126.25 (q, *J* = 277.3 Hz), 125.56, 119.78, 118.05, 104.97, 77.48, 77.16, 76.84, 52.12, 37.19 (q, *J* = 28.6 Hz), 32.83, 29.51, 29.48, 29.46, 26.73. **^{19}F NMR** (376 MHz, $CDCl_3$) δ -63.25, -63.28, -63.31. **HRMS (ESI)**: calcd. for $C_{16}H_{15}F_3NO[M+H]^+$: 326.0999; found: 326.0992.

4-Bromo-9-(2,2,2-trifluoroethyl)-8,9-dihydropyrido[1,2-*a*]indol-6(7*H*)-one (3m)

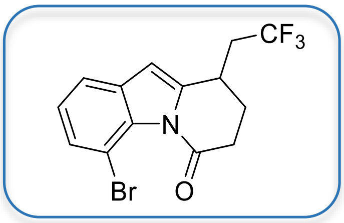

3m, white solid, 15.5 mg, yield 43%. **^{1}H NMR** (400 MHz, $CDCl_3$) δ 7.56 – 7.50 (m, 1H), 7.47 – 7.41 (m, 1H), 7.14 (td, J = 7.8, 2.2 Hz, 1H), 6.39 (s, 1H), 3.38 (s, 1H), 3.01 – 2.71 (m, 3H), 2.42 (tt, J = 18.5, 9.9 Hz, 2H), 2.01 (qd, J = 11.1, 4.9 Hz, 1H). **^{13}C NMR** (101 MHz, $CDCl_3$) δ 166.68, 142.59, 134.80, 133.61, 130.51, 126.31 (d, J = 277.4 Hz) 125.69, 122.17, 119.49, 109.50, 105.39, 77.47, 77.16, 76.84, 37.49 (q, J = 28.5 Hz), 33.55, 29.91, 29.88, 29.86, 29.83, 27.14. **^{19}F NMR** (376 MHz, $CDCl_3$) δ -63.27, -63.30, -63.33. **HRMS (ESI)**: calcd. for $C_{14}H_{12}BrF_3NO[M+H]^+$: 346.0049; found: 346.0053.

4-Methyl-9-(2,2,2-trifluoroethyl)-8,9-dihydropyrido[1,2-*a*]indol-6(7*H*)-one (3n)

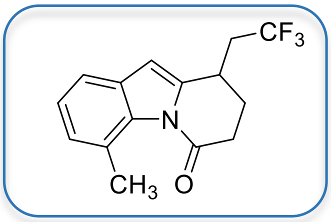

3n, white solid, 8.5mg, yield 30%. **^{1}H NMR** (400 MHz, $CDCl_3$) δ 7.32 (d, J = 7.7 Hz, 1H), 7.20 (t, J = 7.5 Hz, 1H), 7.13 (d, J = 7.4 Hz, 1H), 6.40 (d, J = 1.6 Hz, 1H), 3.36 (tt, J = 9.2, 4.4 Hz, 1H), 2.93 (dt, J = 17.4, 4.9 Hz, 1H), 2.81 (dddd, J = 14.5, 11.3, 5.7, 2.2 Hz, 2H), 2.62 (s, 3H), 2.48 – 2.33 (m, 2H), 1.97 (dtd, J = 13.2, 10.8, 4.7 Hz, 1H). **^{13}C NMR** (101 MHz, $CDCl_3$) δ 167.95, 140.98, 135.11, 131.00, 128.27, 127.08, 126.43 (q, J = 277.4 Hz), 124.78, 117.85, 106.01, 77.48, 77.16, 76.84, 37.56 (q, J = 28.3 Hz), 33.55, 29.93, 26.94, 23.05. **^{19}F NMR** (376 MHz, $CDCl_3$) δ -63.24, -63.27, -63.30. **HRMS (ESI)**: calcd. for $C_{15}H_{15}F_3NO[M+H]^+$: 282.1100; found: 268.0099.

7-Methyl-9-(2,2,2-trifluoroethyl)-8,9-dihydropyrido[1,2-*a*]indol-6(7*H*)-one (3o)

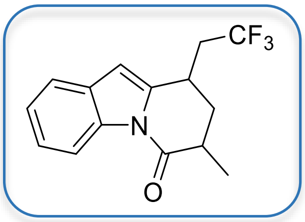

3o, white solid, 12.1 mg, yield 43%. ^{1}**H NMR** (400 MHz, $CDCl_3$) δ 8.52 (d, *J* = 8.1 Hz, 1H), 7.53 (dd, *J* = 7.4, 1.4 Hz, 1H), 7.36 (td, *J* = 7.7, 1.5 Hz, 1H), 7.33 – 7.28 (m, 1H), 6.41 (d, *J* = 1.9 Hz, 1H), 3.49 – 3.35 (m, 1H), 3.03 – 2.79 (m, 2H), 2.52 – 2.34 (m, 2H), 1.77 (q, *J* = 12.9 Hz, 1H), 1.47 (d, *J* = 6.9 Hz, 3H). ^{13}**C NMR** (101 MHz, $CDCl_3$) δ 172.16, 140.05, 135.41, 129.48, 125.23, 124.98, 124.26, 120.23, 116.66, 104.54, 77.48, 77.16, 76.84,38.68, 37.57 (q, *J* = 28.6, 26.2 Hz), 36.05, 30.23, 15.98. ^{19}**F NMR** (376 MHz, $CDCl_3$) δ -63.03, -63.06, -63.09. **HRMS (ESI)**: calcd. for $C_{15}H_{15}F_3NO[M+H]^+$: 282.1100; found:282.1088.

10-(2,2,2-Trifluoroethyl)-7,8,9,10-tetrahydro-6*H*-azepino[1,2-*a*]indol-6-one (3p)

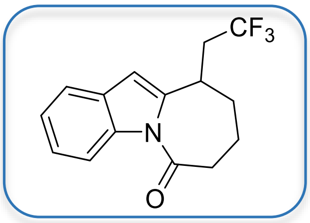

3p, white solid, 14.6mg, yield 52%. ^{1}**H NMR** (400 MHz, $CDCl_3$) δ 8.40 (dd, *J* = 8.3, 1.0 Hz, 1H), 7.57 – 7.50 (m, 1H), 7.41 – 7.31 (m, 2H), 7.30 – 7.27 (m, 1H), 6.42 – 6.39 (m, 1H), 3.52 (tt, *J* = 9.9, 5.1 Hz, 1H), 3.11 – 2.81 (m, 3H), 2.62 (dqd, *J* = 14.8, 10.2, 9.0 Hz, 1H), 2.37 – 2.22 (m, 1H), 2.07 (dddd, *J* = 12.1, 8.5, 6.2, 2.4 Hz, 1H), 1.88 – 1.68 (m, 2H). ^{13}**C NMR** (101 MHz, $CDCl_3$) δ 173.82, 140.06, 136.85, 128.88,127.84, 126.46 (d, *J* = 277.2 Hz), 125.08, 123.75, 120.01, 115.96, 106.33, 77.48, 77.16, 76.84, 37.80 (q, *J* = 28.6 Hz), 36.24, 31.18, 30.45, 20.18. ^{19}**F NMR** (376 MHz, $CDCl_3$) δ -62.98, -63.01, -63.04. **HRMS (ESI)**: calcd. for $C_{15}H_{15}F_3NO[M+H]^+$: 282.1100; found: 282.1100.

9-(2,2,2-Trifluoroethyl)-6,7,8,9-tetrahydropyrido[1,2-*a*]indole (3q)

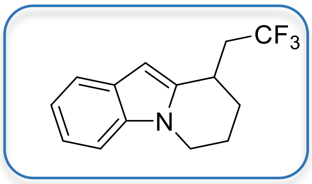

3q, white solid, 12mg, 25%. Its NMR data was the same with pre-reported one[7]. ^{1}**H NMR** (400 MHz, $CDCl_3$) δ 7.59 (dt, *J* = 7.7, 1.0 Hz, 1H), 7.31 (d, *J* = 8.2 Hz, 1H), 7.21 (ddd, *J* = 8.2, 7.0, 1.3 Hz, 1H), 7.14 (ddd, *J* = 8.0, 7.0, 1.2 Hz, 1H), 6.33 (s, 1H), 4.25 (ddd, *J* = 11.7, 5.7, 3.5 Hz, 1H), 4.00 – 3.87 (m, 1H), 3.41 (tt, *J* = 9.8, 4.4 Hz, 1H), 2.88 (dqd, *J* = 15.4, 11.8, 3.7 Hz, 1H), 2.49 – 2.21 (m, 3H), 2.15 – 2.01 (m, 1H). ^{13}**C NMR**

(101 MHz, $CDCl_3$) δ 138.51, 136.47, 127.87, 126.64 (d, J = 247.6 Hz),122.48, 121.10, 120.07, 120.04, 119.88, 108.93, 97.53, 77.47, 77.16, 76.84, 42.18, 39.10 (q, J = 27.5, 26.8 Hz), 29.95, 27.22, 24.47, 22.05, 1.09, 0.06. **^{19}F NMR** (376 MHz, $CDCl_3$) δ -63.28, -63.31, -63.34. **HRMS (ESI)**: calcd. for $C_{14}H_{15}F_3N[M+H]^+$: 254.1151; found: 254.1148.

8-(2,2,2-Trifluoroethyl)-7,8-dihydroindolizin-5(6*H*)-one (3r)

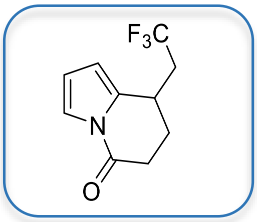

3r, white solid, 11 mg, yield 20%. **^{1}H NMR** (400 MHz, $CDCl_3$) δ 7.45 – 7.41 (m, 1H), 6.29 (t, J = 3.3 Hz, 1H), 6.09 (dt, J = 3.3, 1.7 Hz, 1H), 3.26 (dq, J = 9.6, 4.9, 4.2 Hz, 1H), 2.91 – 2.67 (m, 3H), 2.36 (ddt, J = 20.4, 15.3, 7.6 Hz, 2H), 1.92 (dtd, J = 13.3, 11.2, 4.3 Hz, 1H). **^{13}C NMR** (101 MHz, $CDCl_3$) δ 167.62, 134.27, 117.12, 112.63, 109.06, 77.48, 77.16, 76.84, 37.55 (q, J = 28.0 Hz), 32.21, 28.83, 27.64. **^{19}F NMR** (376 MHz, $CDCl_3$) δ -63.33, -63.36, -63.39. **HRMS (ESI)**: calcd. for $C_{10}H_{11}F_3NO[M+H]^+$: 218.0787; found: 218.0786.

1-Methyl-1-(2,2,2-trifluoroethyl)-2,3-dihydro-1*H*-pyrrolo[1,2-*a*]indole (3s)

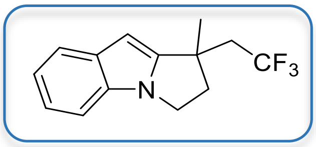

3s, white solid, 7.3mg, yield 29%. Its NMR data was consistent with a previous report[8]. **^{1}H NMR** (400 MHz, $CDCl_3$) δ 7.61 (d, J = 7.8 Hz, 1H), 7.32 – 7.27 (m, 1H), 7.20 (ddd, J = 8.1, 7.0, 1.3 Hz, 1H), 7.12 (t, J = 7.5 Hz, 1H), 6.21 (s, 1H), 4.24 – 4.10 (m, 2H), 2.81 – 2.64 (m, 2H), 2.64 – 2.48 (m, 2H). **^{13}C NMR** (101 MHz, $CDCl_3$) δ 150.43, 132.69, 132.34, 120.98, 120.90, 119.51, 109.57, 91.31, 77.48, 77.16, 76.84, 43.38 (q, J = 27.0 Hz), 42.36, 41.99, 38.85, 25.63, 1.10. **^{19}F NMR** (376 MHz, $CDCl_3$) δ -60.50, -60.53, -60.56. **HRMS (ESI)**: calcd. for $C_{14}H_{15}F_3N[M+H]^+$: 254.1151; found: 254.1154.

5-Methyl-5-(2,2,2-trifluoroethyl)-5,6-dihydroindolo[2,1-*a*]isoquinoline (3t)

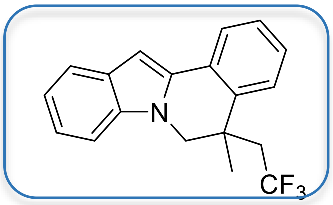

3t, white solid, 10.5 mg, yield 29%. **^{1}H NMR** (400 MHz, $CDCl_3$) δ 7.48 (dt, *J* = 7.6, 2.3 Hz, 1H), 7.45 – 7.35 (m, 3H), 7.31 – 7.25 (m, 1H), 7.18 (td, *J* = 7.5, 3.1 Hz, 1H), 6.96 (d, *J* = 3.1 Hz, 1H), 4.48 (dd, *J* = 12.7, 3.1 Hz, 1H), 3.89 (dd, *J* = 12.6, 3.1 Hz, 1H), 2.45 – 2.20 (m, 2H). **^{13}C NMR** (101 MHz, $CDCl_3$) δ 138.47, 136.90, 134.72, 128.79, 128.09, 128.00, 127.89, 127.77, 125.08, 125.00, 122.17, 120.92, 120.24, 109.04, 97.25, 77.41, 77.09, 76.78, 49.69, 41.12 (q, *J* = 26.5 Hz), 36.57, 22.30, 8.85. **^{19}F NMR** (376 MHz, $CDCl_3$) δ -59.53, -59.56, -59.59. **HRMS (ESI)**: calcd. for $C_{19}H_{17}F_3N[M+H]^+$: 316.1308; found: 316.1316.

***N*-(2-(2-Methoxy-6-oxo-9-(2,2,2-trifluoroethyl)-6,7,8,9-tetrahydropyrido[1,2-*a*]indol-10-yl)ethyl)acetamide (3u)**

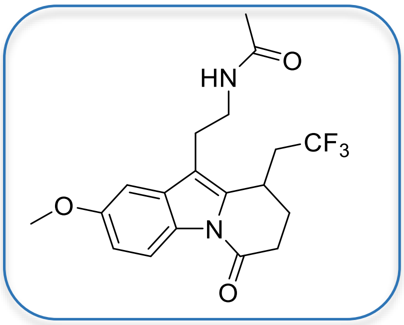

3u, light green oil, 29 mg, yield 75%. **^{1}H NMR** (400 MHz, $CDCl_3$) δ 8.27 (d, *J* = 8.9 Hz, 1H), 7.02 (d, *J* = 2.1 Hz, 1H), 6.88 (t, *J* = 8.4 Hz, 1H), 6.21 – 5.88 (m, 1H), 3.82 (d, *J* = 4.8 Hz, 3H), 3.68 – 3.56 (m, 1H), 3.47 (dq, *J* = 19.8, 6.6 Hz, 2H), 2.86 – 2.67 (m, 4H), 2.57 – 2.28 (m, 2H), 2.25 – 2.10 (m, 2H), 1.91 (s, 3H),1.63(s,2H). **^{13}C NMR** (101 MHz, $CDCl_3$) δ 171.27, 170.43, 170.33, 167.75, 157.05, 155.92, 149.70, 136.14, 131.29, 130.88, 129.36, 126.09 (d, *J* = 277.9 Hz), 123.76, 117.57, 117.46, 116.09, 114.76, 113.11, 112.91, 102.00, 101.74, 83.55, 77.48, 77.16, 76.84, 60.45, 55.79, 55.76, 39.16, 36.56 (q, *J* = 27.7 Hz), 29.03, 28.24, 25.40, 25.14, 24.52, 24.34, 23.30, 23.17, 21.06, 14.21. **^{19}F NMR** (376 MHz, $CDCl_3$) δ -63.97, -64.00, -64.02. **HRMS (ESI)**: calcd. for $C_{19}H_{22}F_3N_2O_3[M+H]^+$: 383.1577; found: 383.1577.

2-(6-Oxo-9-(2,2,2-trifluoroethyl)-6,7,8,9-tetrahydropyrido[1,2-*a*]indol-10-yl)acetonitrile (3v)

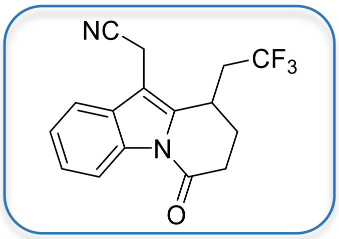

3v, white solid, 21mg, yield 68%. **^{1}H NMR** (400 MHz, $CDCl_3$) δ 8.46 (d, J = 8.5 Hz, 1H), 7.57 (d, J = 7.6 Hz, 1H), 7.37 (t, J = 7.9 Hz, 2H), 3.72 (s, 3H), 2.85 (s, 2H), 2.56 – 2.44 (m, 2H), 2.25 (s, 2H). **^{13}C NMR** (101 MHz, $CDCl_3$) δ 167.92, 136.14, 134.63, 126.04 (d, J = 277.8 Hz), 118.04, 116.88, 116.57, 106.82, 77.47, 77.16, 76.84, 36.49 (q, J = 27.8 Hz), 29.75, 29.17, 25.33, 25.06, 21.10, 12.90. **^{19}F NMR** (376 MHz, $CDCl_3$) δ -64.07. **HRMS (ESI)**: calcd. for $C_{16}H_{14}F_3N_2O[M+H]^+$: 307.1053; found: 307.1052.

3. NMR spectra of products

3a ^{1}H NMR (400 MHz, $CDCl_3$)

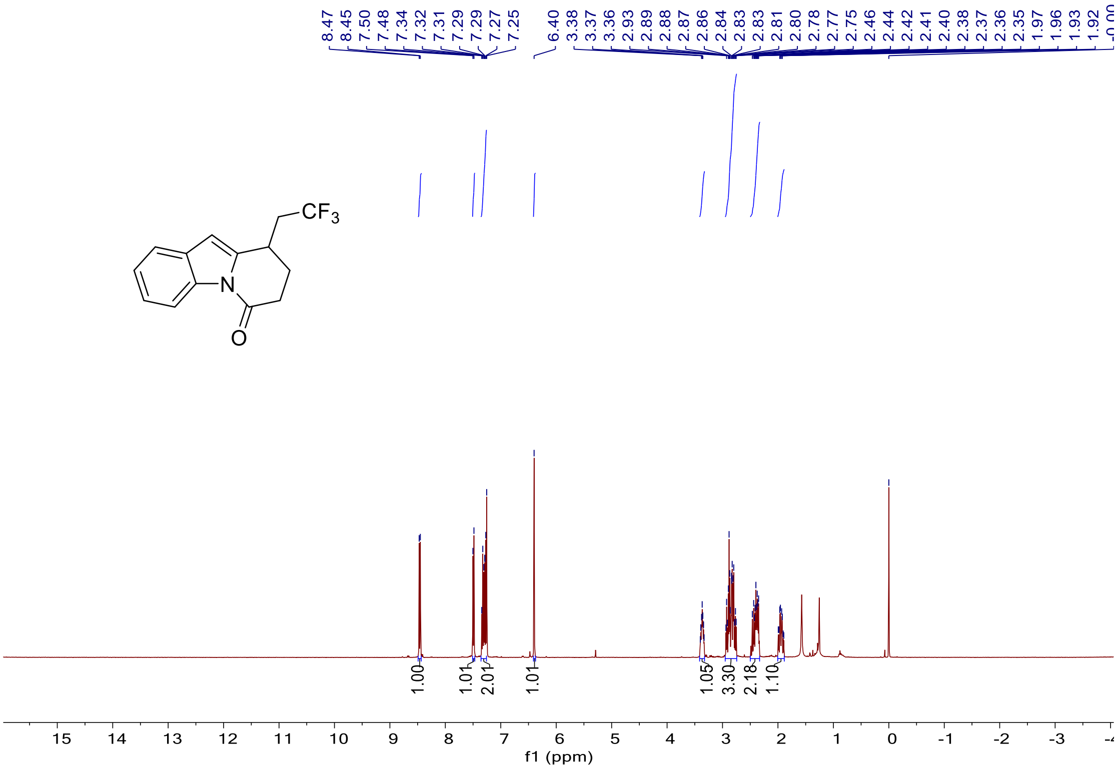

^{13}C NMR (101 MHz, $CDCl_3$)

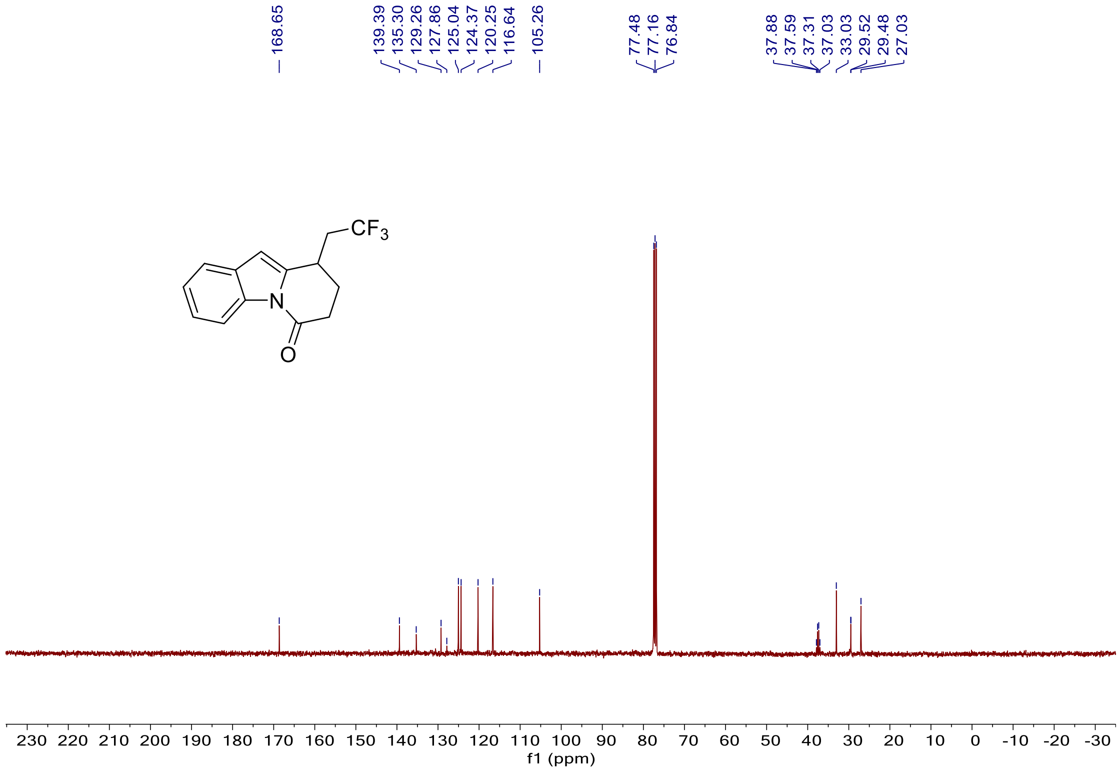

^{19}F NMR (376 MHz, $CDCl_3$)

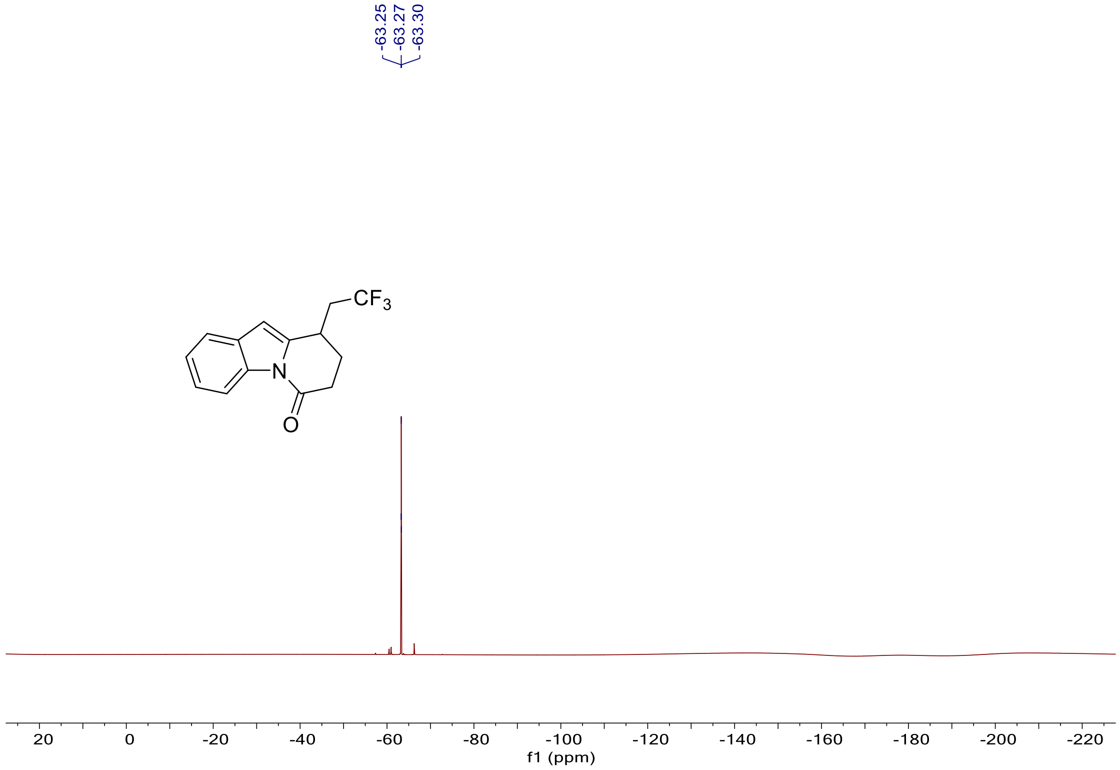

3b ^{1}H NMR (400 MHz, $CDCl_3$)

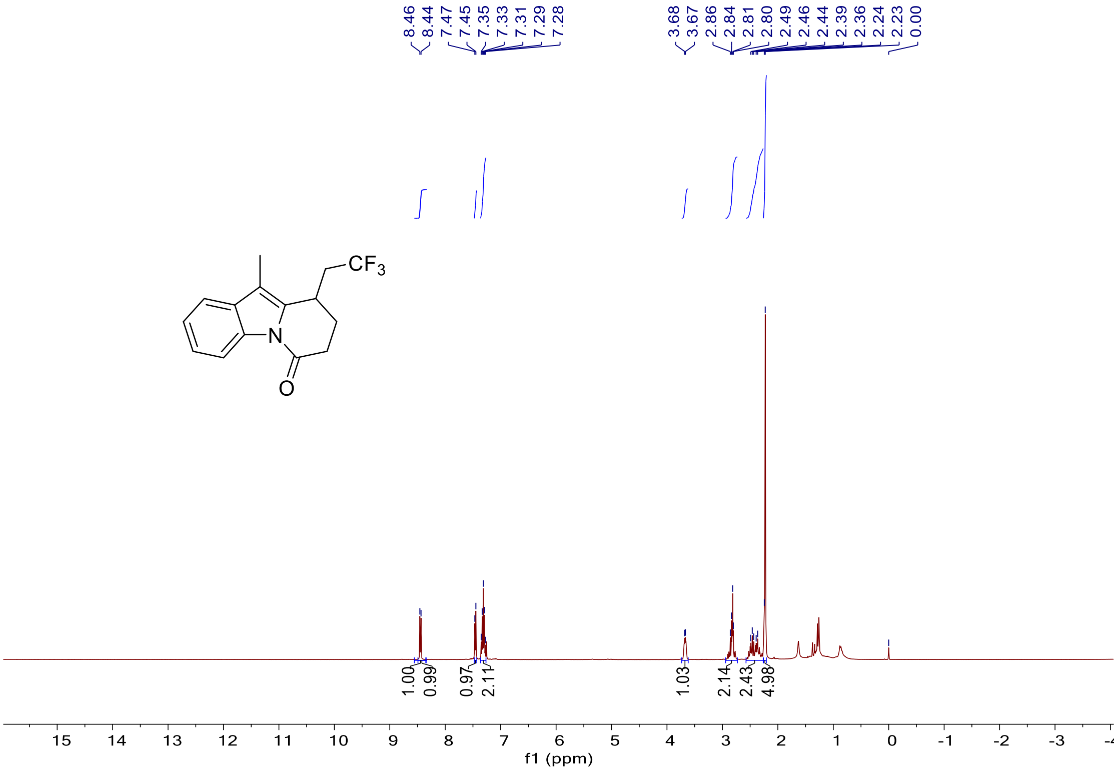

^{13}C NMR (101 MHz, $CDCl_3$)

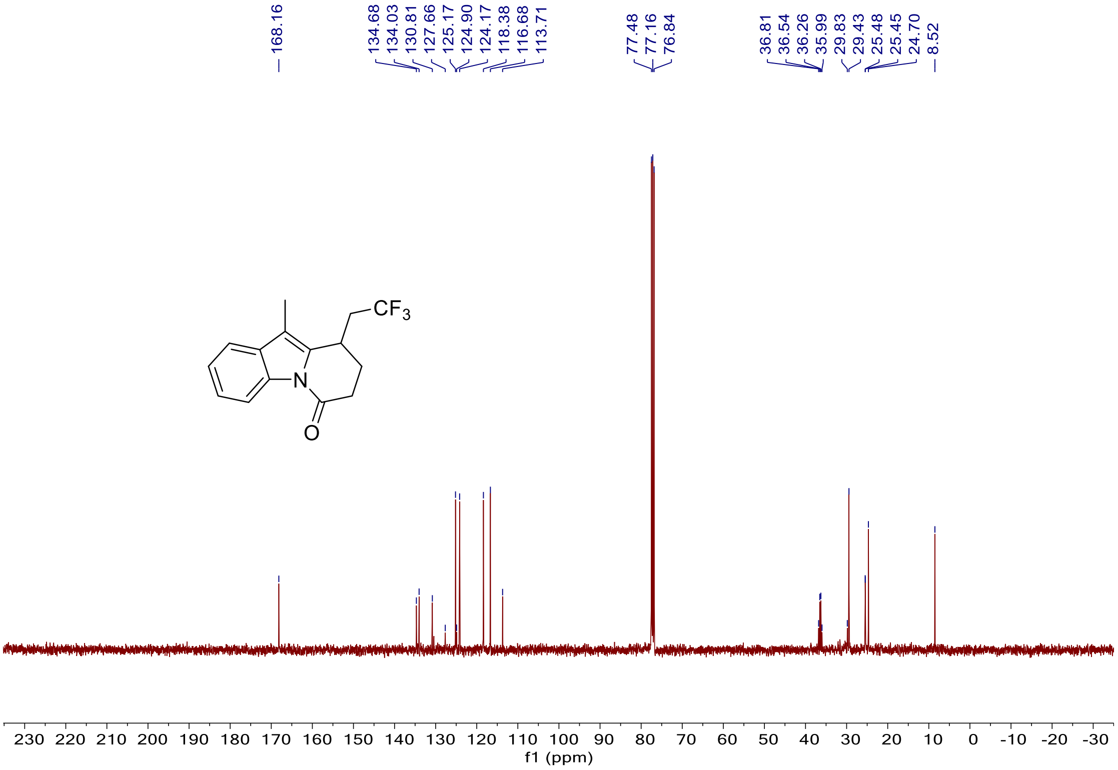

^{19}F NMR (376 MHz, $CDCl_3$)

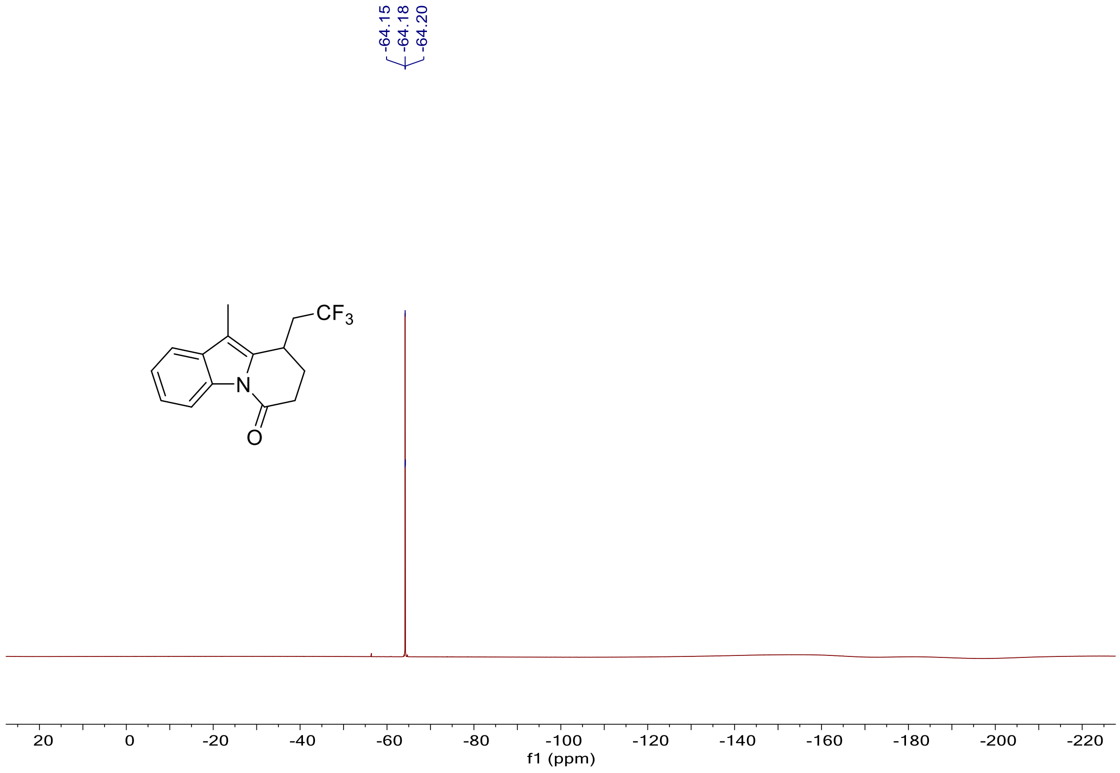

3c ^{1}H NMR (400 MHz, $CDCl_3$)

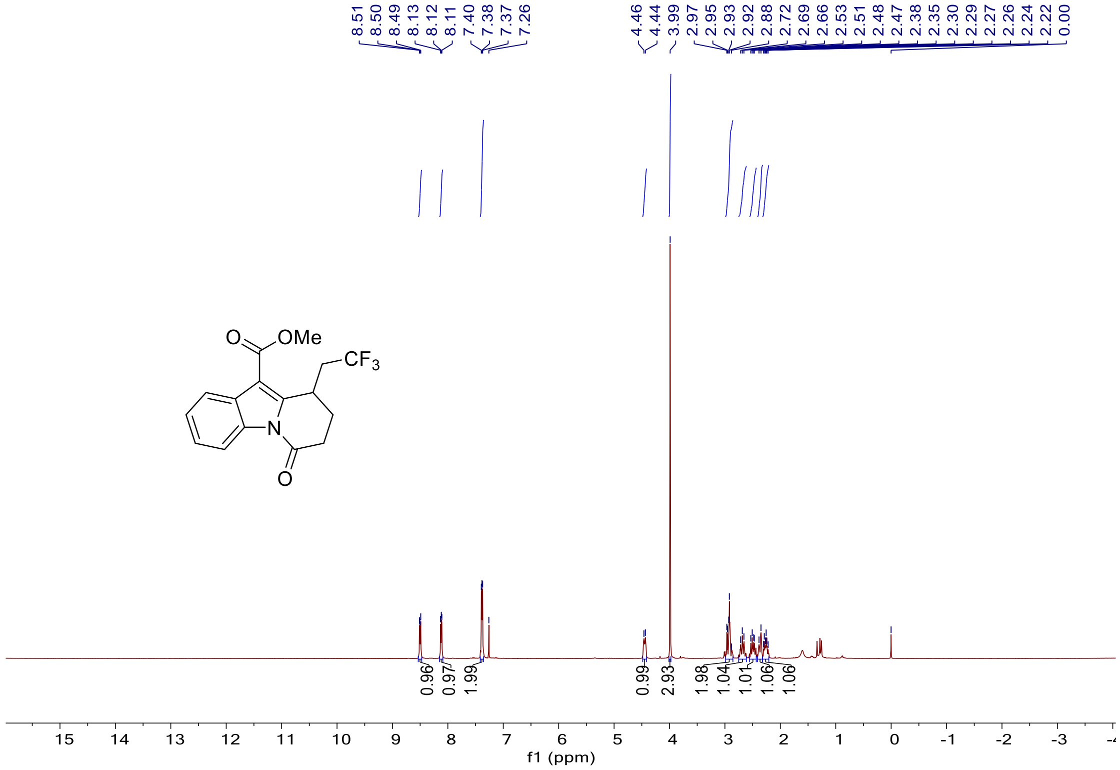

^{13}C NMR (101 MHz, $CDCl_3$)

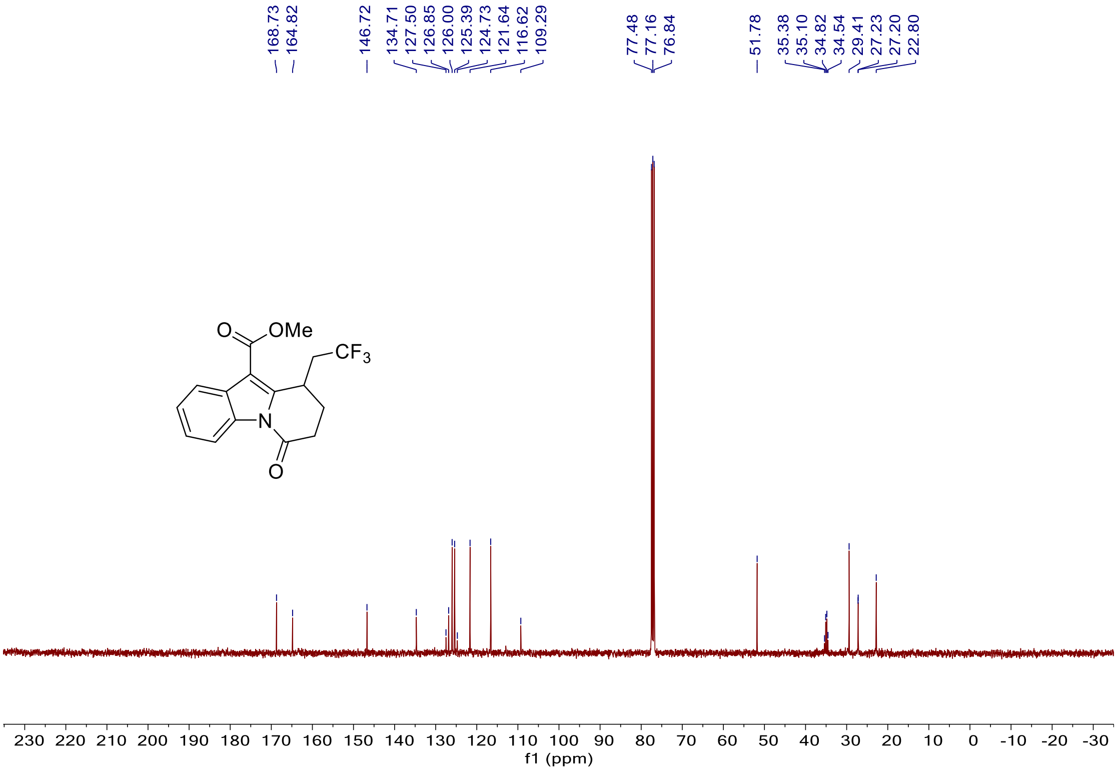

^{19}F NMR (376 MHz, $CDCl_3$)

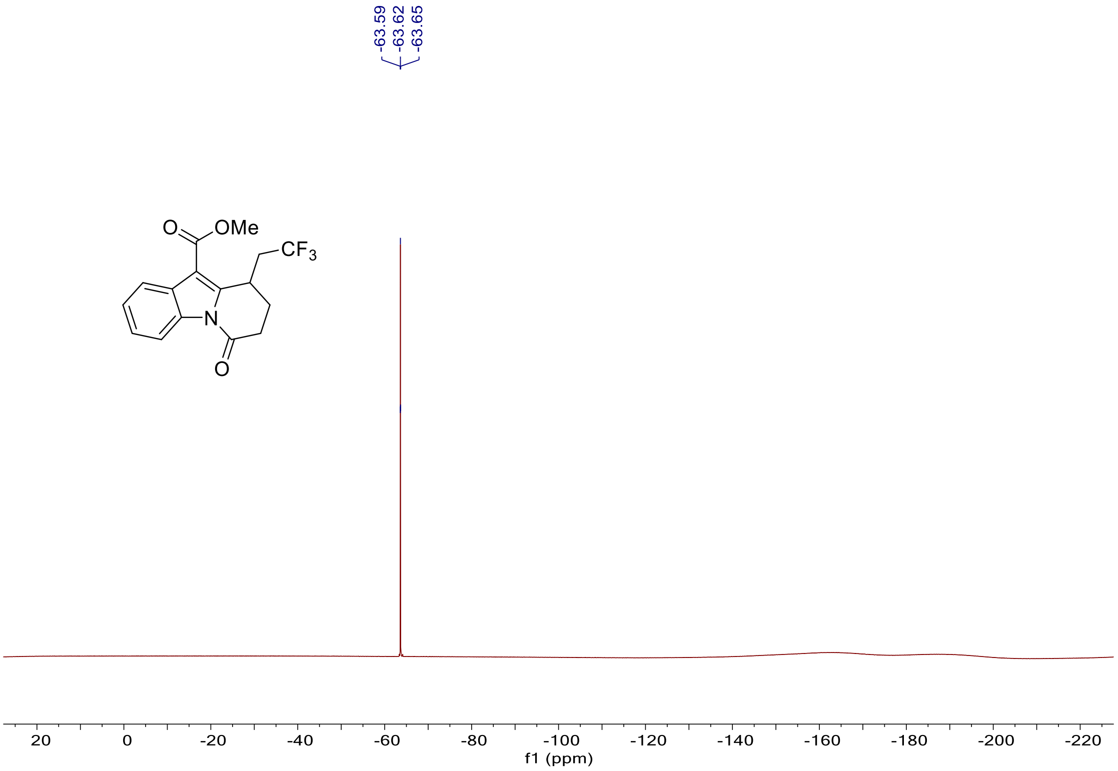

3d ^{1}H NMR (400 MHz, $CDCl_3$)

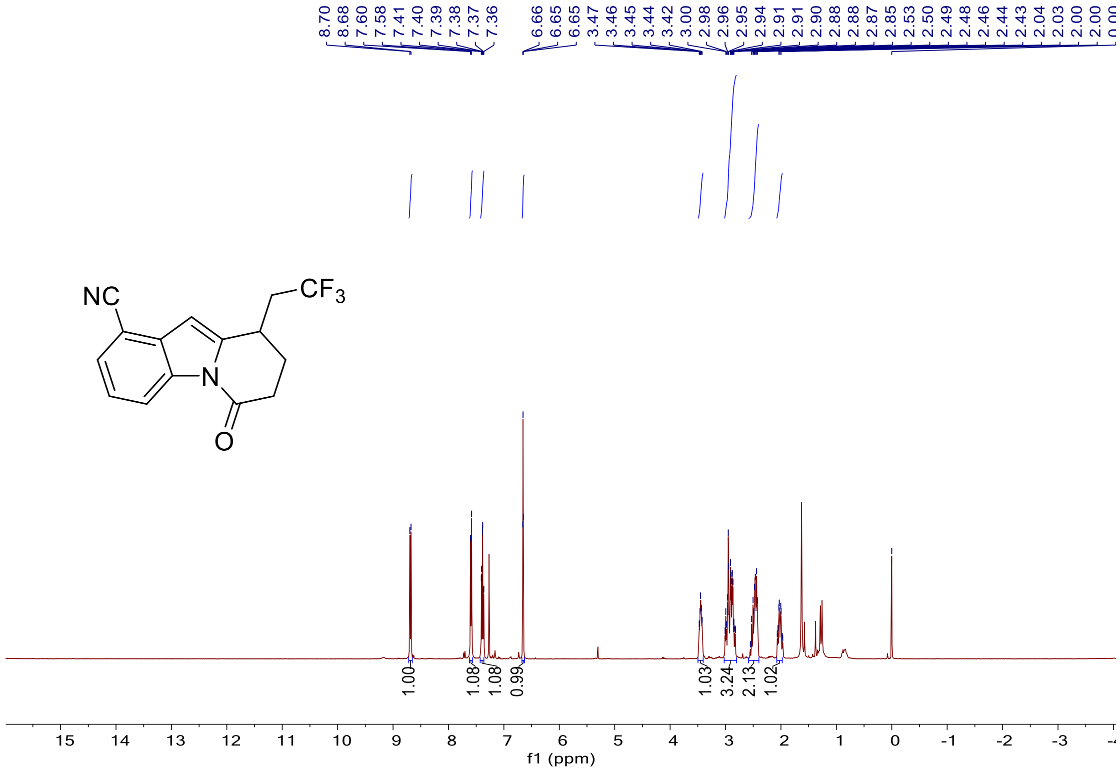

^{13}C NMR (101 MHz, $CDCl_3$)

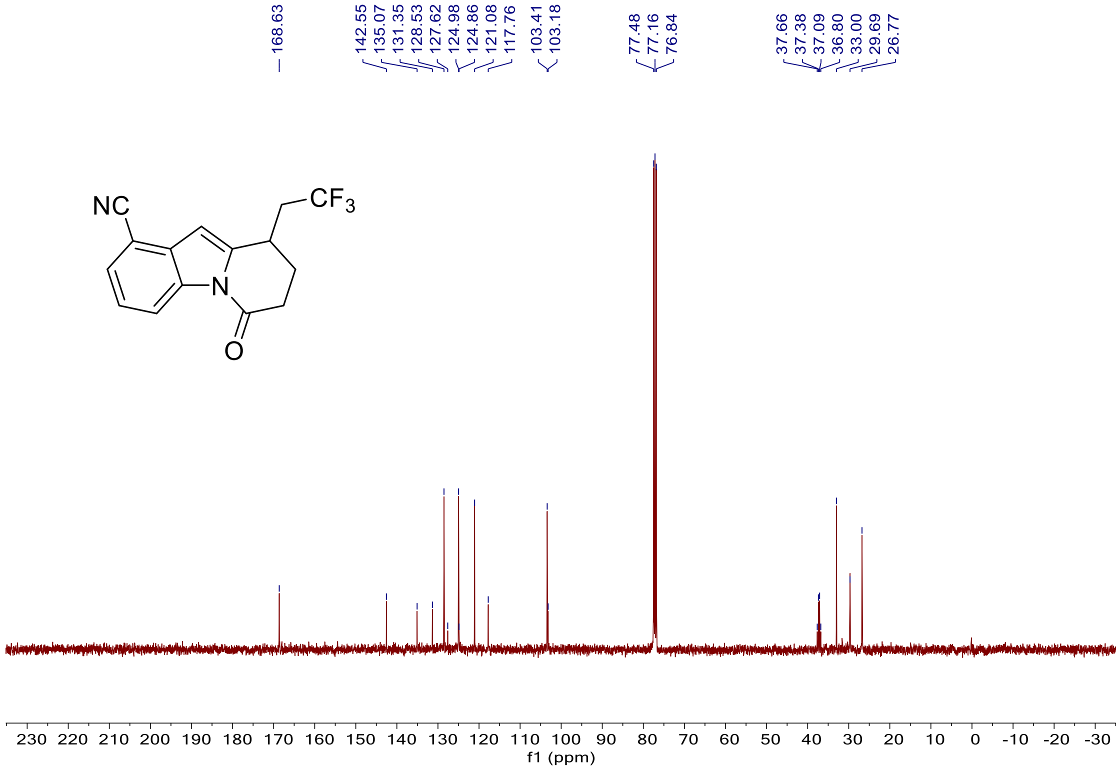

^{19}F NMR (376 MHz, $CDCl_3$)

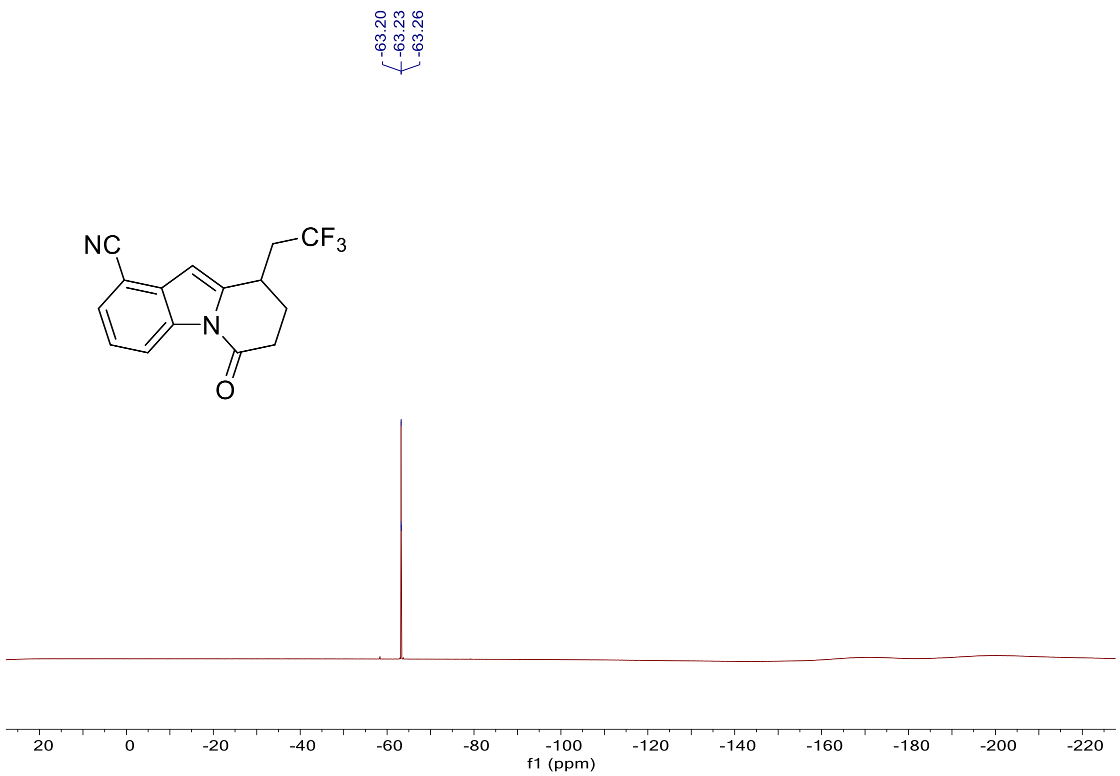

3e ^{1}H NMR (400 MHz, $CDCl_3$)

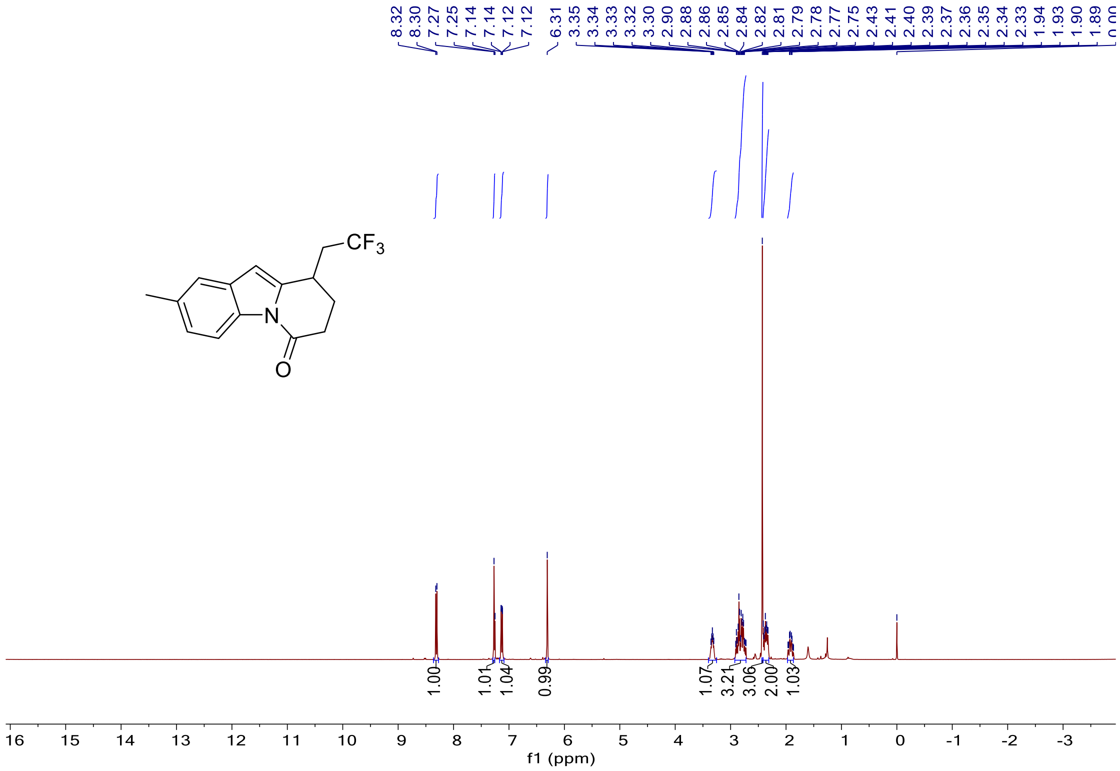

^{13}C NMR (101 MHz, $CDCl_3$)

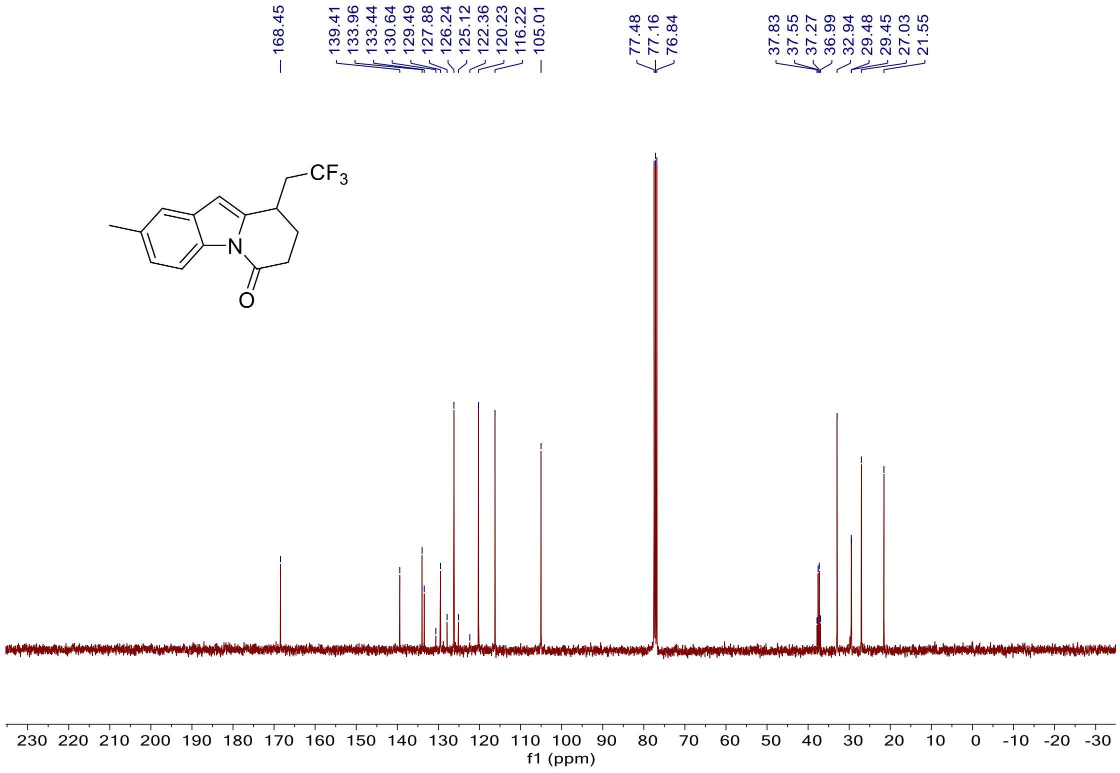

^{19}F NMR (376 MHz, $CDCl_3$)

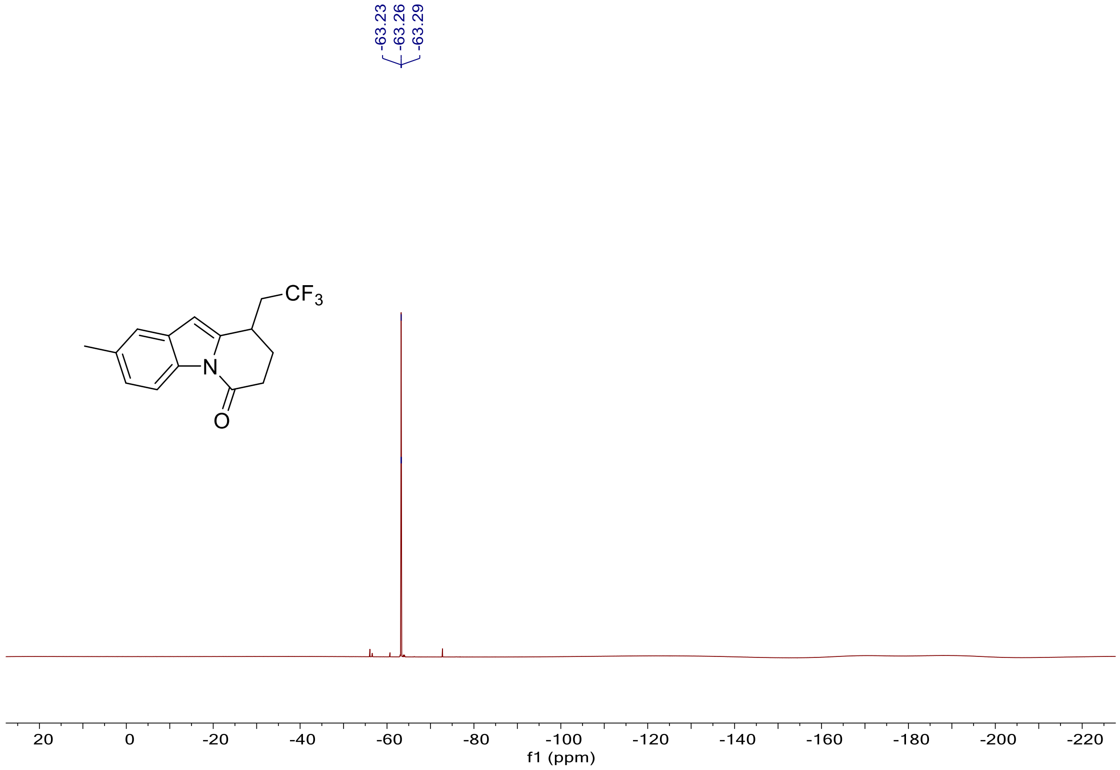

3f ^{1}H NMR (400 MHz, $CDCl_3$)

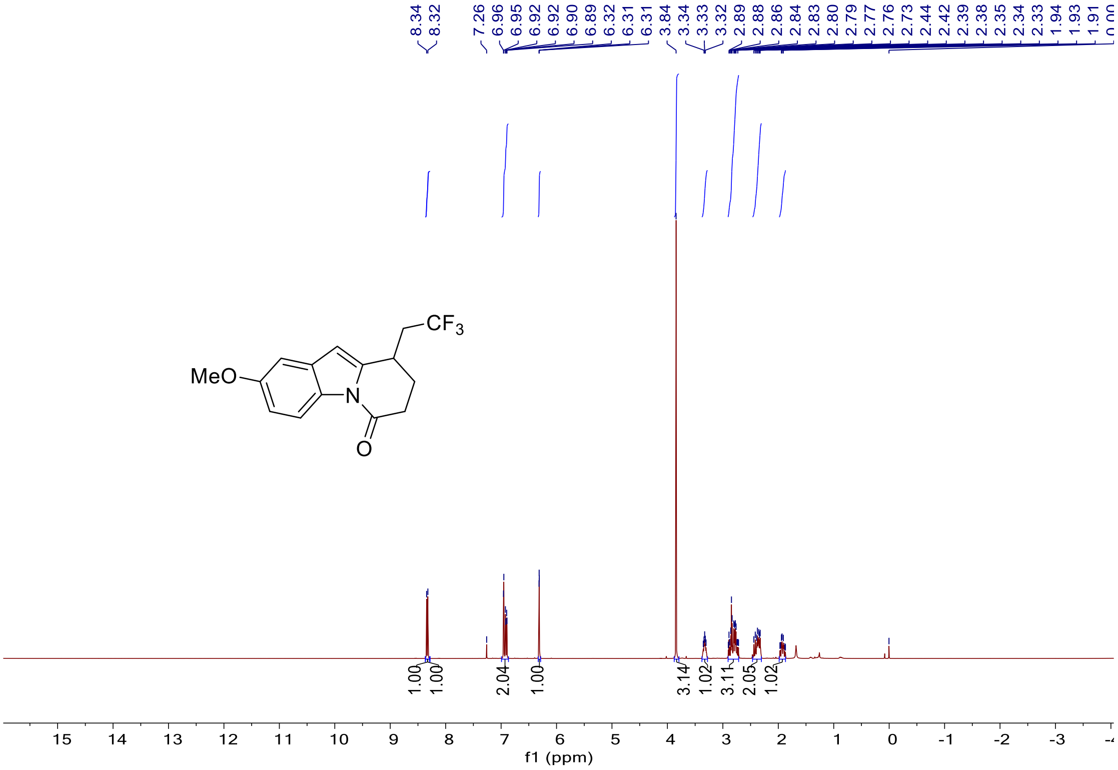

^{13}C NMR (101 MHz, $CDCl_3$)

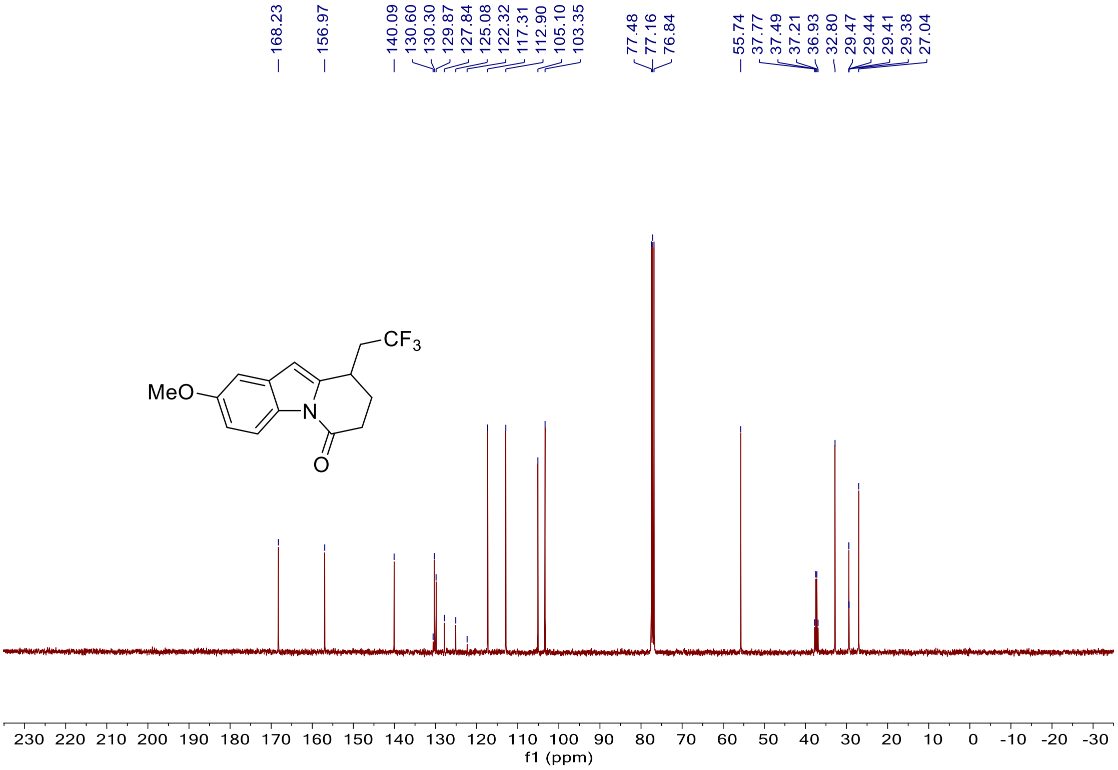

^{19}F NMR (376 MHz, $CDCl_3$)

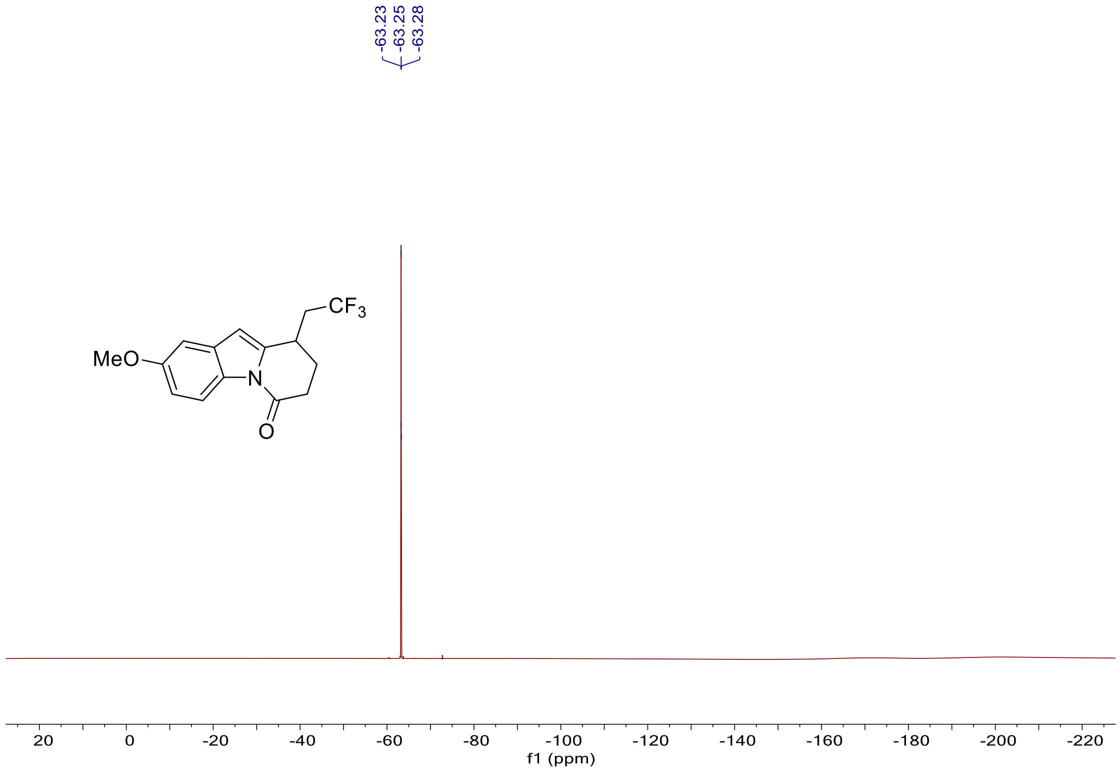

3g ^{1}H NMR (400 MHz, $CDCl_3$)

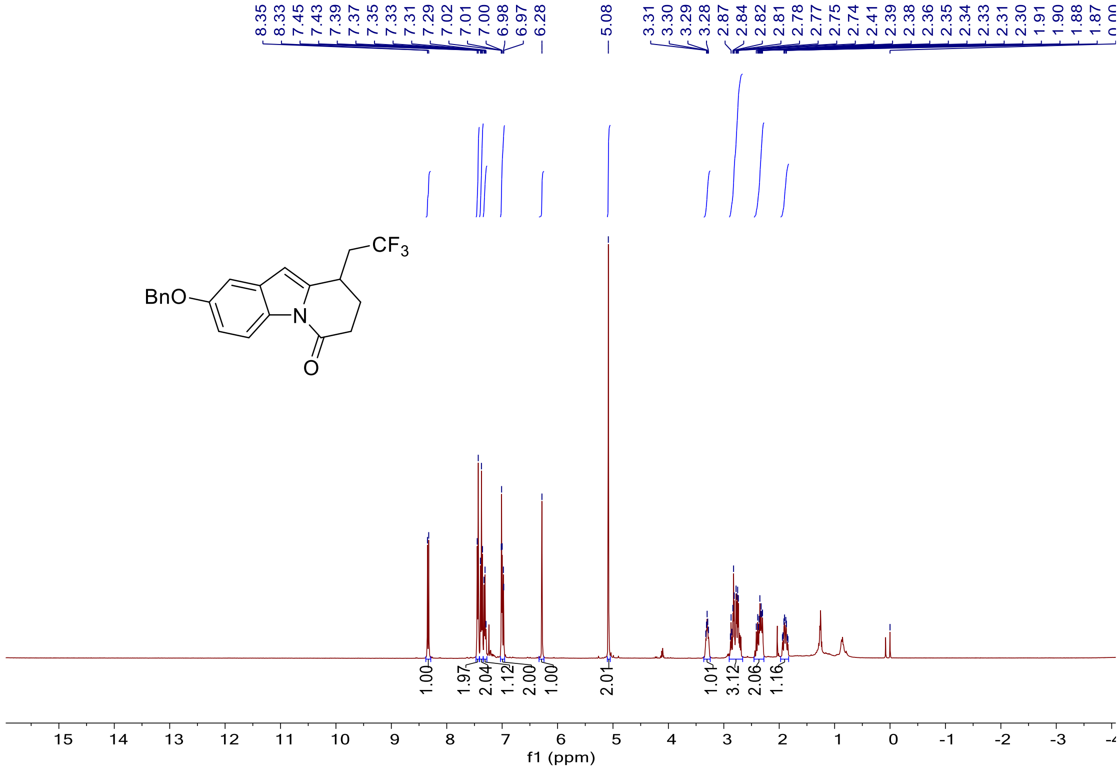

^{13}C NMR (101 MHz, $CDCl_3$)

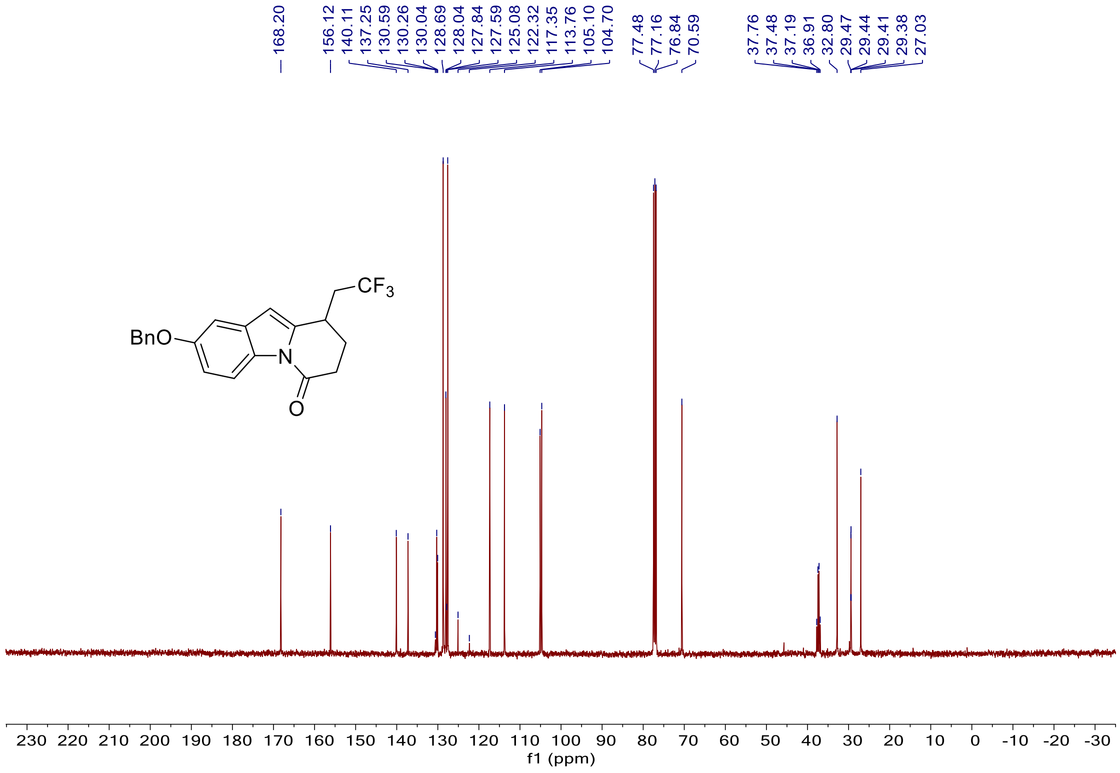

^{19}F NMR (376 MHz, $CDCl_3$)

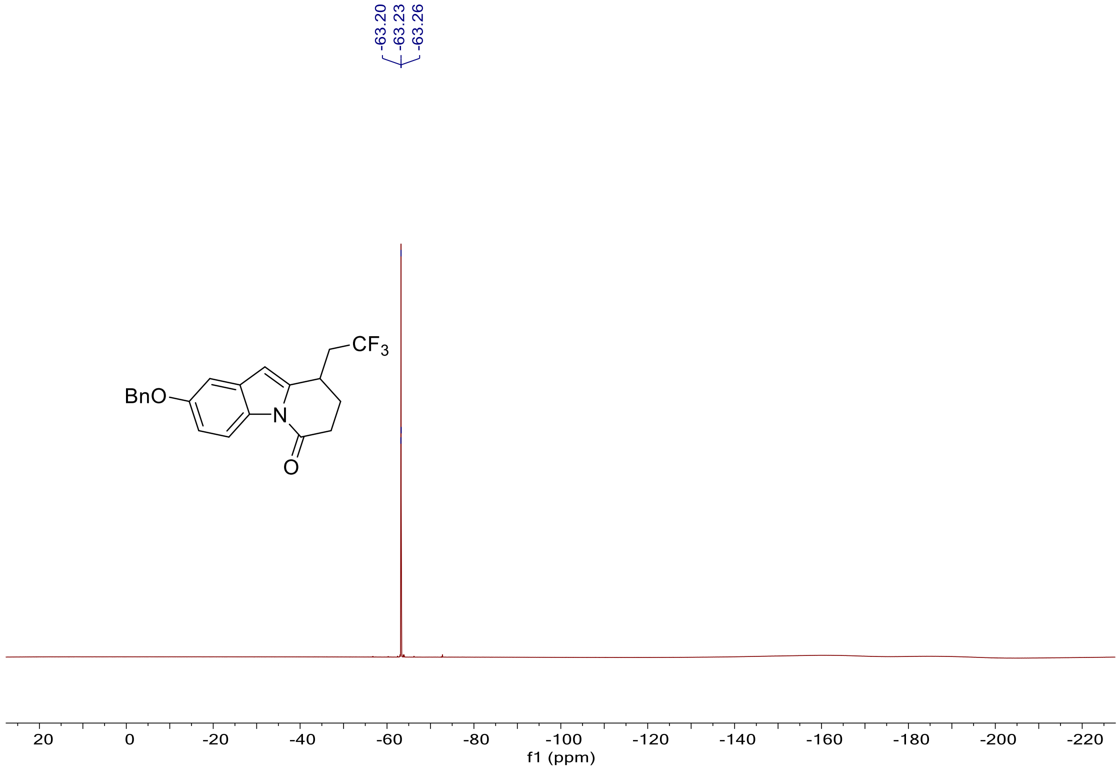

3h ^{1}H NMR (400 MHz, $CDCl_3$)

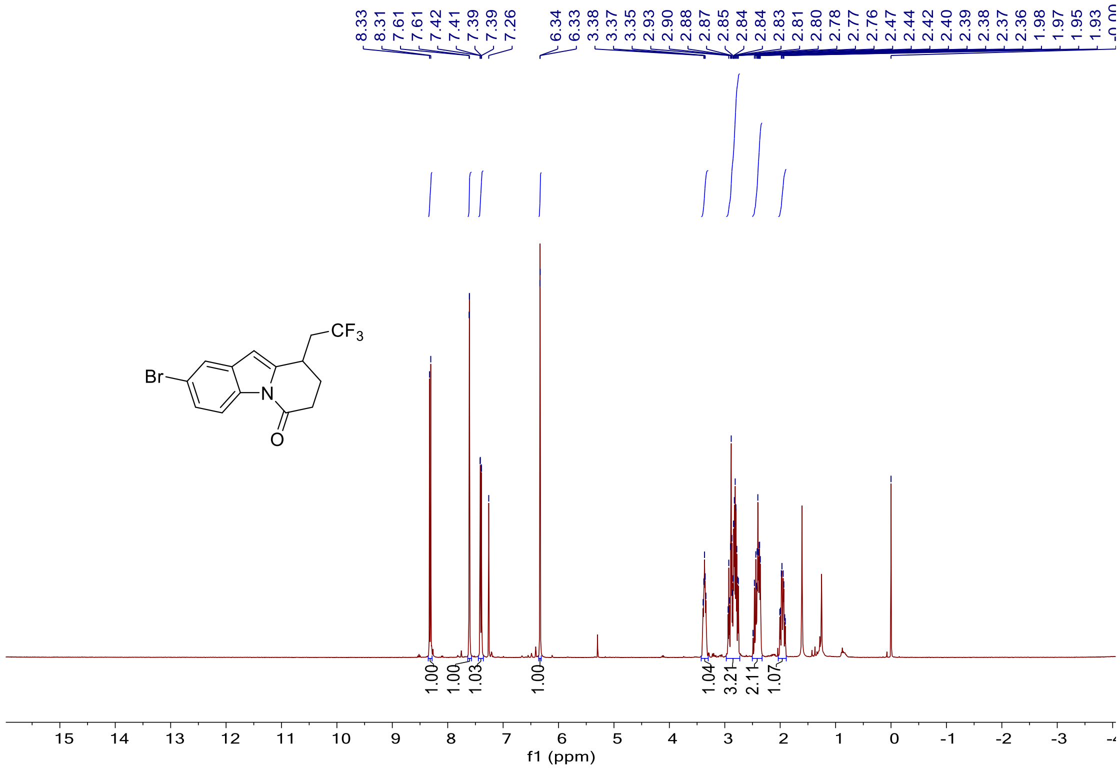

^{13}C NMR (101 MHz, $CDCl_3$)

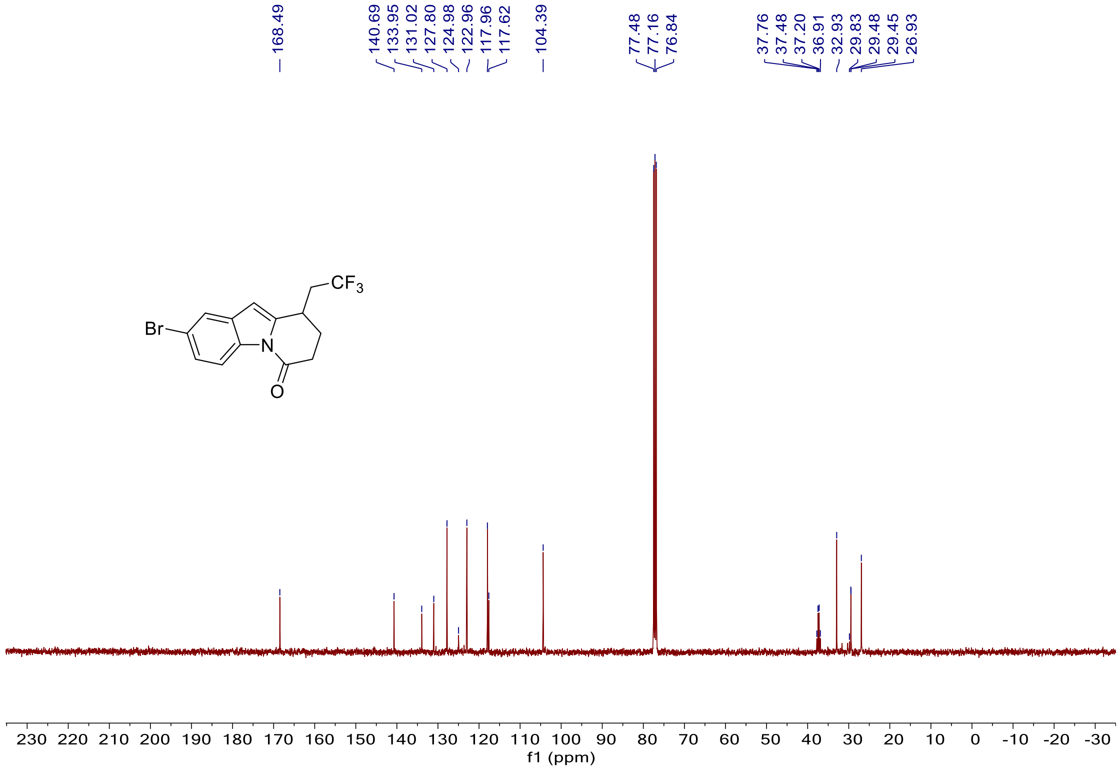

^{19}F NMR (376 MHz, $CDCl_3$)

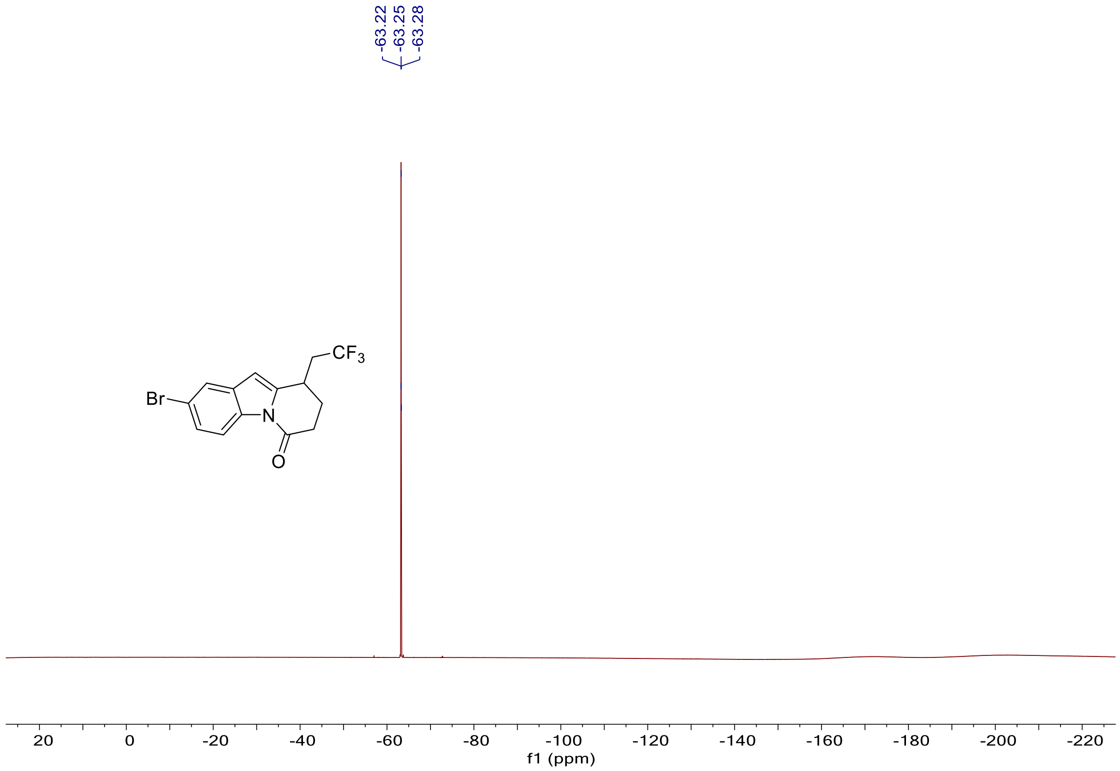

3i ^{1}H NMR (400 MHz, $CDCl_3$)

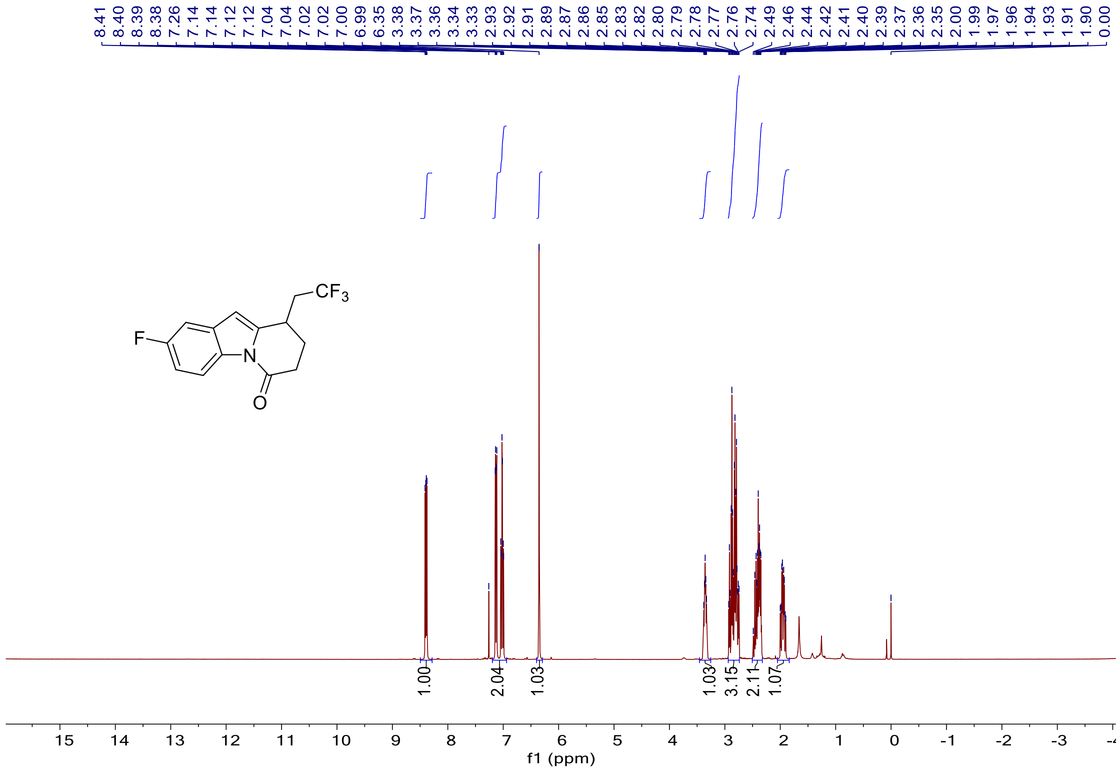

^{13}C NMR (101 MHz, $CDCl_3$)

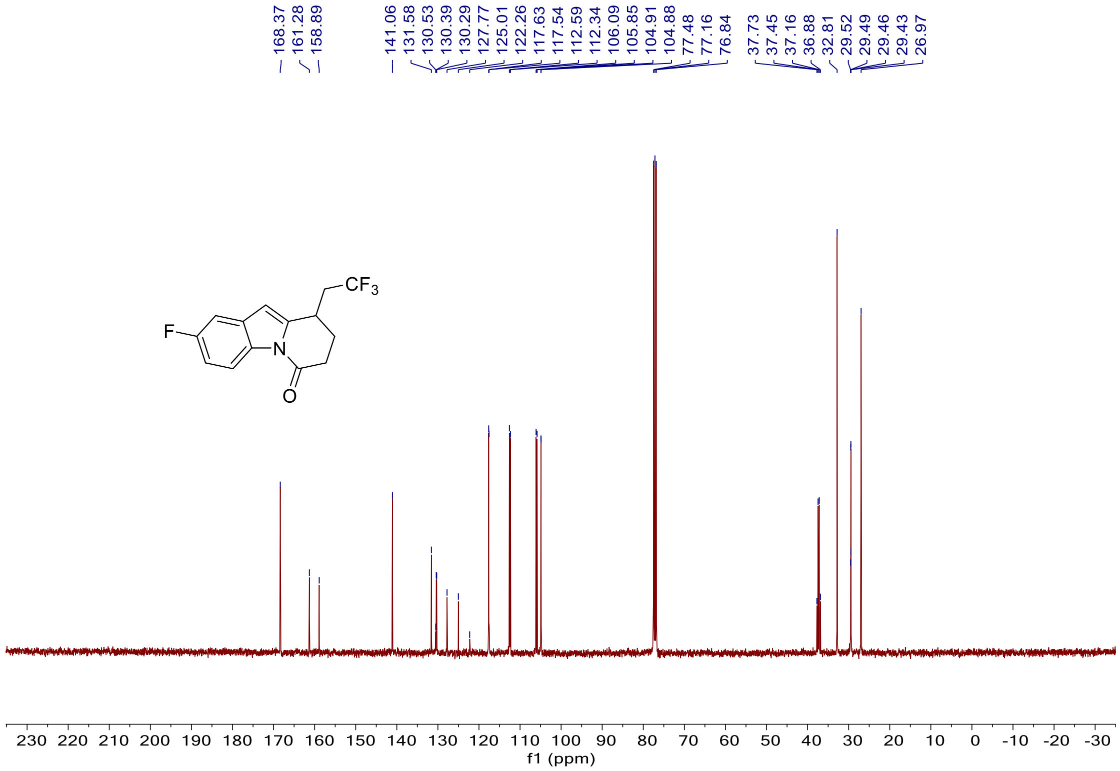

^{19}F NMR (376 MHz, $CDCl_3$)

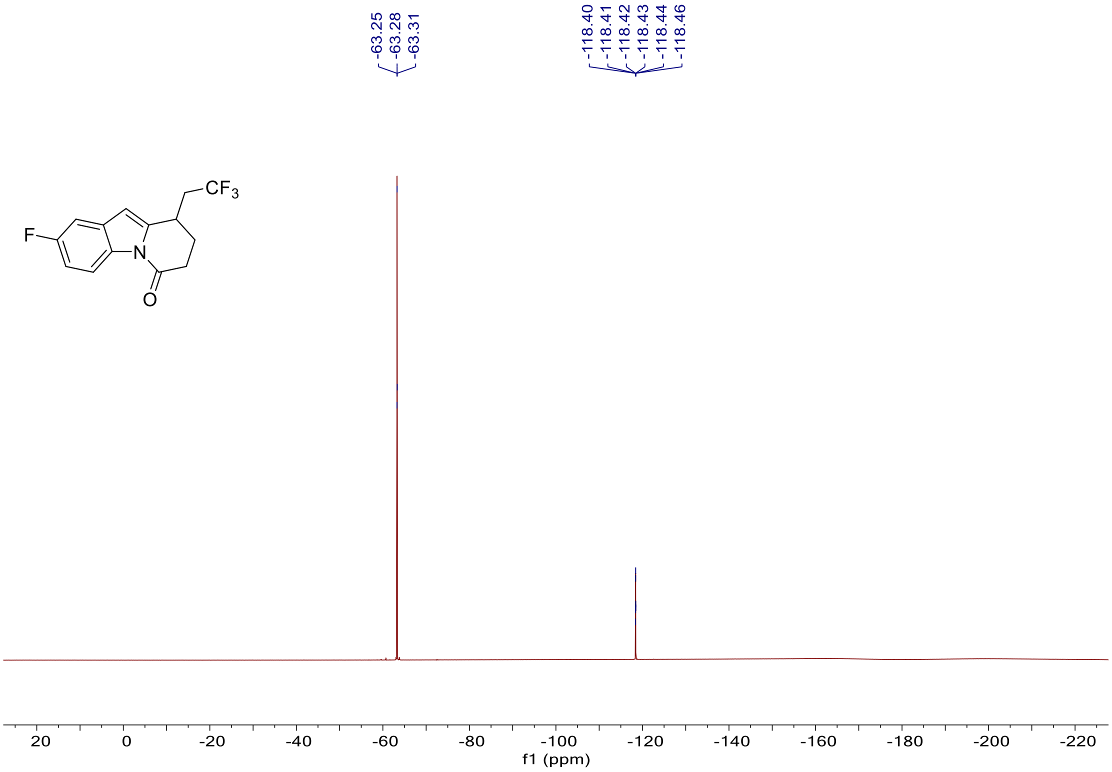

3j ^{1}H NMR (400 MHz, $CDCl_3$)

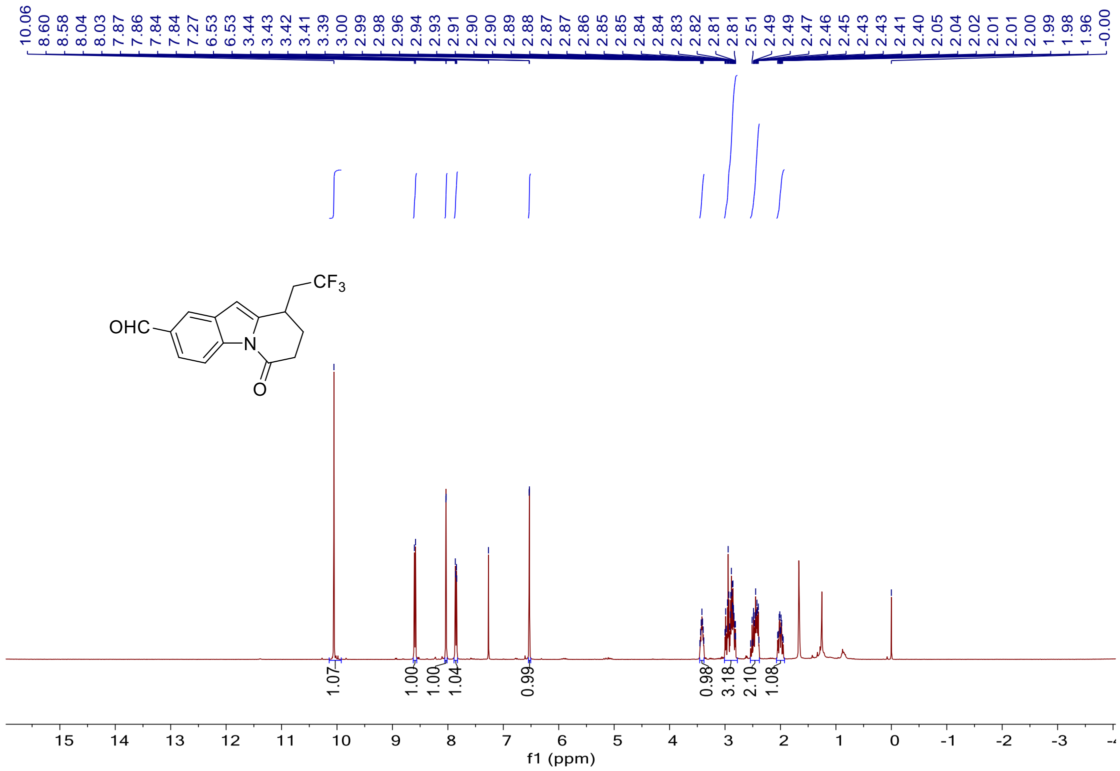

^{13}C NMR (101 MHz, $CDCl_3$)

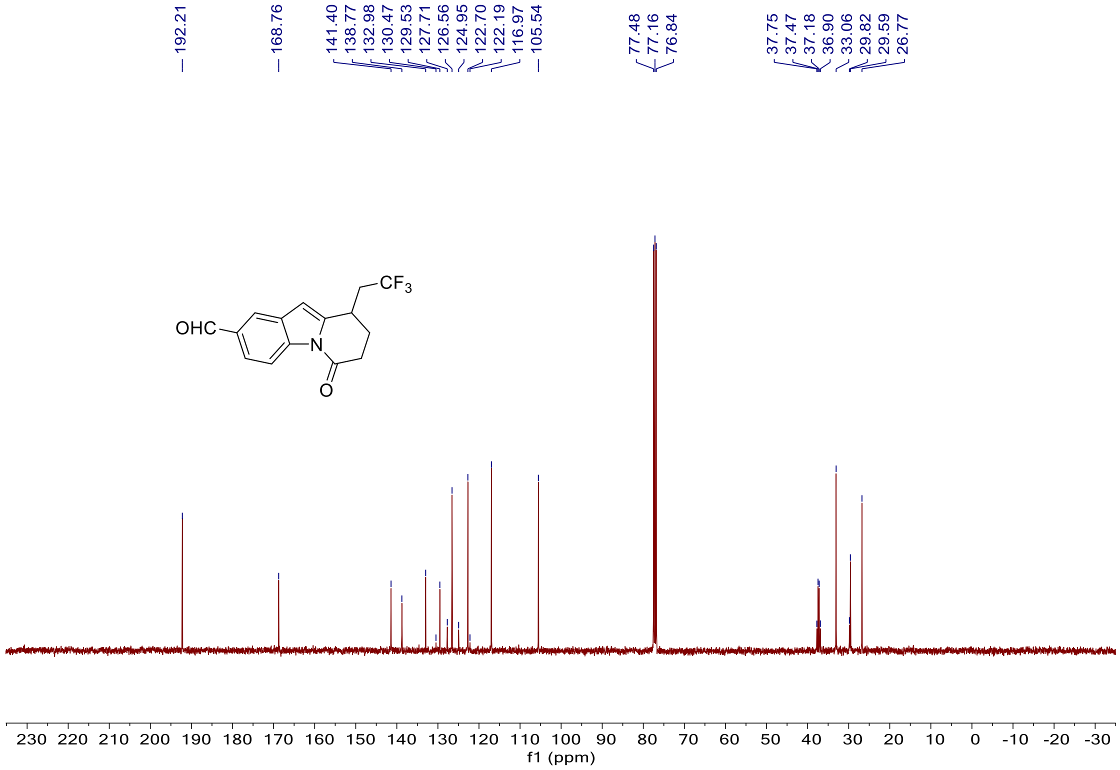

^{19}F NMR (376 MHz, $CDCl_3$)

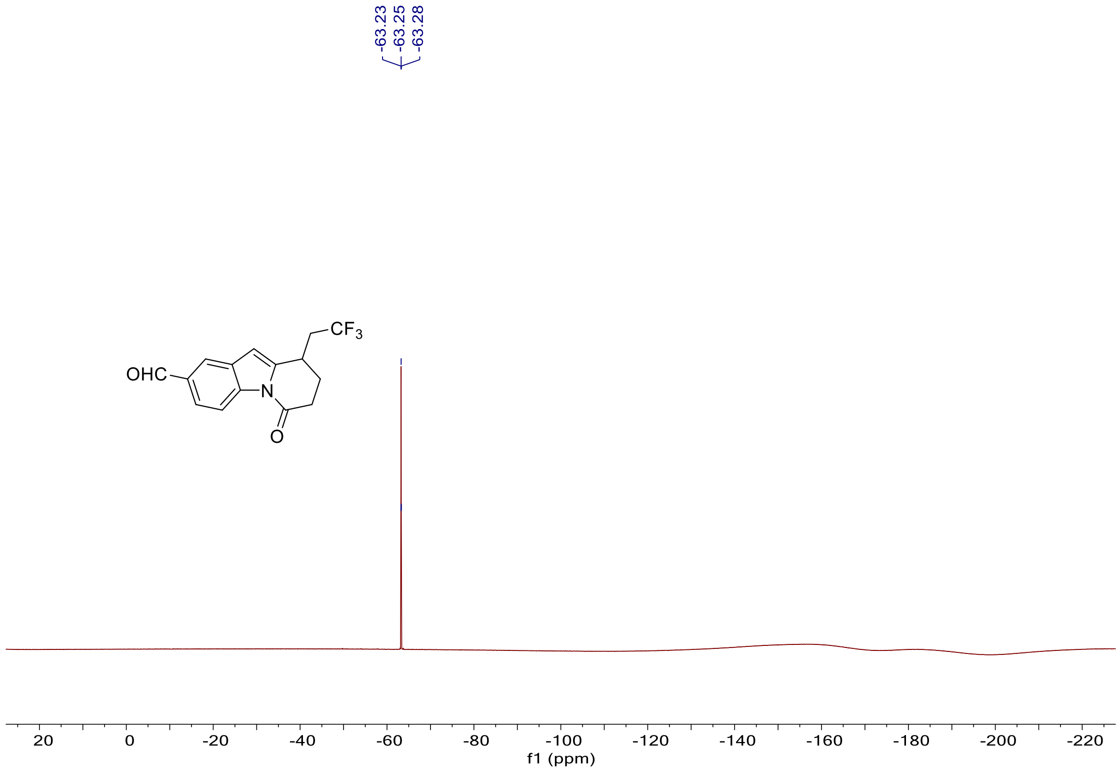

3k ^{1}H NMR (400 MHz, $CDCl_3$)

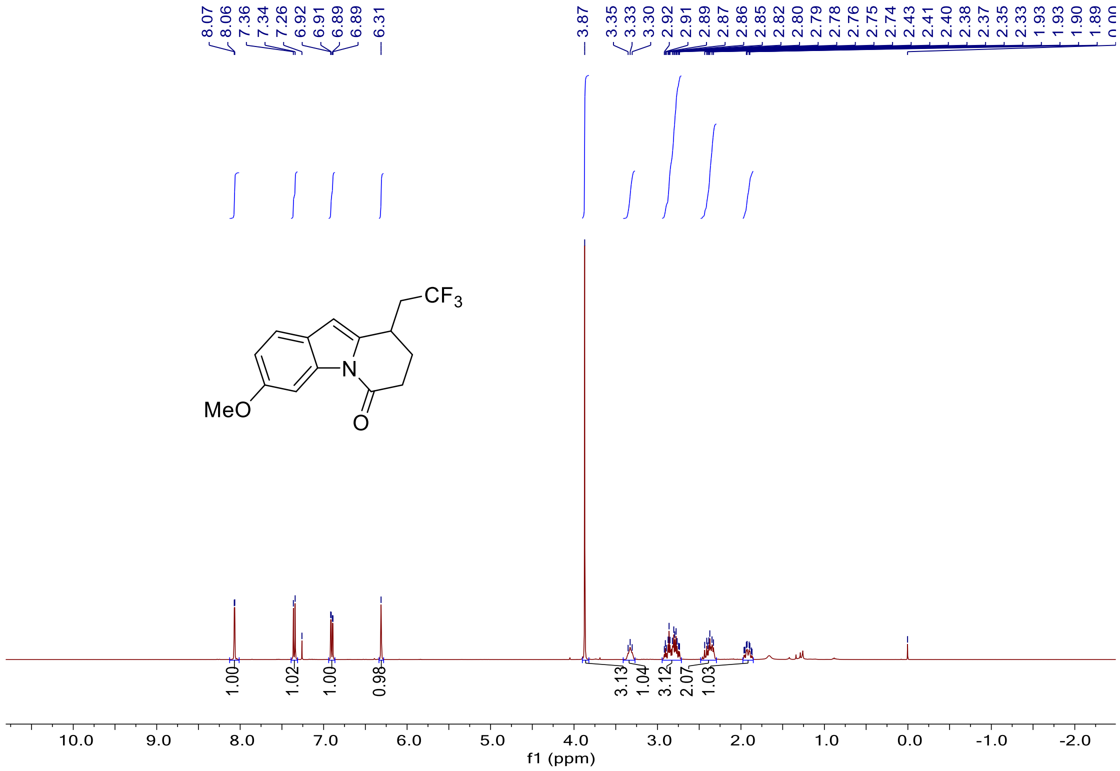

^{13}C NMR (101 MHz, $CDCl_3$)

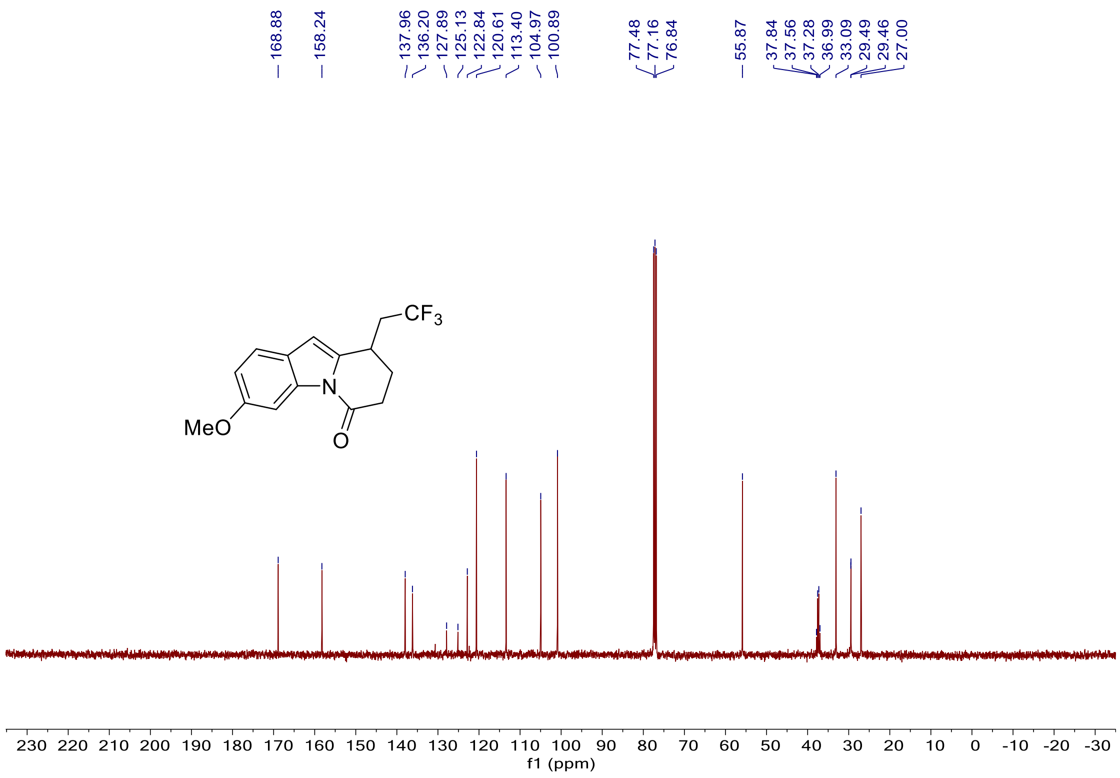

^{19}F NMR (376 MHz, $CDCl_3$)

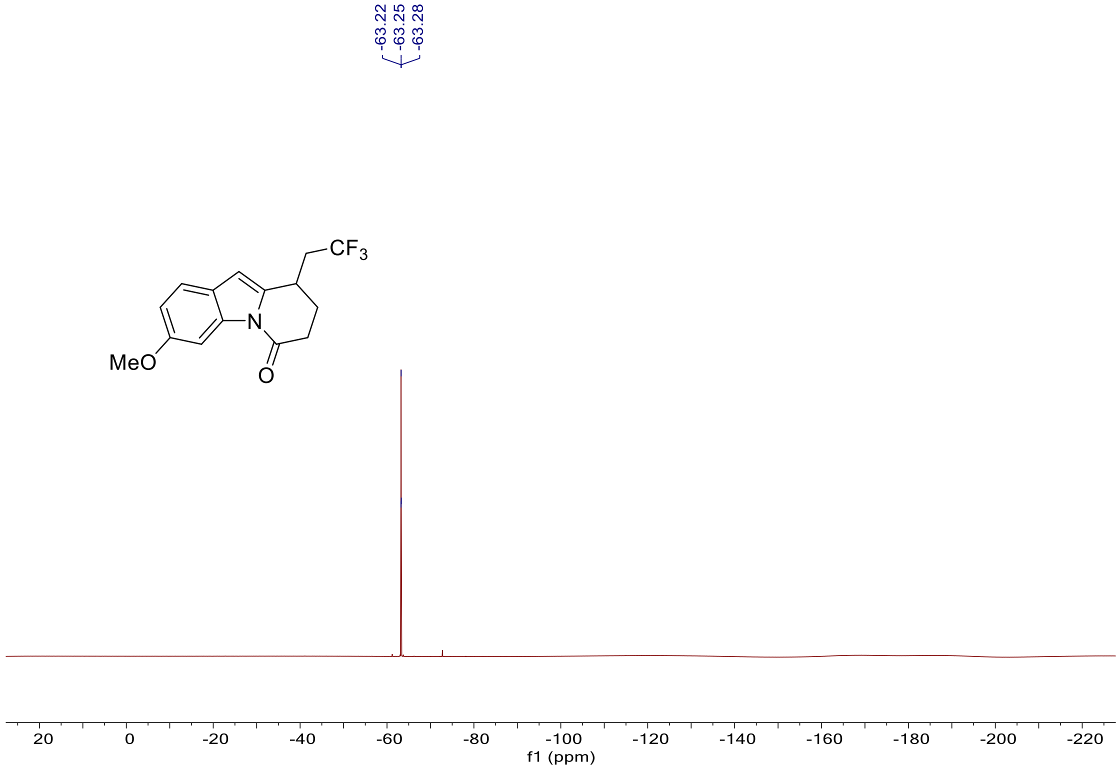

3l ^{1}H NMR (400 MHz, $CDCl_3$)

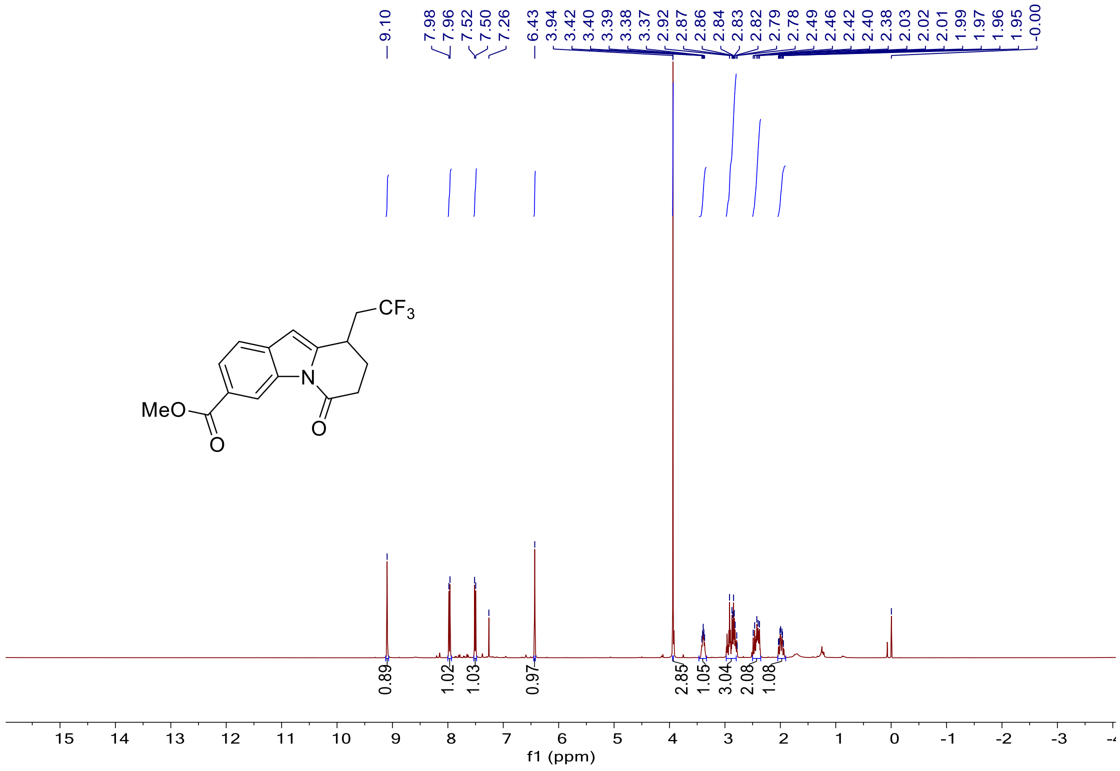

^{13}C NMR (101 MHz, $CDCl_3$)

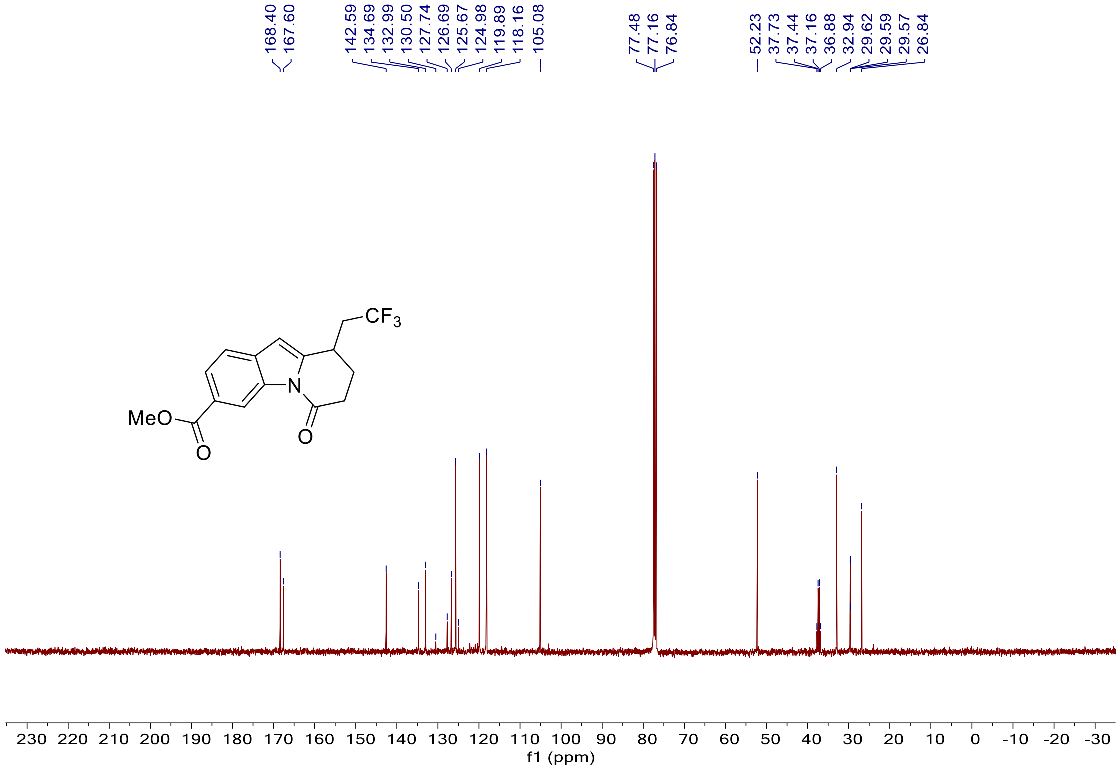

^{19}F NMR (376 MHz, $CDCl_3$)

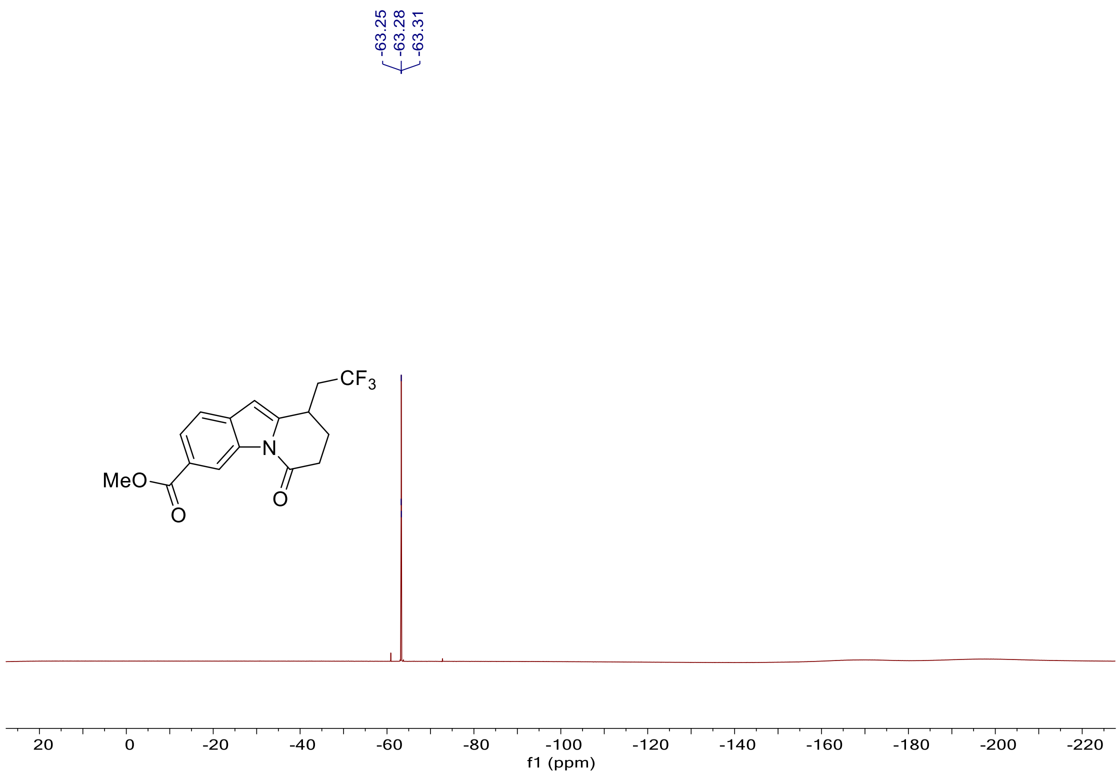

3m ^{1}H NMR (400 MHz, $CDCl_3$)

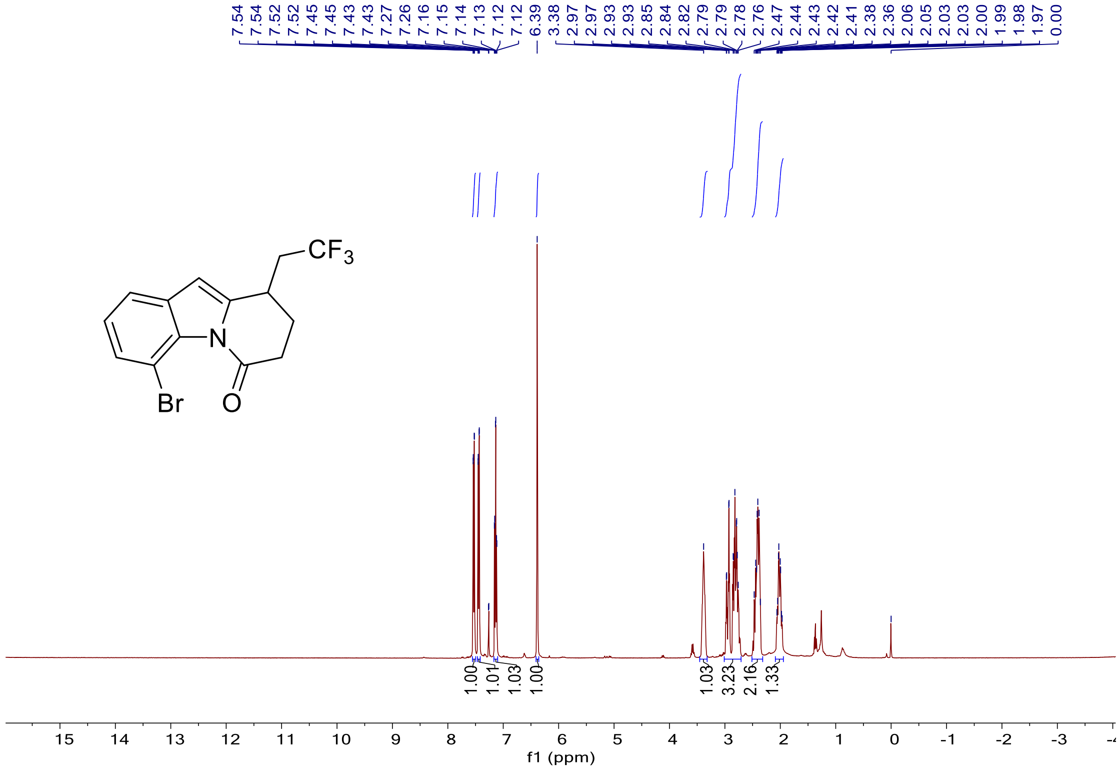

^{13}C NMR (101 MHz, $CDCl_3$)

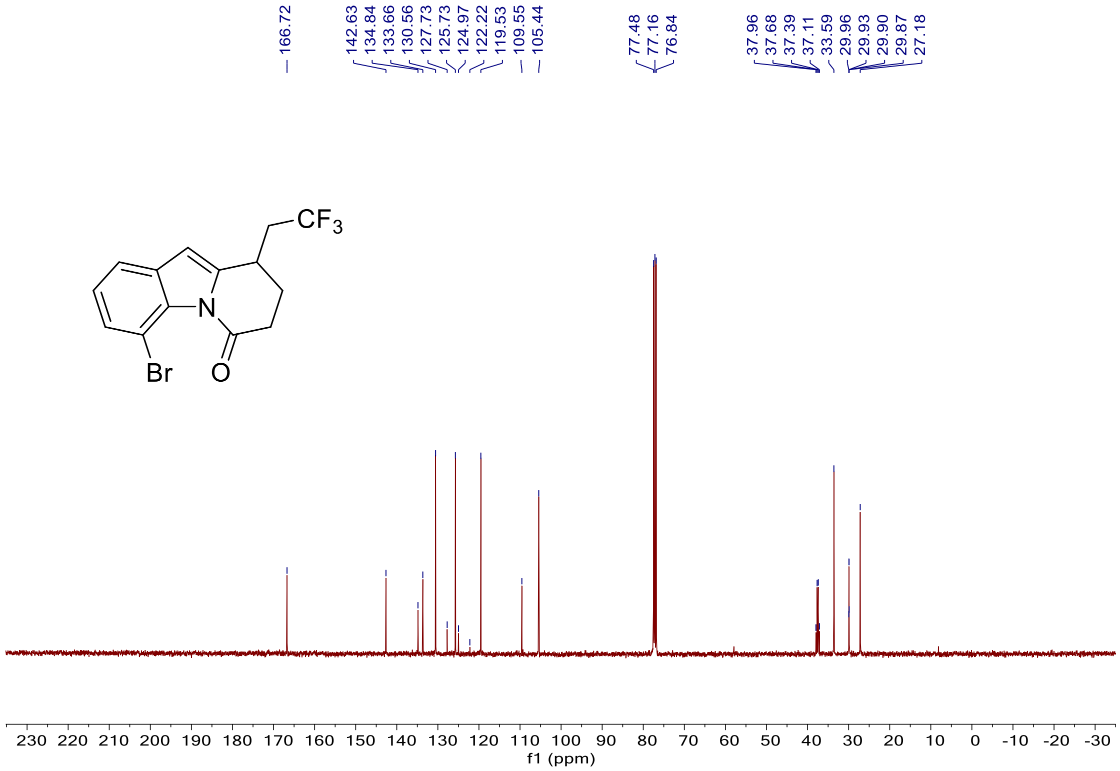

^{19}F NMR (376 MHz, $CDCl_3$)

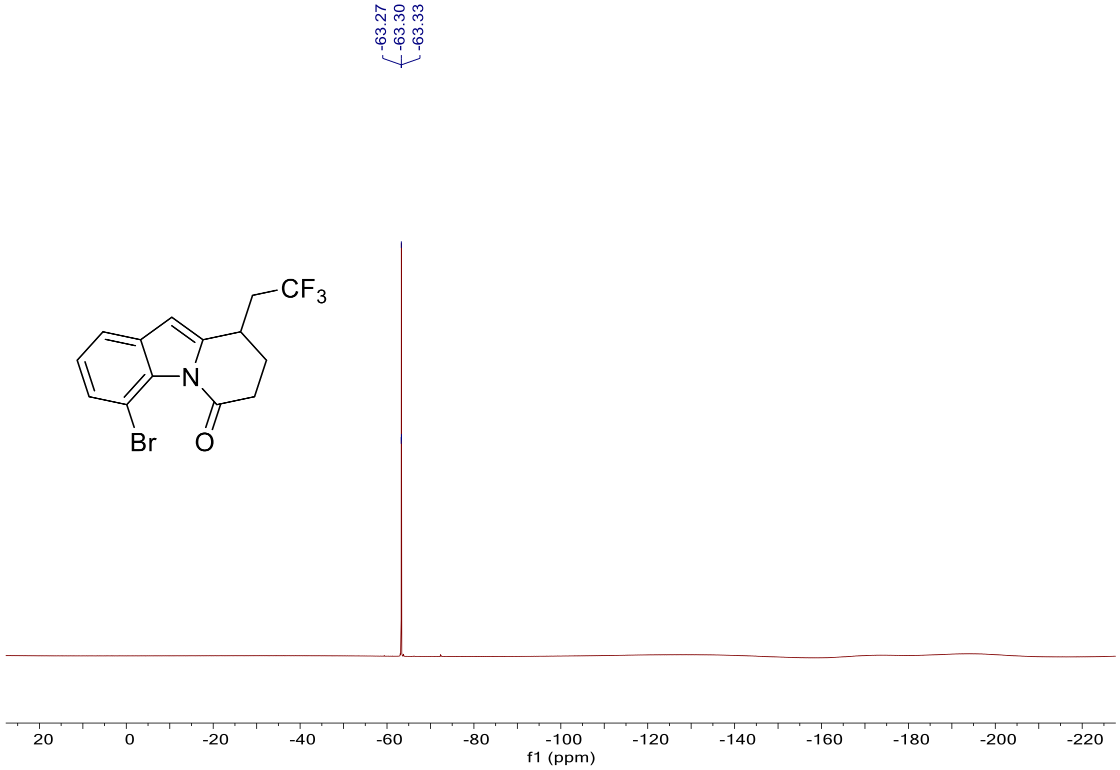

3n ^{1}H NMR (400 MHz, $CDCl_3$)

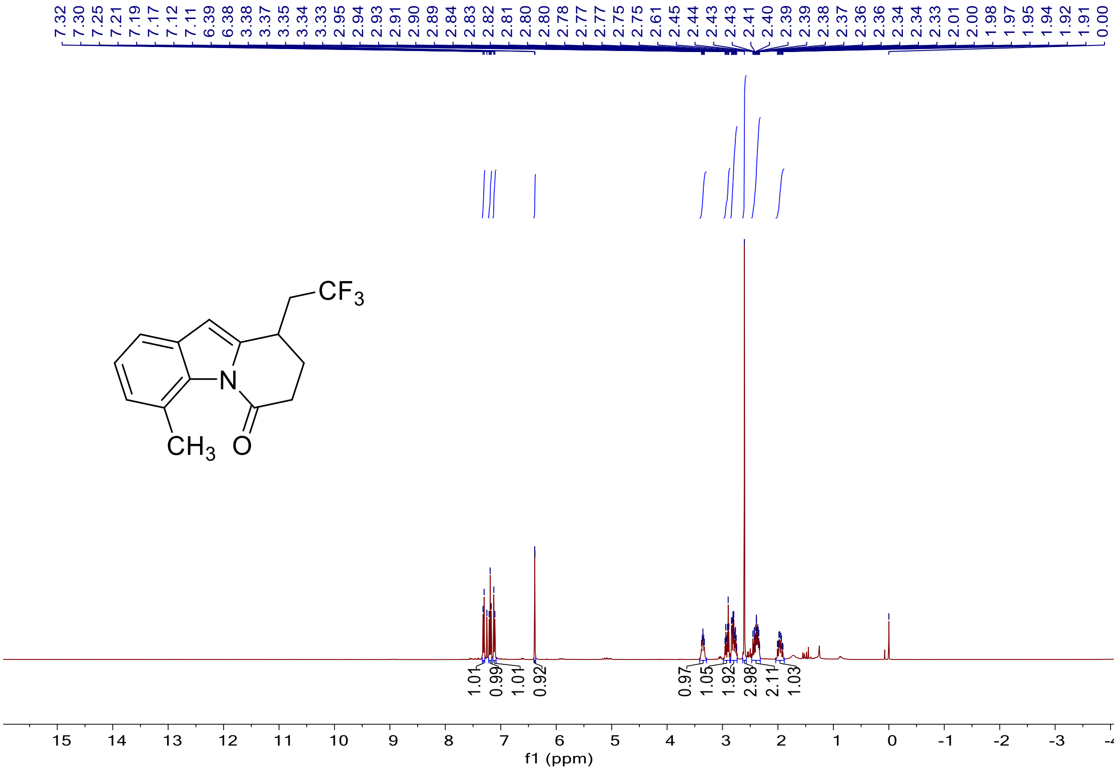

^{13}C NMR (101 MHz, $CDCl_3$)

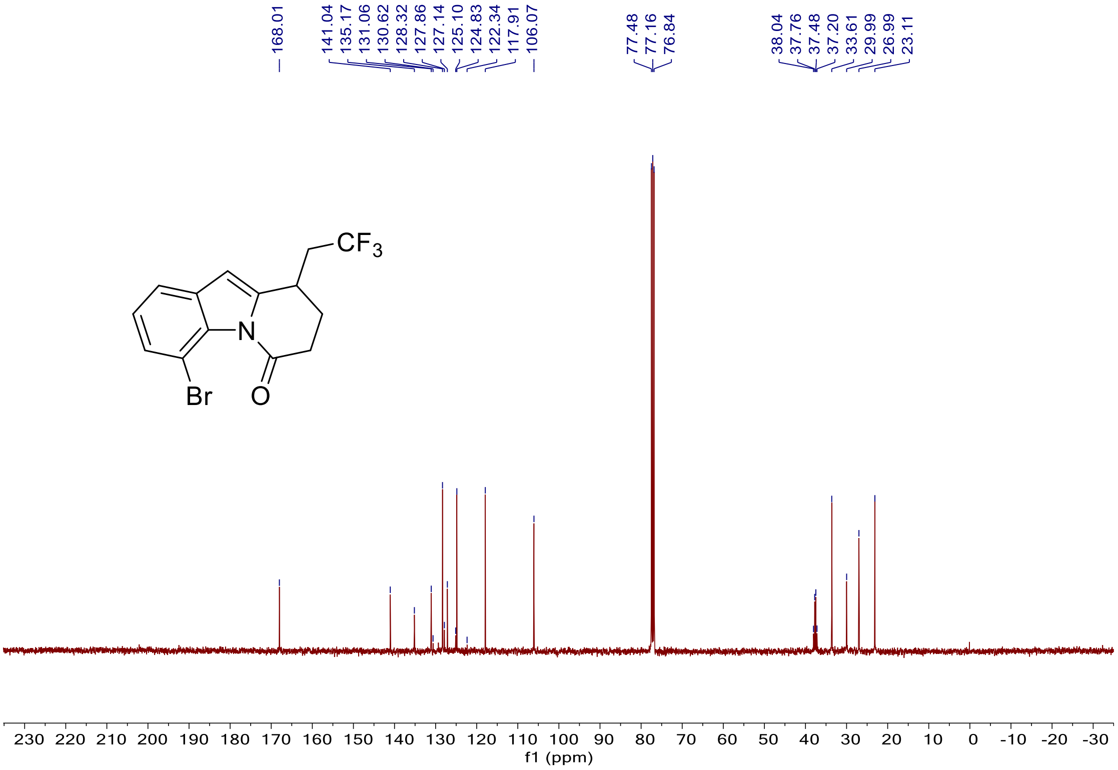

^{19}F NMR (376 MHz, $CDCl_3$)

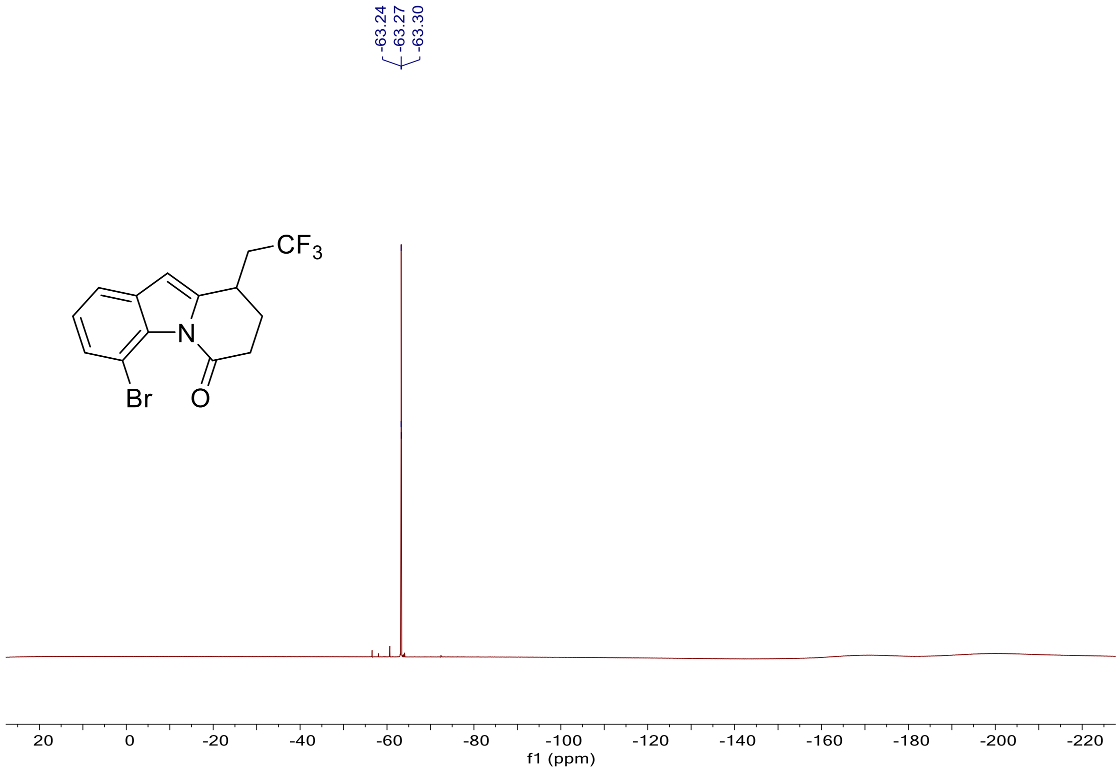

3o ^{1}H NMR (400 MHz, $CDCl_3$)

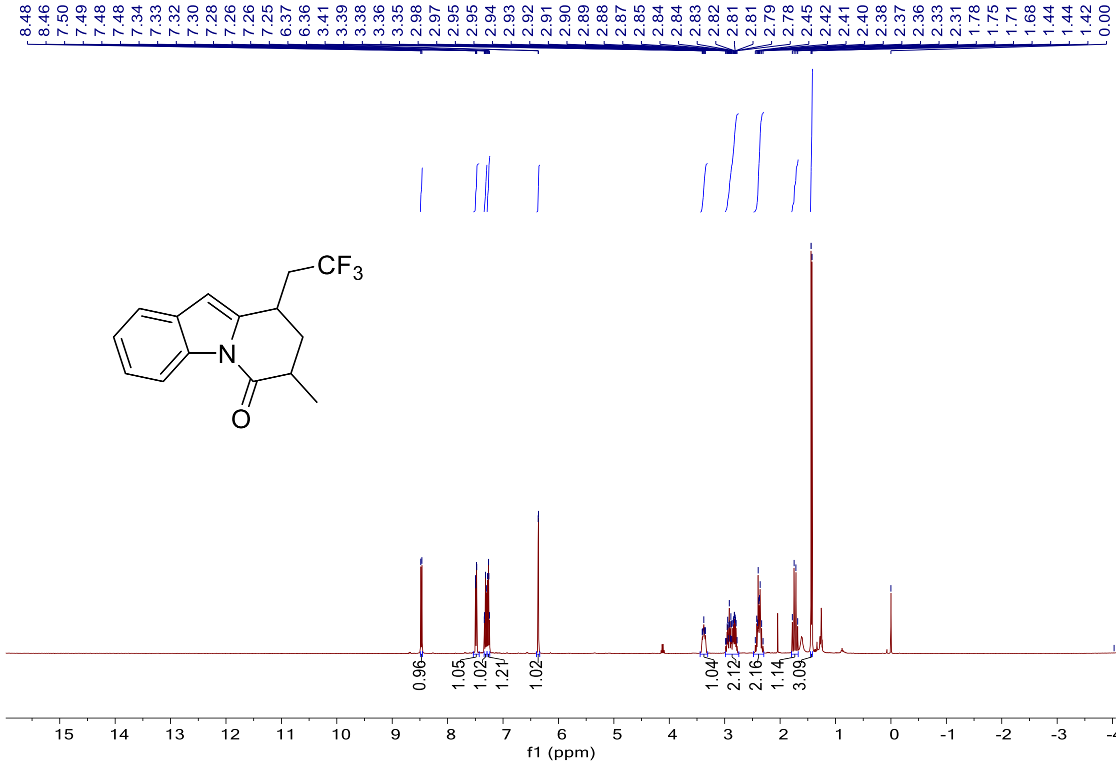

^{13}C NMR (101 MHz, $CDCl_3$)

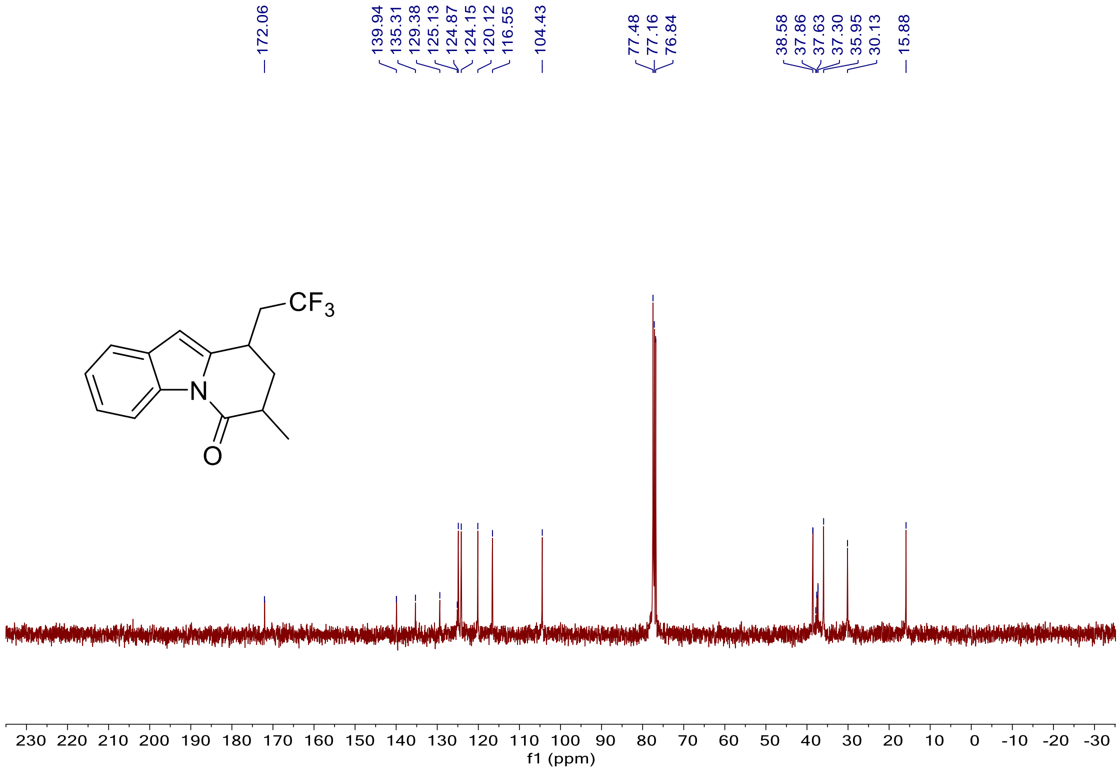

^{19}F NMR (376 MHz, $CDCl_3$)

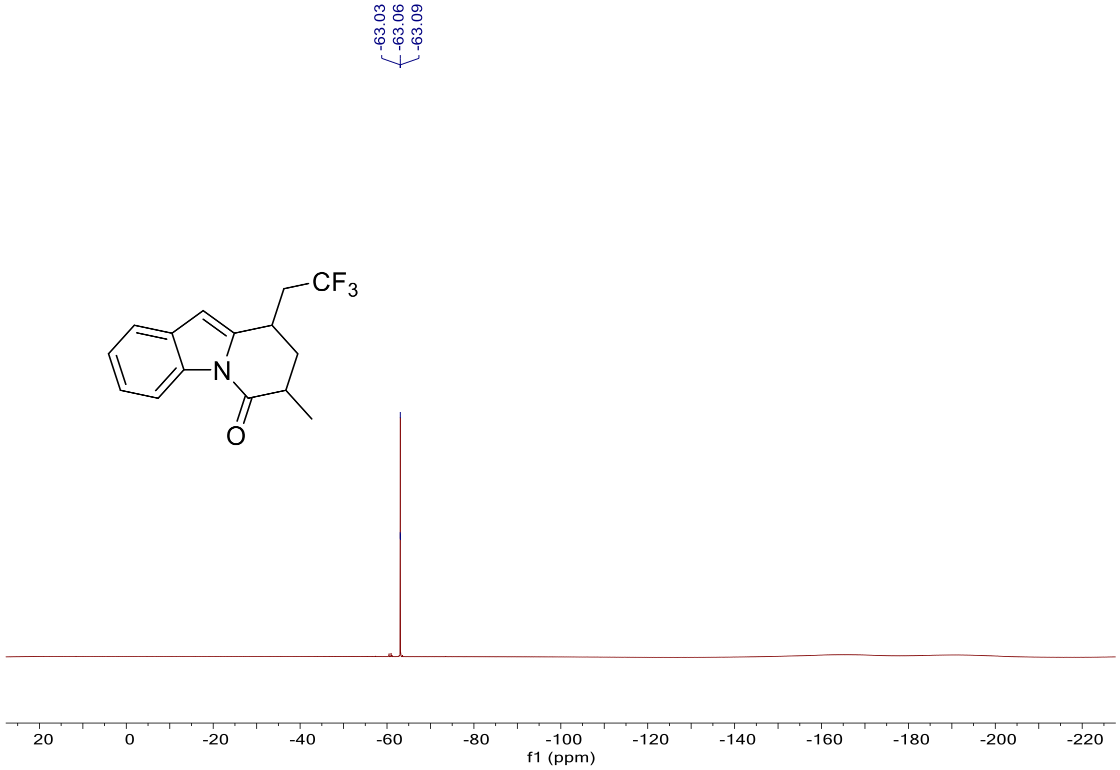

3p ^{1}H NMR (400 MHz, $CDCl_3$)

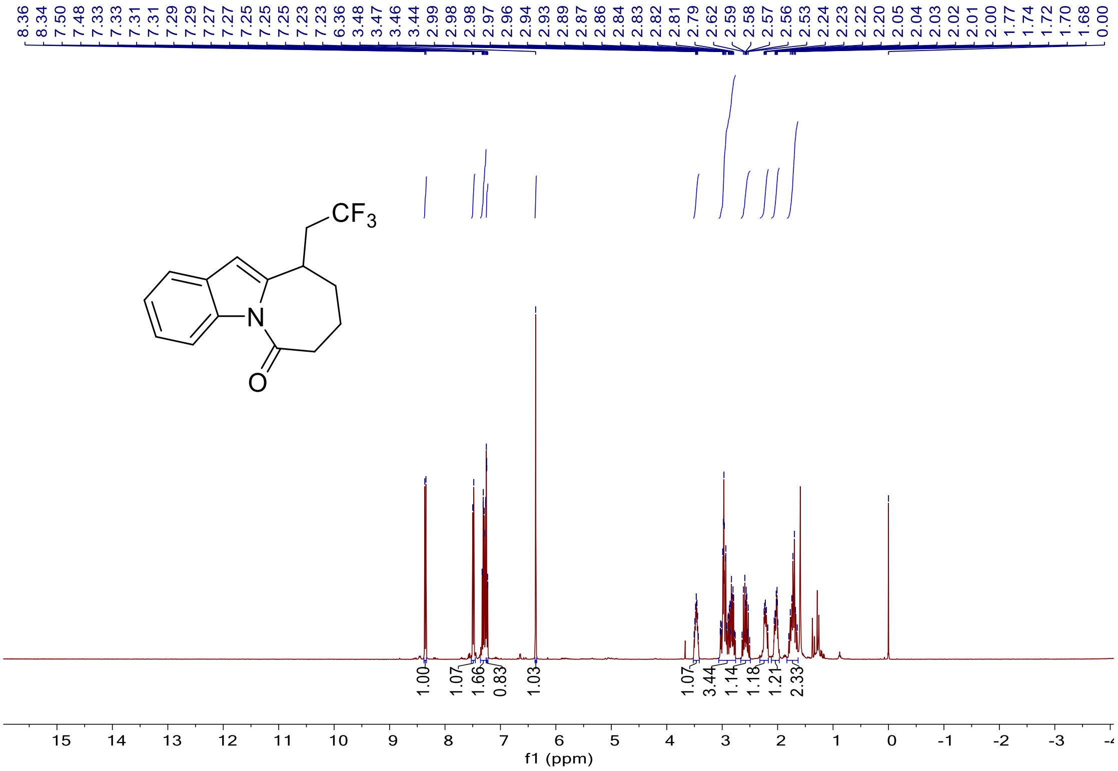

^{13}C NMR (101 MHz, $CDCl_3$)

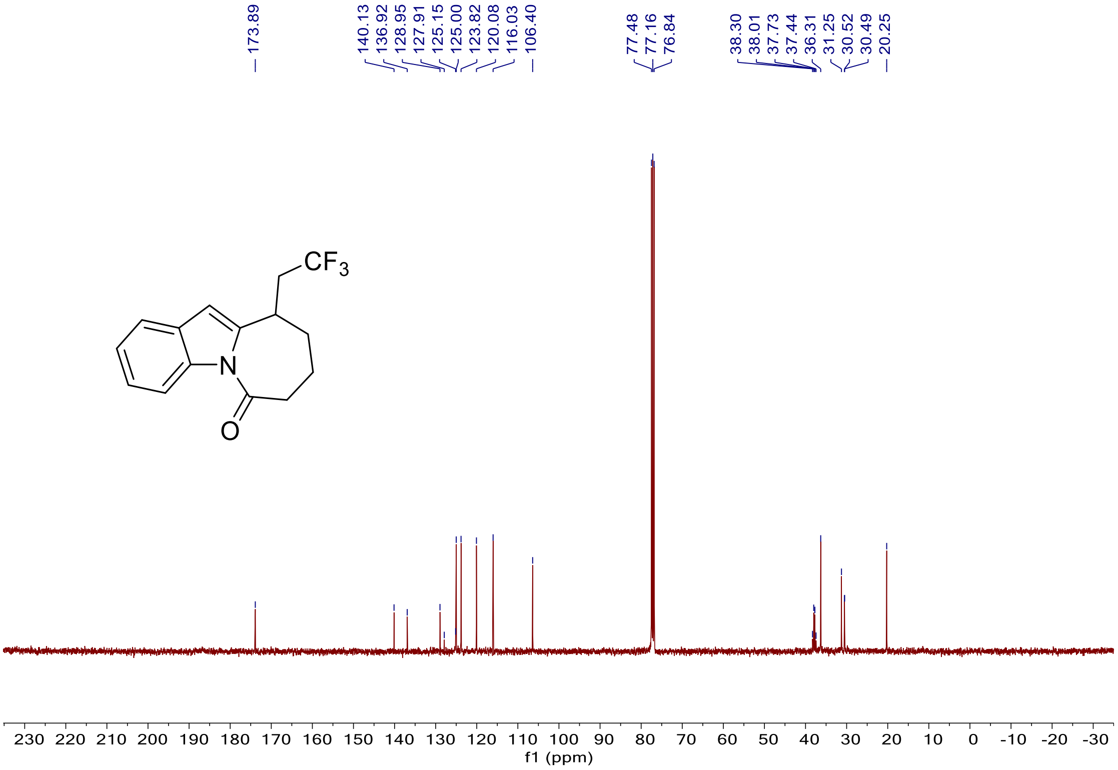

^{19}F NMR (376 MHz, $CDCl_3$)

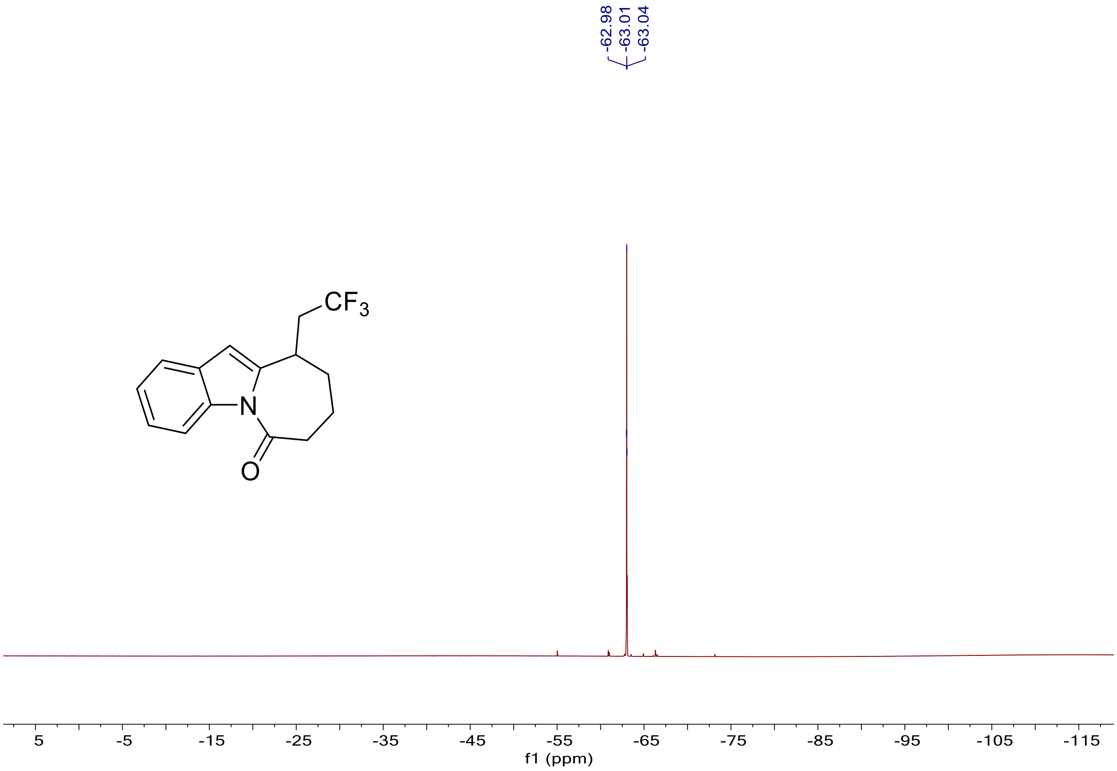

3q ^{1}H NMR (400 MHz, $CDCl_3$)

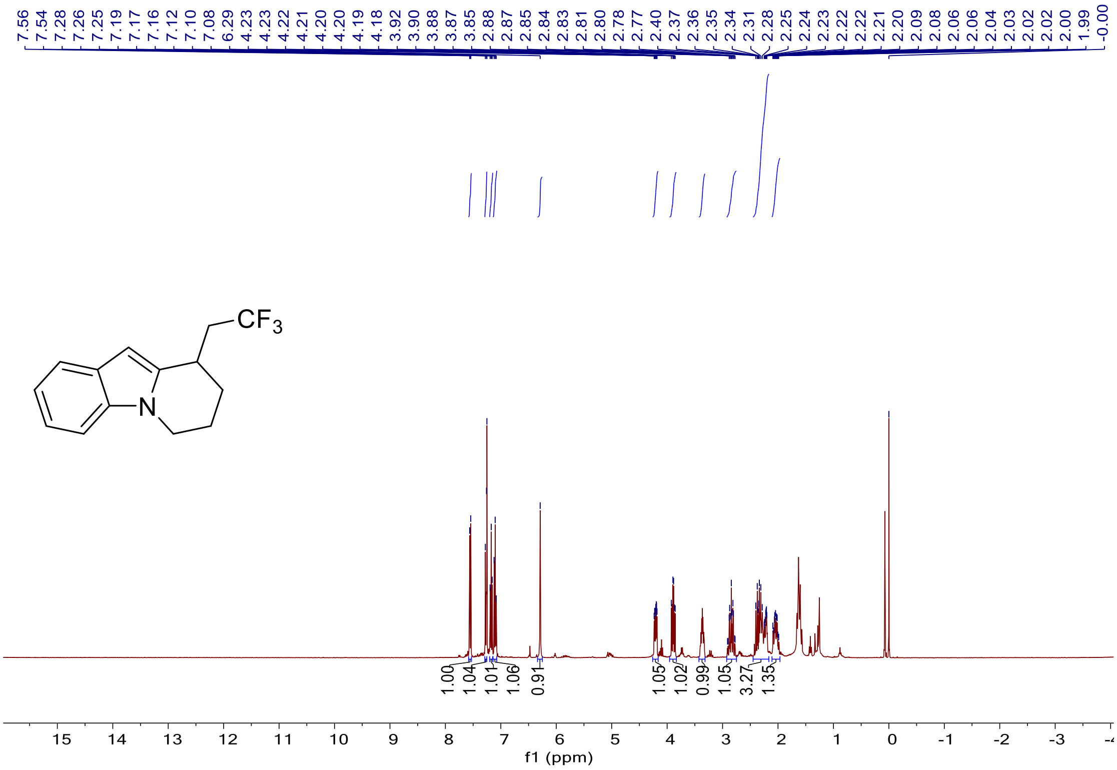

^{13}C NMR (101 MHz, $CDCl_3$)

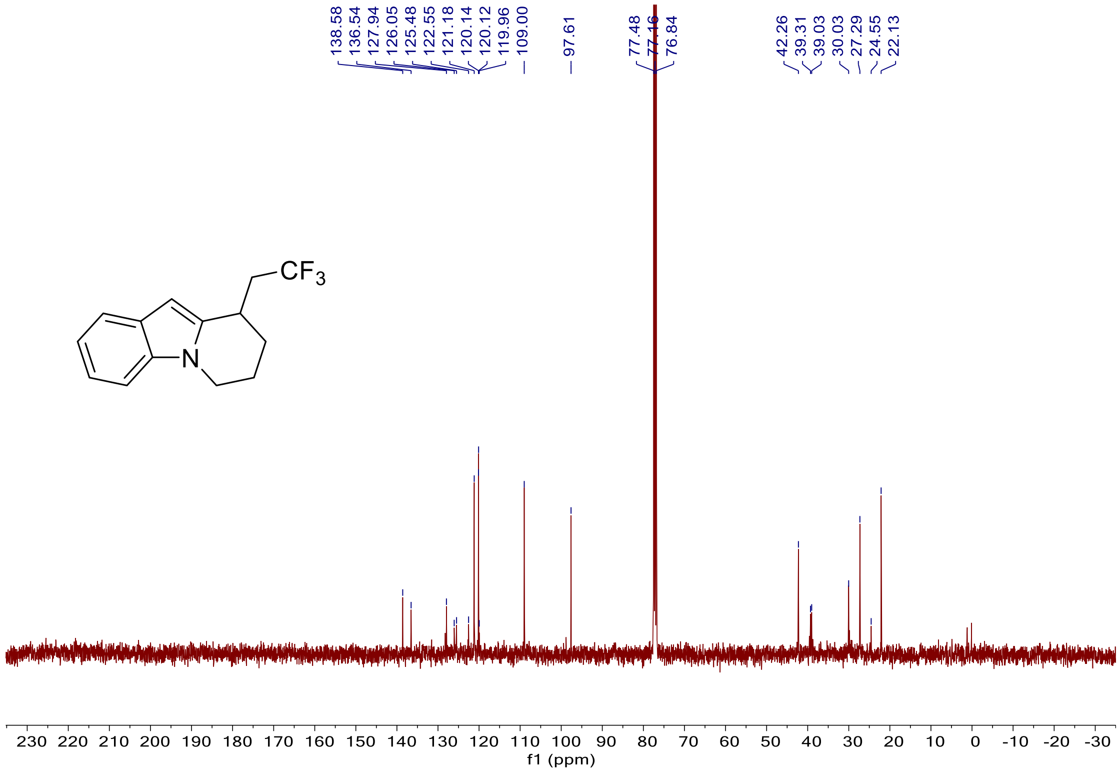

^{19}F NMR (376 MHz, $CDCl_3$)

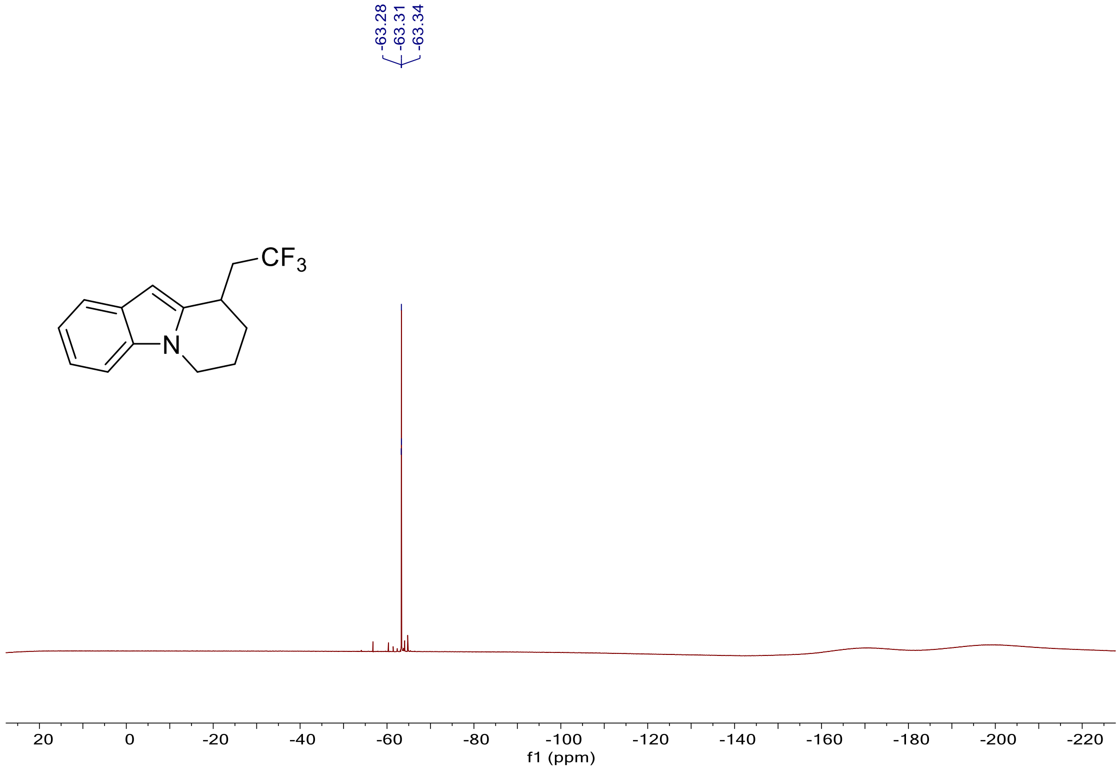

3r ^{1}H NMR (400 MHz, $CDCl_3$)

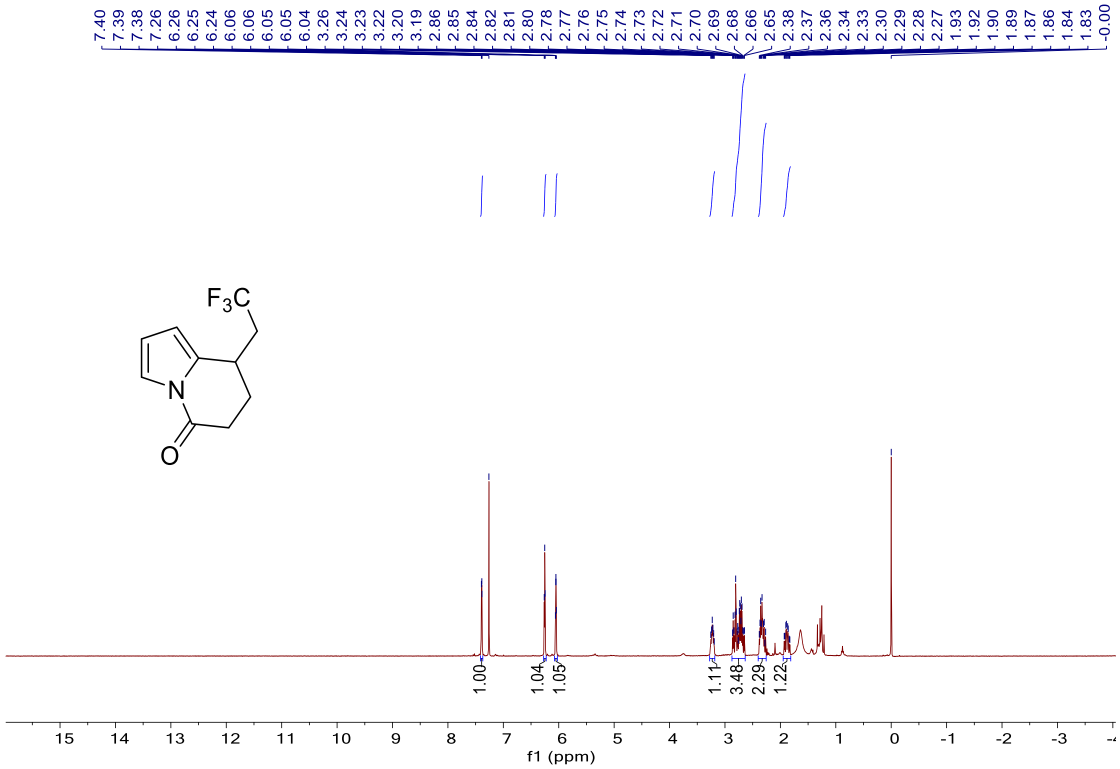

^{13}C NMR (101 MHz, $CDCl_3$)

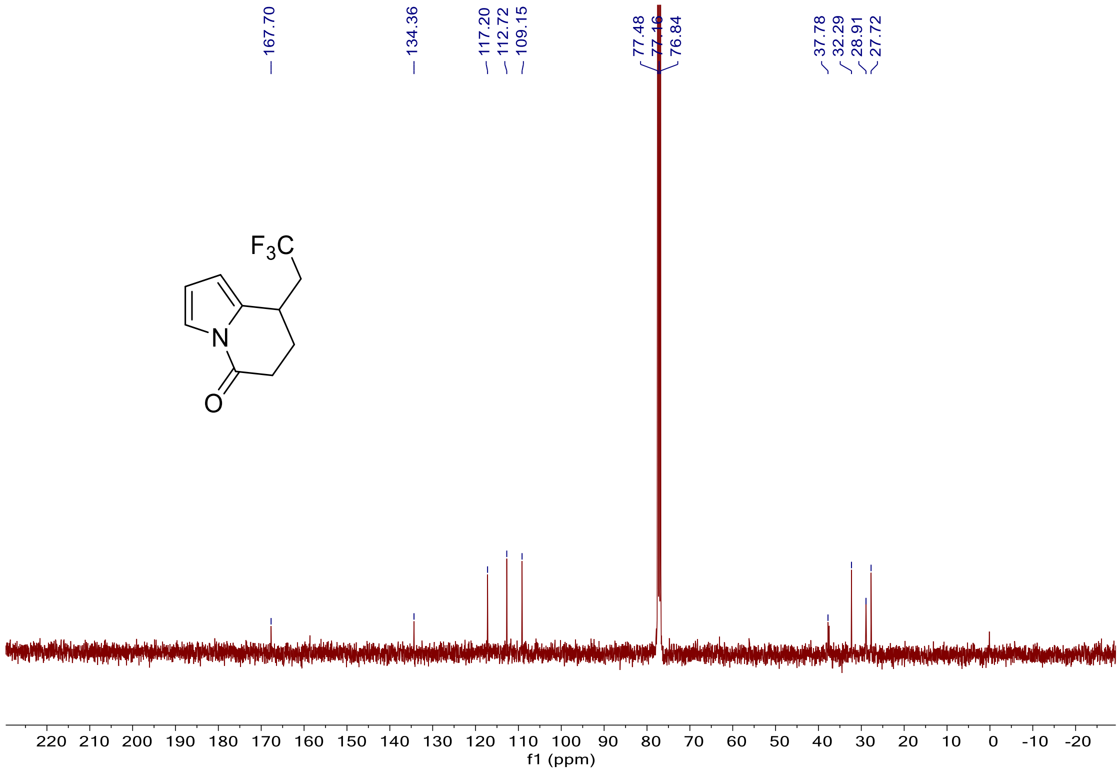

^{19}F NMR (376 MHz, $CDCl_3$)

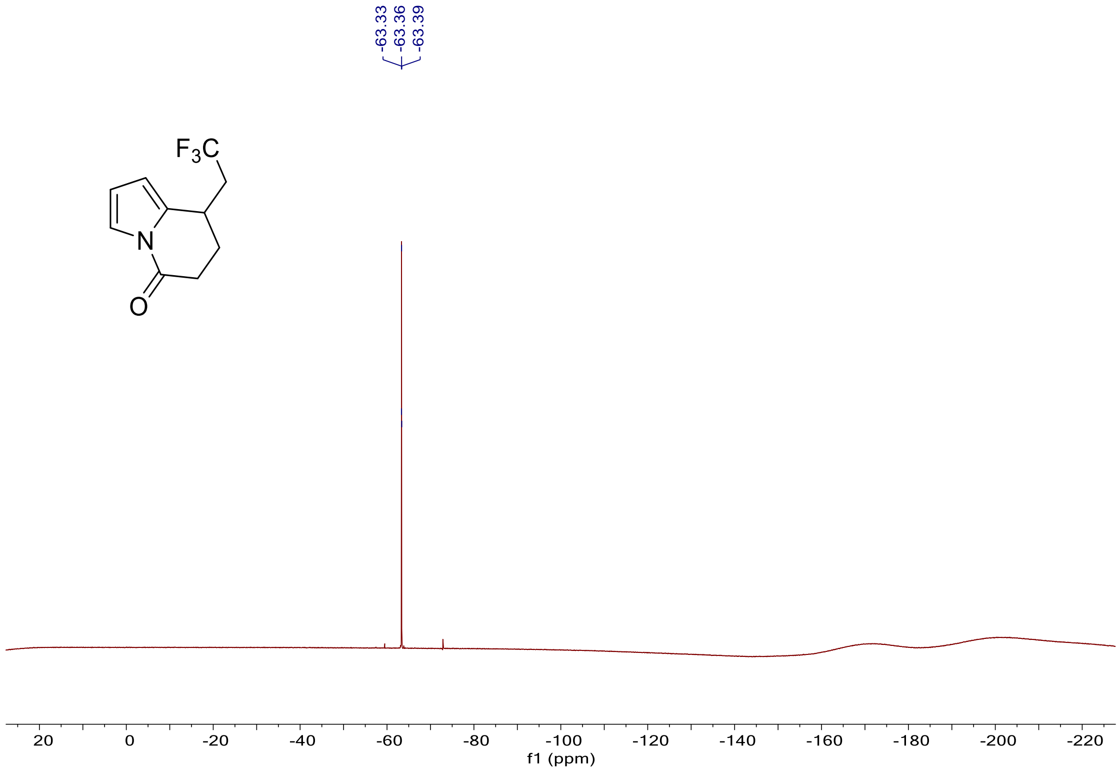

3s ^{1}H NMR (400 MHz, $CDCl_3$)

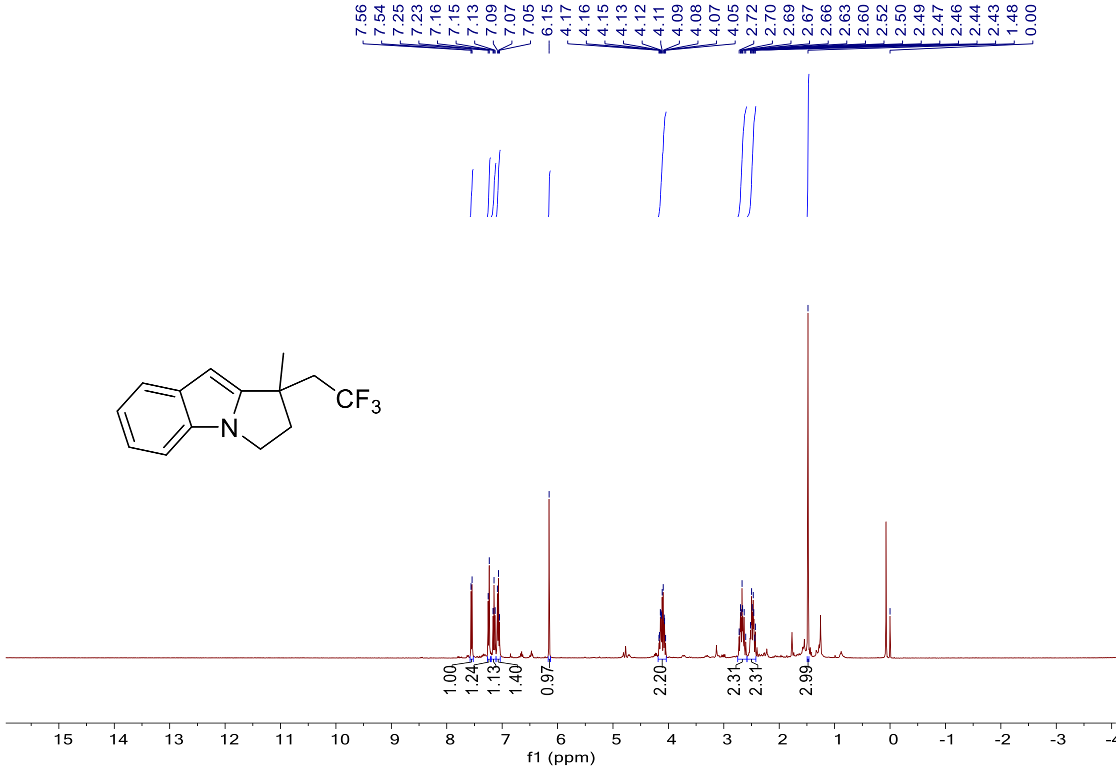

^{13}C NMR (101 MHz, $CDCl_3$)

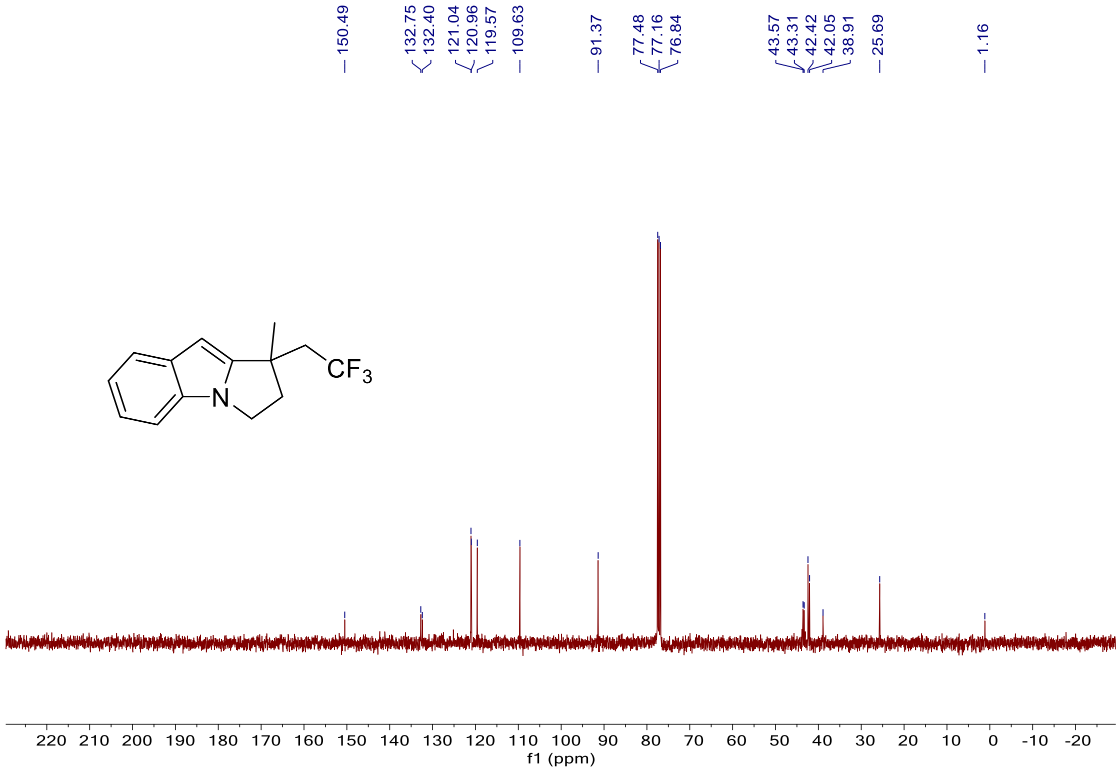

^{19}F NMR (376 MHz, $CDCl_3$)

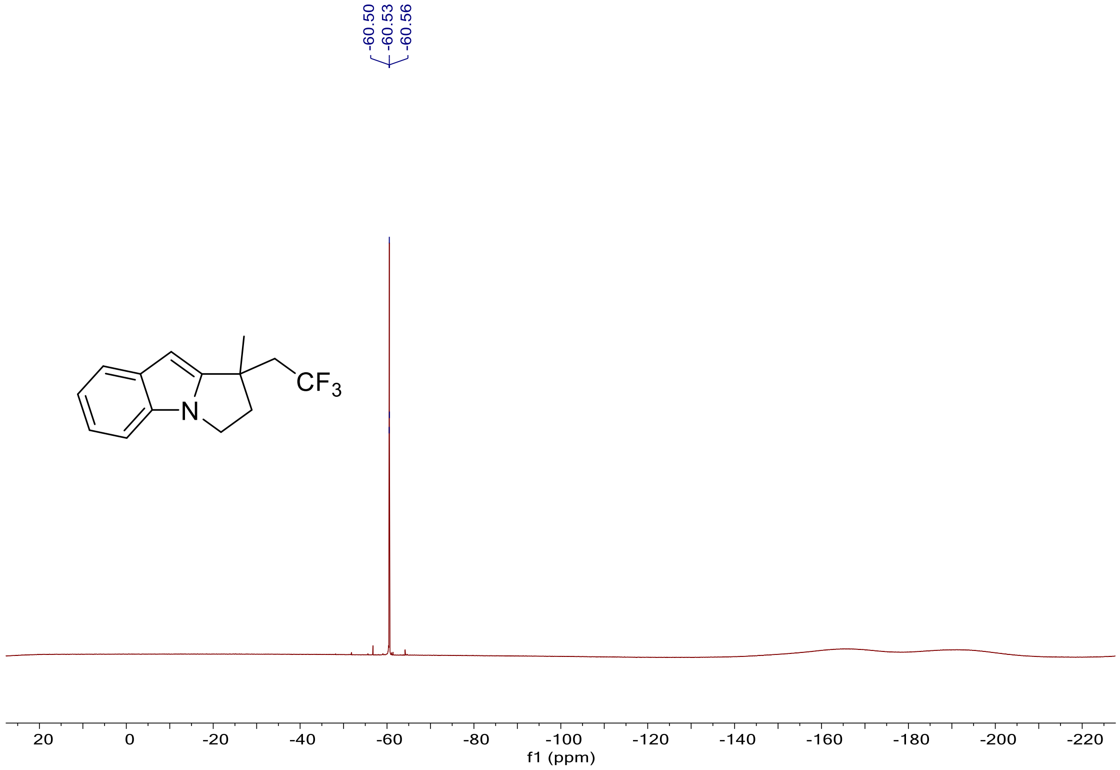

3t ^{1}H NMR (400 MHz, $CDCl_3$)

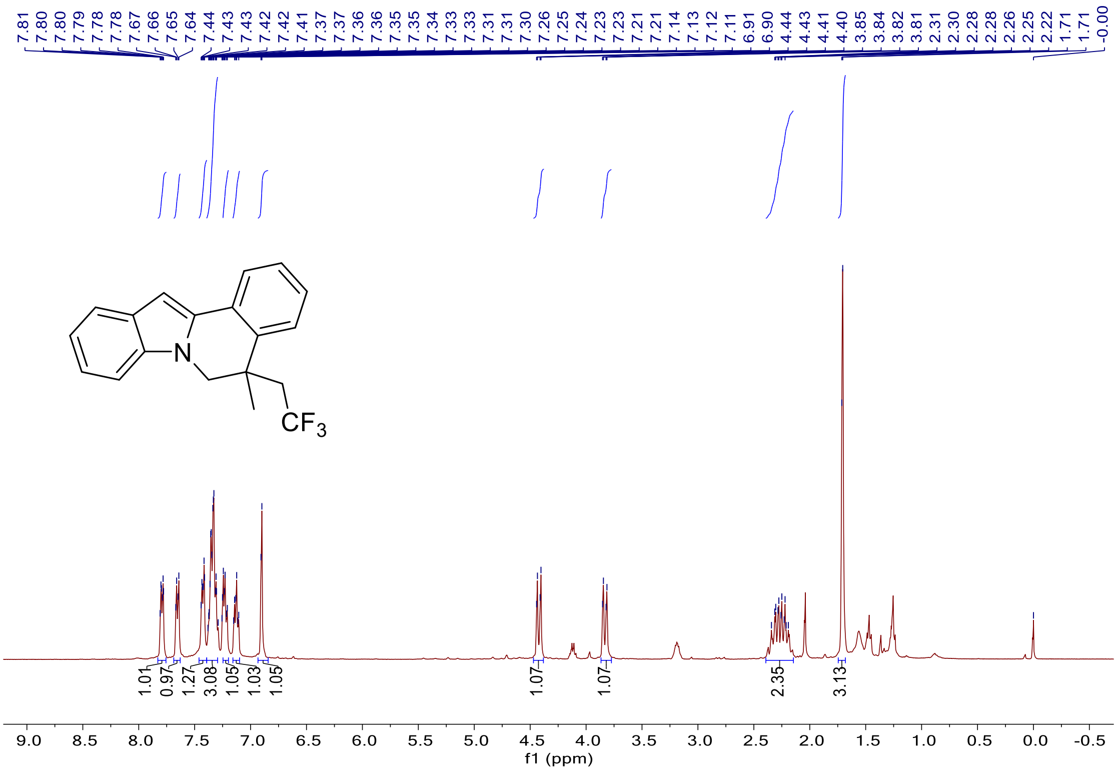

^{13}C NMR (101 MHz, $CDCl_3$)

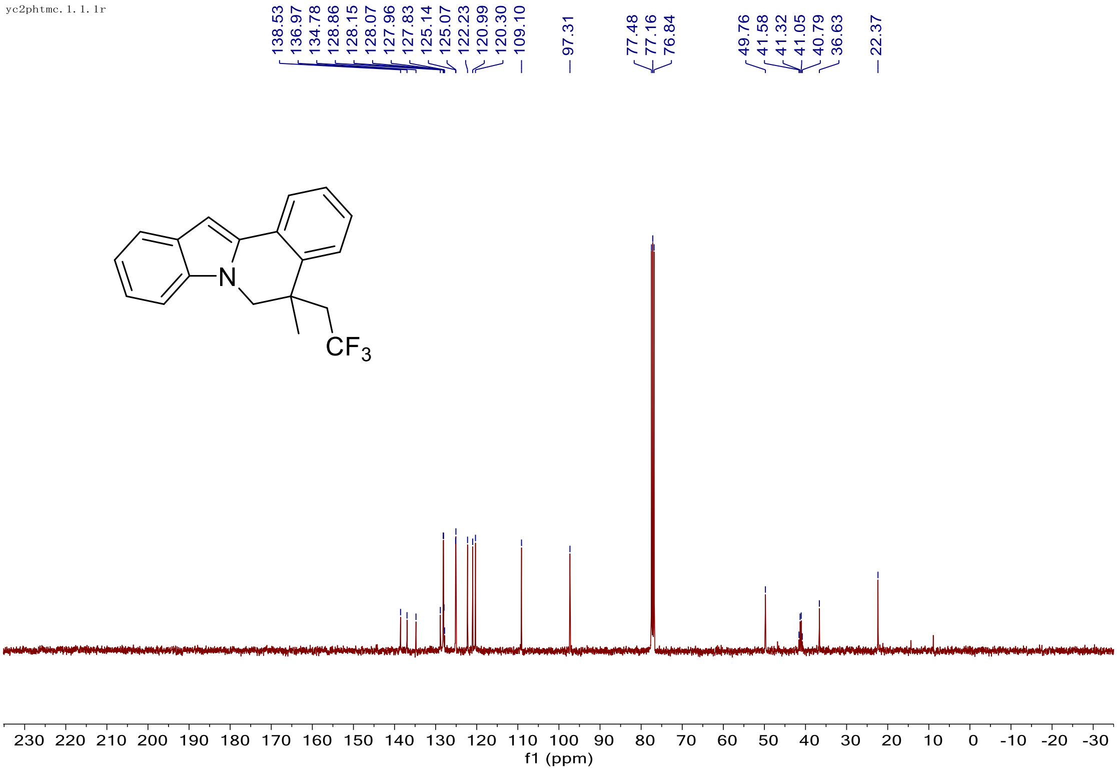

^{19}F NMR (376 MHz, $CDCl_3$)

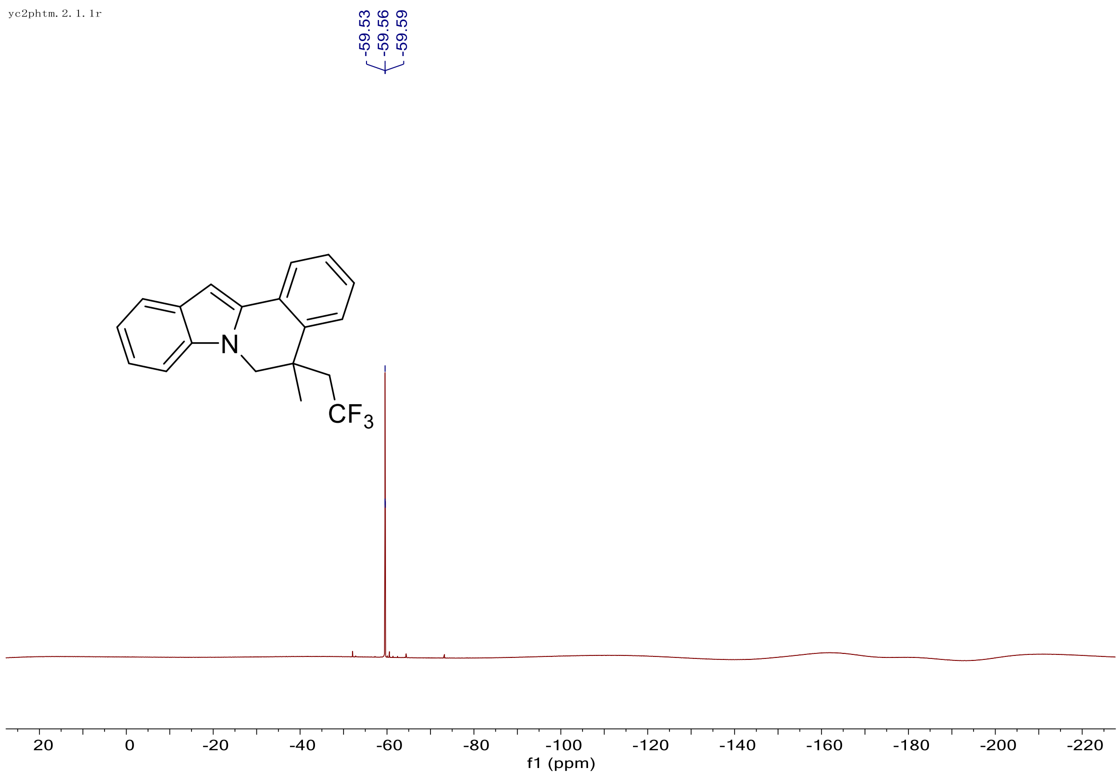

3u ^{1}H NMR (400 MHz, $CDCl_3$)

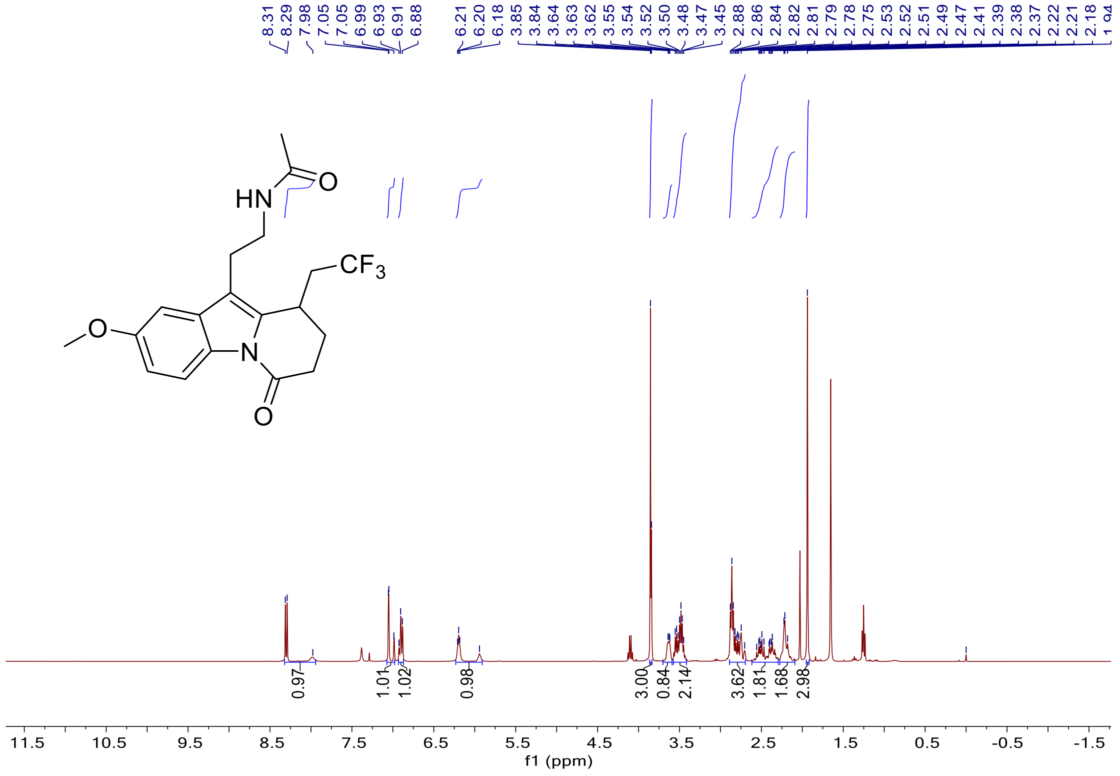

^{13}C NMR (101 MHz, $CDCl_3$)

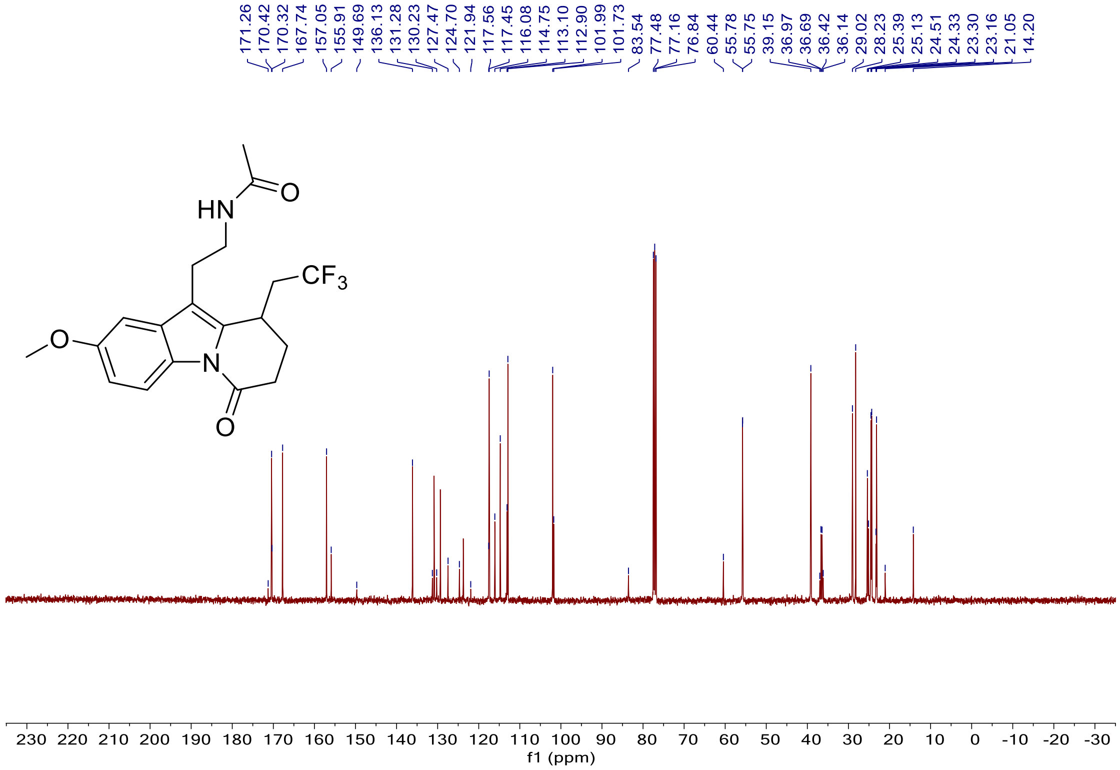

^{19}F NMR (376 MHz, $CDCl_3$)

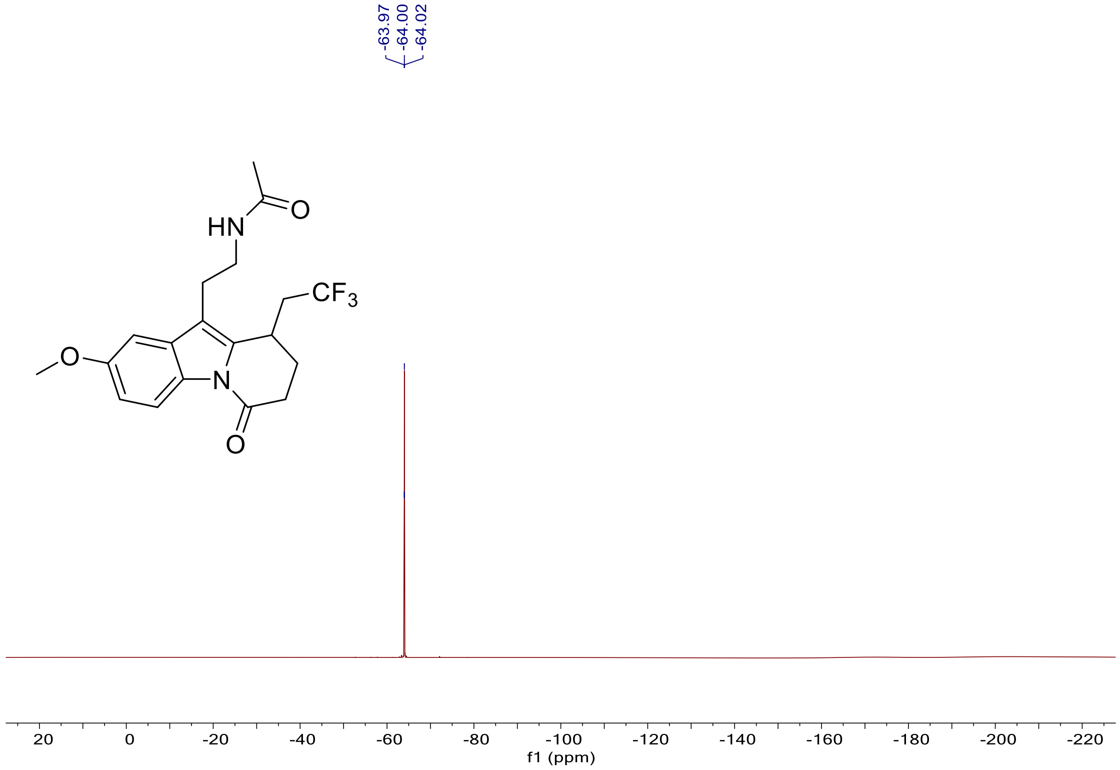

3v ^{1}H NMR (400 MHz, $CDCl_3$)

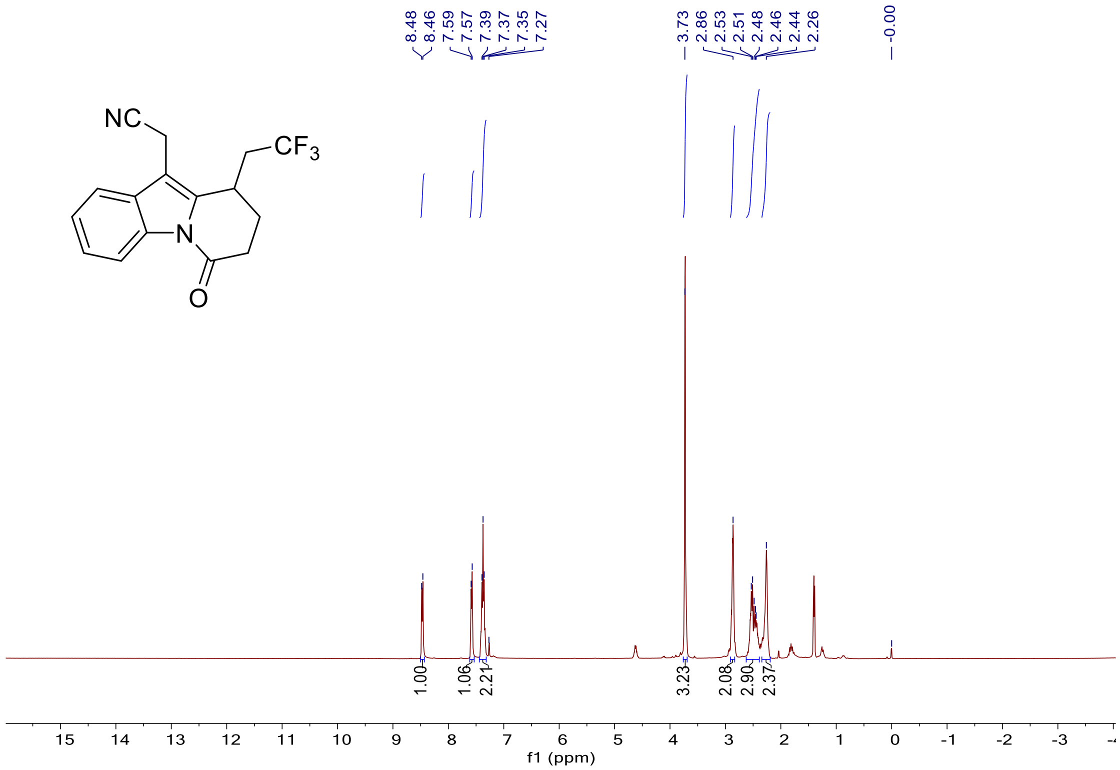

^{13}C NMR (101 MHz, $CDCl_3$)

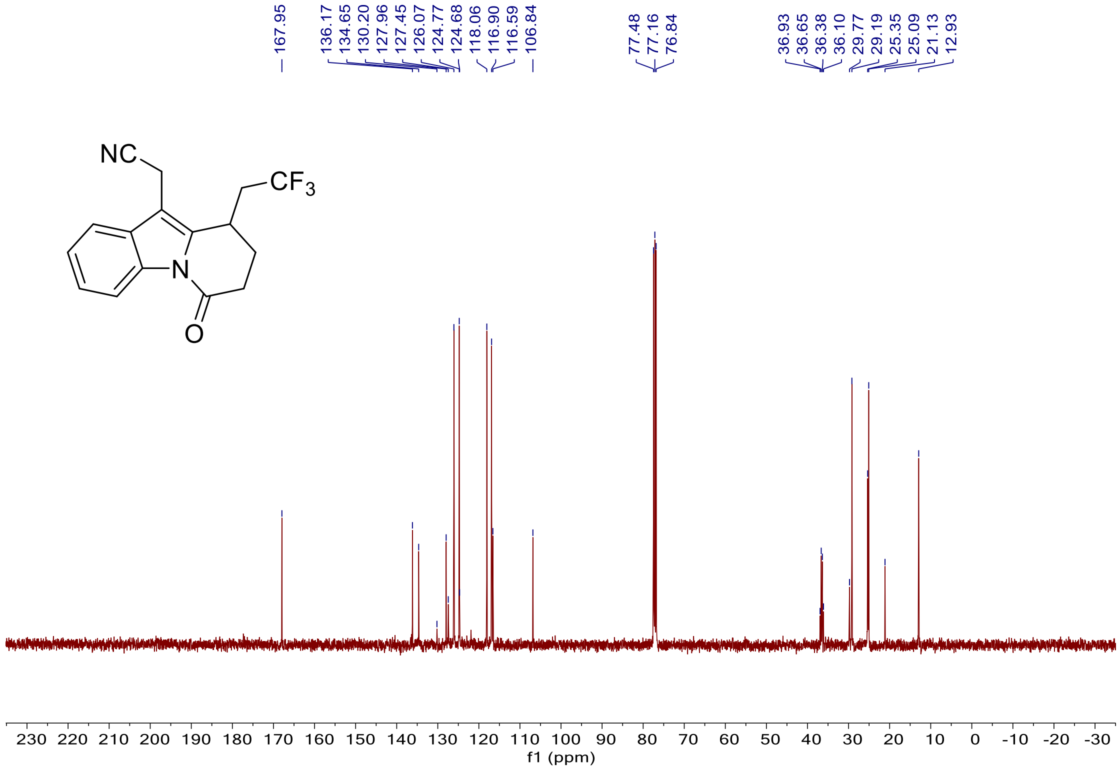

^{19}F NMR (376 MHz, $CDCl_3$)

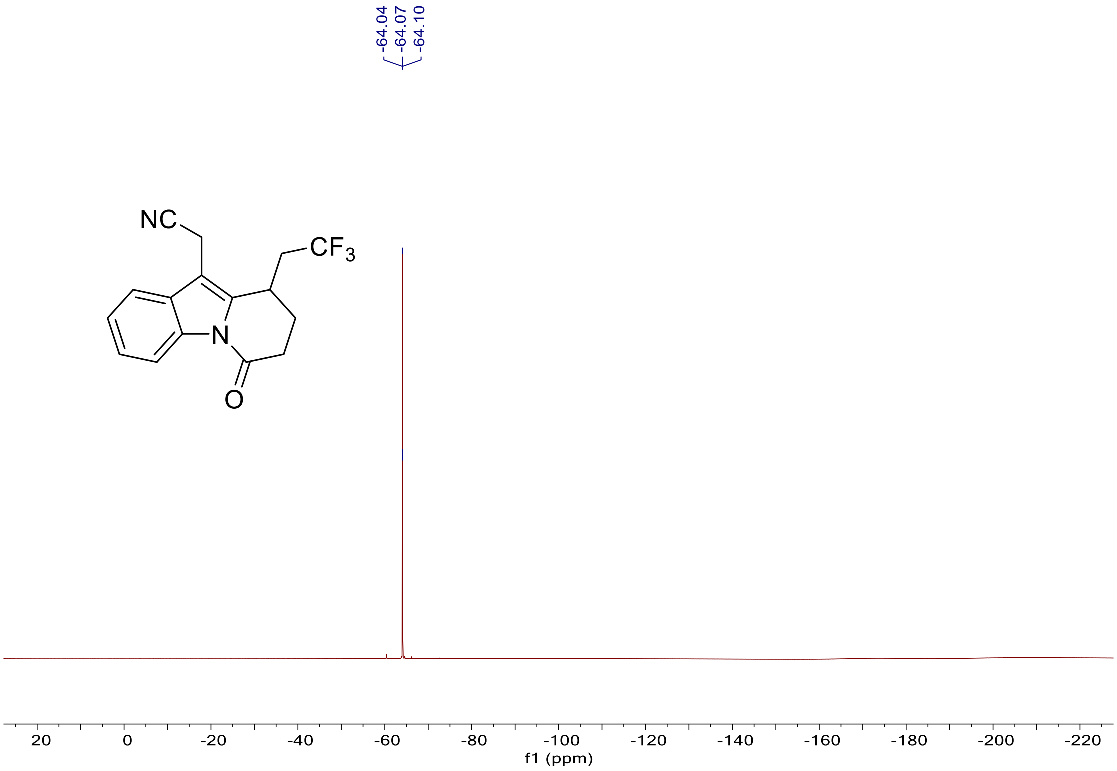

Supplement: File 1 — Characterization data and copies of spectra. [file Beilstein_J_Org_Chem-20-118-s001.pdf]
